# International Evidence Based Reappraisal of Genes Associated With Arrhythmogenic Right Ventricular Cardiomyopathy Using the Clinical Genome Resource Framework

**DOI:** 10.1161/CIRCGEN.120.003273

**Published:** 2021-04-08

**Authors:** Cynthia A. James, Jan D.H. Jongbloed, Ray E. Hershberger, Ana Morales, Daniel P. Judge, Petros Syrris, Kalliopi Pilichou, Argelia Medeiros Domingo, Brittney Murray, Julia Cadrin-Tourigny, Ronald Lekanne Deprez, Rudy Celeghin, Alexandros Protonotarios, Babken Asatryan, Emily Brown, Elizabeth Jordan, Jennifer McGlaughon, Courtney Thaxton, C. Lisa Kurtz, J. Peter van Tintelen

**Affiliations:** 1 Division of Cardiology, Department of Medicine, Johns Hopkins Hospital, Baltimore, MD (C.A.J., B.M., E.B.).; 2 Department of Genetics, University of Groningen, University Medical Center Groningen, the Netherlands (J.D.H.J.).; 3 Division of Cardiovascular Medicine, Department of Internal Medicine (R.E.H., E.J.), Ohio State University, Columbus.; 4 Division of Human Genetics, Department of Internal Medicine (R.E.H., A.M.), Ohio State University, Columbus.; 5 Division of Cardiology, Department of Medicine, Medical University of South Carolina, Charleston (D.P.J.).; 6 Centre for Heart Muscle Disease, Institute of Cardiovascular Science, University College London, United Kingdom (P.S., A.P.).; 7 Department of Cardiac-Thoracic-Vascular Sciences and Public Health, University of Padua, Italy (K.P., R.C.).; 8 Department for Cardiology, Inselspital, Bern University Hospital, University of Bern, Switzerland (A.M.D., B.A.).; 9 Cardiovascular Genetics Centre, Montreal Heart Institute, Université de Montréal, Canada (J.C.-T.).; 10 Department of Clinical Genetics, Amsterdam UMC, University of Amsterdam, the Netherlands (R.L.D., J.P.v.T.).; 11 Department of Genetics, University of North Carolina, Chapel Hill (J.M., C.T., C.L.K.).; 12 Department of Genetics, University of Utrecht, University Medical Center Utrecht, the Netherlands (J.P.v.T.).

**Keywords:** desmosomes, diagnosis, genes, genetic testing, tachycardia

## Abstract

**Background:**

Arrhythmogenic right ventricular cardiomyopathy (ARVC) is an inherited disease characterized by ventricular arrhythmias and progressive ventricular dysfunction. Genetic testing is recommended, and a pathogenic variant in an ARVC-associated gene is a major criterion for diagnosis according to the 2010 Task Force Criteria. As incorrect attribution of a gene to ARVC can contribute to misdiagnosis, we assembled an international multidisciplinary ARVC Clinical Genome Resource Gene Curation Expert Panel to reappraise all reported ARVC genes.

**Methods:**

Following a comprehensive literature search, six 2-member teams conducted blinded independent curation of reported ARVC genes using the semiquantitative Clinical Genome Resource framework.

**Results:**

Of 26 reported ARVC genes, only 6 (*PKP2*, *DSP*, *DSG2*, *DSC2*, *JUP*, and *TMEM43*) had strong evidence and were classified as definitive for ARVC causation. There was moderate evidence for 2 genes, *DES* and *PLN*. The remaining 18 genes had limited or no evidence. *RYR2* was refuted as an ARVC gene since clinical data and model systems exhibited a catecholaminergic polymorphic ventricular tachycardia phenotype. In ClinVar, only 5 pathogenic/likely pathogenic variants (1.1%) in limited evidence genes had been reported in ARVC cases in contrast to 450 desmosome gene variants (97.4%).

**Conclusions:**

Using the Clinical Genome Resource approach to gene-disease curation, only 8 genes (*PKP2*, *DSP*, *DSG2*, *DSC2*, *JUP*, *TMEM43*, *PLN*, and *DES*) had definitive or moderate evidence for ARVC, and these genes accounted for nearly all pathogenic/likely pathogenic ARVC variants in ClinVar. Therefore, only pathogenic/likely pathogenic variants in these 8 genes should yield a major criterion for ARVC diagnosis. Pathogenic/likely pathogenic variants identified in other genes in a patient should prompt further phenotyping as variants in many of these genes are associated with other cardiovascular conditions.

## Abstract 2

Supplemental Digital Content is available in the text.

Arrhythmogenic right ventricular cardiomyopathy (ARVC) is an inherited cardiomyopathy characterized by fibro-fatty myocardial replacement, frequent ventricular arrhythmias, and slowly progressive ventricular dysfunction. Patients typically present between their second and fifth decades with symptoms associated with ventricular arrhythmias.^1^ Sudden cardiac death is a common presentation, occurring in up to half of probands.

ARVC clusters in families and its pattern of inheritance is generally autosomal dominant with age-related, reduced penetrance.^2^ The discovery that Naxos disease, a rare cardiocutaneous autosomal recessive form of ARVC, was caused by pathogenic variants in *JUP*-encoded plakoglobin^3^ prompted rapid identification of pathogenic variants in other desmosomal genes (*PKP2*, *DSP*, *DSG2*, and *DSC2*) in ARVC populations. In contemporary ARVC cohorts meeting 2010 Task Force Criteria (TFC), up to two-thirds of cases have pathogenic/likely pathogenic (P/LP) desmosomal variants.^4,5^

Exponential growth in sequencing capacity led investigators to sequence ever-growing lists of candidate genes and to undertake exome and genome sequencing in attempts to identify the genetic basis of ARVC in gene-elusive patients. These studies identified variants of interest in numerous genes which were then suggested to be ARVC causative. However, some reports were based on an incomplete understanding of the extent of rare variation in the human genome. Older studies used dated diagnostic criteria for inclusion. Finally, isolated gene-elusive ARVC may be oligogenic,^2^ calling the assumptions underlying some gene identification studies into question. Despite these limitations, newly reported ARVC genes have been promptly added to diagnostic ARVC sequencing panels which now generally range from 11 to 46 genes. Genetic testing is recommended for patients with ARVC to confirm the diagnosis, inform management, and enable cascade genetic testing.^6^

The NIH-funded Clinical Genome Resource (ClinGen) created a standardized evidence-based framework to systematically assess gene-disease relationships.^7^ Recently, evaluation of genes associated with hypertrophic cardiomyopathy,^8^ long QT syndrome,^9^ and Brugada syndrome^10^ called into question causality of many disease genes. Similar weaknesses in conventional understanding of the genetic architecture for ARVC seemed likely. ARVC can be difficult to diagnose, particularly when phenotypic expression is mild or when the patient has biventricular disease raising the possibility that gene:diseases associations may have been erroneously derived from participants with other cardiovascular diseases. The TFC was updated in 2010 and older articles relied on less sensitive and specific 1994 criteria^5^ making phenotyping in older publications potentially problematic.

Since identification of a P/LP variant constitutes a major criterion in the TFC^5^ accurate understanding of genetic architecture has direct implications for ARVC diagnosis and management. Incorrect ARVC gene:disease associations may result in (1) a patient’s phenotype incorrectly attributed to a variant leading to potential over-diagnosis and incorrect cascade genetic testing or (2) a variant associated with a different disease incorrectly attributed to ARVC missing the opportunity for correct genotype-specific management of a family. Therefore, an international multidisciplinary ARVC ClinGen Gene Curation Expert Panel (GCEP; Table I in the Data Supplement; https://clinicalgenome.org/affiliation/40003/) with expertise in ARVC research, genetics, and clinical care was assembled to formally reappraise all previously reported ARVC genes using the ClinGen Gene-Disease Clinical Validity Framework. To enhance scientific rigor, we used a dual, blinded, independent curation approach (Figure 1). In this effort, we defined ARVC by fulfillment of the 2010 TFC.^5^ While arrhythmogenic cardiomyopathy has been suggested as a concept by several groups of authors,^6,11,12^ at present, there is considerable variability in the breadth of phenotypes covered by the term arrhythmogenic cardiomyopathy and no standard agreed-upon diagnostic criteria that could be applied for gene curation. Here, we report our results.

**Figure 1.**
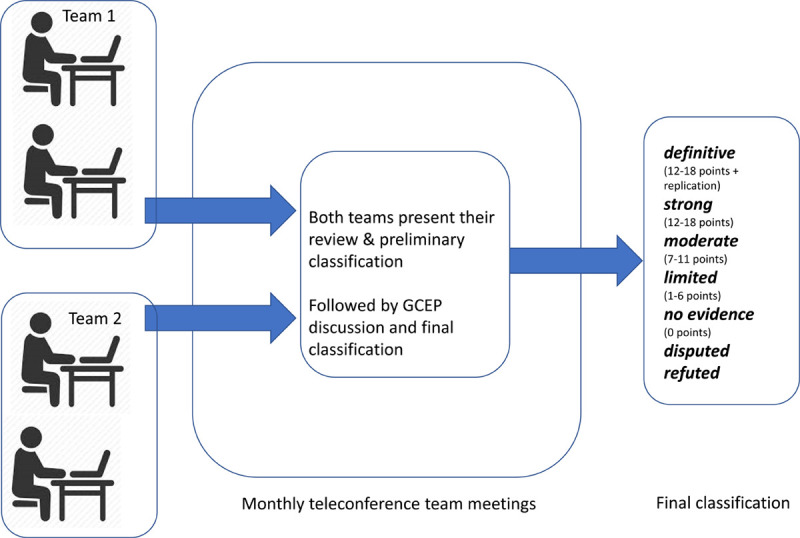
**Arrhythmogenic right ventricular cardiomyopathy (ARVC) gene curation approach.** Two-member teams conducted blinded independent dual curation using the semiquantitative Clinical Genome Resource (ClinGen) framework with ARVC-specific rules for required minor allele frequency of variants detected in patients and phenotypic evaluation of model systems. Each summarized their analysis in separate presentations for the entire ARVC gene curation expert panel (GCEP) who arrived by consensus at the final gene classification.

## Materials and Methods

The data that support the findings of this study are available from the corresponding authors upon reasonable request. The study was approved by the Johns Hopkins Medicine institutional review board (data for variant frequency cutoffs) and subjects gave informed consent. The full methods for this study are available as Data Supplement (Methods in the Data Supplement).

## Results

### Overview

**Figure 2.**
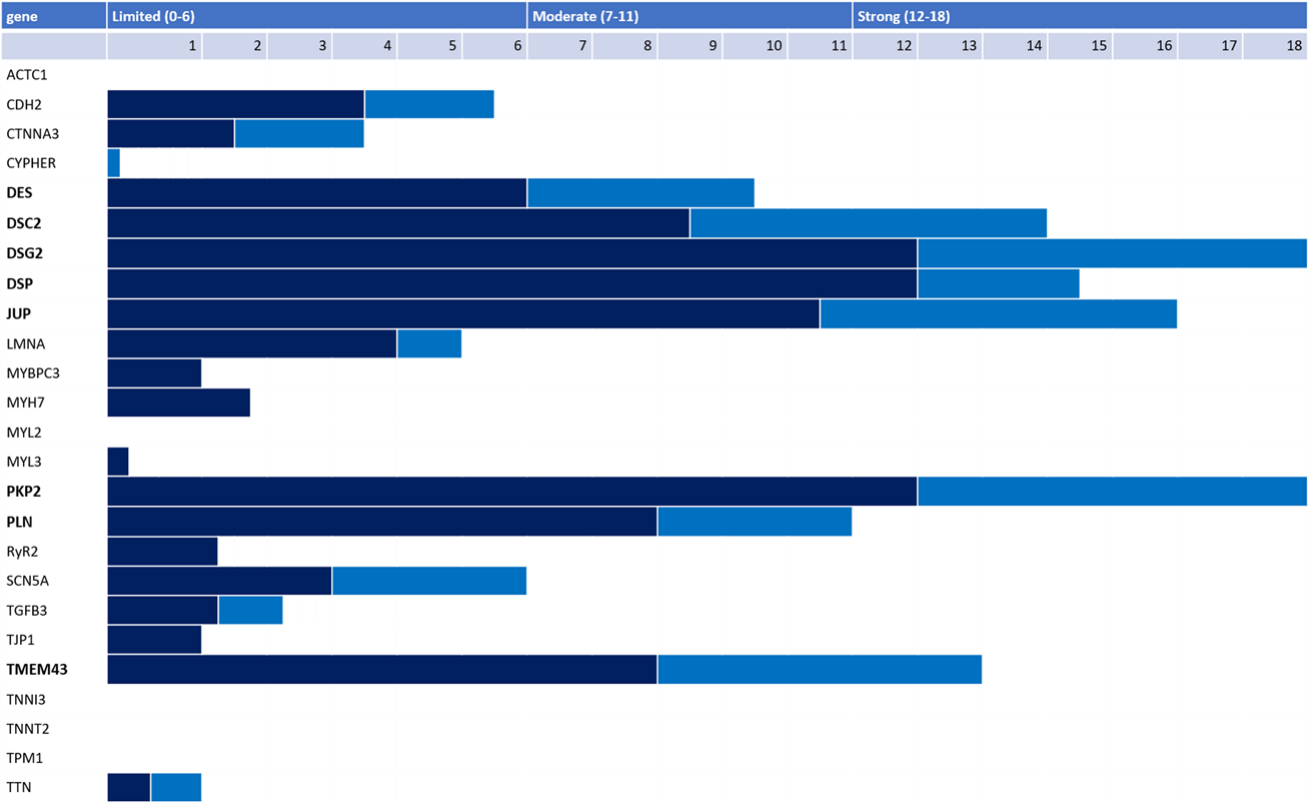
**Level of evidence scores for genes reported for arrhythmogenic right ventricular cardiomyopathy (ARVC).** Final genetic (dark blue) and experimental (light blue) evidence scores for 26 genes reported in the literature as associated with ARVC. Only 8 genes (bold font) had strong or moderate evidence for ARVC causality. The granular scores for each gene along with a complete list of references used are available in Table IV in the Data Supplement.

PubMed/OMIM searches resulted in 26 genes reported to cause human ARVC: *ACTC1*, *CDH2, CTNNA3, LDB3, DES, DSC2, DSG2, DSP, JUP, LMNA, MYBPC3, MYH7, MYL2, MYL3, PKP2, PLN, RYR2, SCN5A, TGFB3, TJP1, TMEM43, TNNI3, TNNC1, TNNT2, TPM1*, and *TTN* (Table 1). As shown in Figure 2, based on initial scoring, 6 genes (*PKP2*, *DSP*, *DSC2*, *DSG2*, *JUP*, and *TMEM43*) had strong evidence (12–18 points) and were judged to be definitive for ARVC causation as each had replication across ARVC cohorts. There was moderate evidence for 2 genes, *DES* (9.5 points) and *PLN* (11 points). The remaining genes had only limited or no evidence for ARVC causation (0–6 points). Curation team scores were highly concordant. For every gene both curation teams arrived at the same preliminary classification based on points achieved (Table II in the Data Supplement).

**Table 1.**
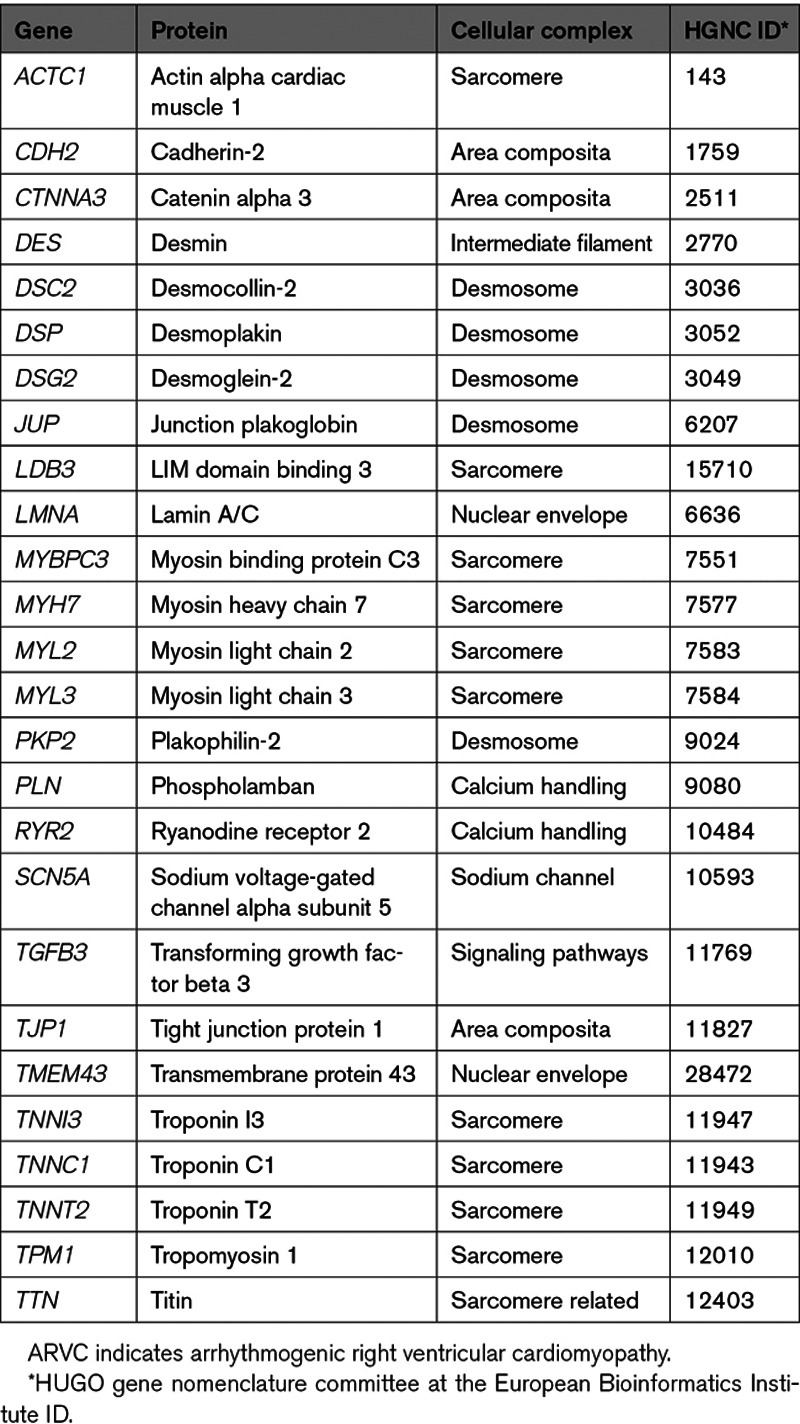
Reported Genes for ARVC

Table 2 summarizes the evidence for genes designated to have definitive or moderate evidence for ARVC causation by the GCEP. The evidence used to arrive at these final classifications was predominantly derived from clinical genetic studies. Table III in the Data Supplement shows scoring for genes with limited or no evidence for ARVC. Granular scores for each subcategory of genetic and experimental evidence can be found for each gene in Table IV in the Data Supplement. The most up-to-date curation data for each gene can be accessed at https://clinicalgenome.org/.

**Table 2.**
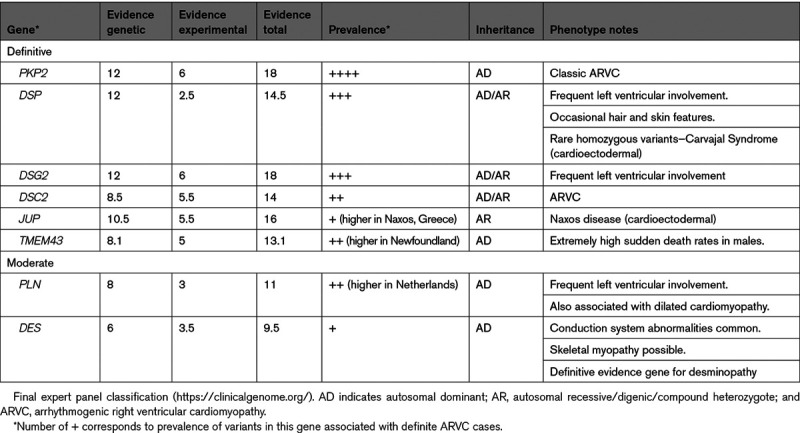
Genetic Architecture of ARVC

As shown in Table 2, the GCEP classified each gene with a score in the strong range (12–18 points) as having definitive evidence for ARVC. The genes encoding the cardiac desmosome (*PKP2*, *DSP*, *DSG2*, *DSC2*, and *JUP*) have been consistently identified across multiple ARVC cohorts. *TMEM43* was initially identified due to a founder variant segregating in a well-characterized ARVC population in Newfoundland, and subsequently in the United Kingdom, Denmark, and Germany and recently on a different haplotype in Spain.^13–15^ The GCEP confirmed the moderate evidence classification of *PLN* which was likewise initially associated with ARVC due to a segregating founder variant.^16^ A moderate evidence classification was also confirmed for *DES*. While not typically considered an ARVC gene, it is frequently associated with a myopathy^17^ in which arrhythmogenic cardiomyopathy (myofibrillar myopathy-1, desminopathy) is a presenting feature and patients can meet TFC.^18^

The GCEP concluded the remaining 18 genes—more than two-thirds of the genes analyzed—did not have convincing evidence for ARVC causality. These fell into 2 categories: (1) genes—often newly published—with rare variants detected in small families with a clear ARVC phenotype but for which data was yet limited and (2) genes known to be associated with other cardiomyopathies or arrhythmia syndromes where evidence for ARVC causality was relatively scant. Notably, the panel refuted *RYR2* as an ARVC gene, finding P/LP variants in *RYR2* typically cause catecholaminergic polymorphic ventricular tachycardia (CPVT) not ARVC. All the literature asserting ARVC causality was judged to be incorrect and based on incomplete phenotyping, wrong interpretation of clinical data (personal communication G. Thiene and C. Basso), use of dated diagnostic criteria, and designation of relatively common *RYR2* variants as pathogenic.

### Genes With Definitive Evidence for ARVC Causality

Cardiac desmosomes are specialized structures composed of proteins (cadherins, armadillo proteins, and plakins) responsible for cardiomyocyte adhesion. ARVC is classically considered a disease of the desmosome and the desmosomal genes rapidly achieved sufficient evidence for a strong designation with replication across cohorts making the gene:disease relationship definitive. There were several nuances. First, ARVC can be accompanied by skin and hair features. This has long been recognized in families with *JUP*-associated Naxos disease and *DSP*-associated Carvajal syndrome, both of which have autosomal recessive inheritance. Several authors have reported skin and hair findings in patients with heterozygous *DSP* variants which can be subtle.^19^ Importantly, there is evidence across studies for a relatively high prevalence of patients with multiple desmosomal P/LP variants beyond Naxos disease and Carvajal syndrome. Pedigrees segregating multiple *DSC2* and *DSG2* variants have been reported and inheritance more consistent with autosomal recessive than autosomal dominant inheritance has been recognized in specific populations.^20,21^

The *TMEM43* (transmembrane protein 43) gene encodes a nuclear membrane protein. One heterozygous pathogenic variant (NM_024334.3(TMEM43):c.1073C>T; p.Ser358Leu) was identified as a founder mutation in a large number of patients and families from Newfoundland, Denmark, and Germany and has also been identified in other populations.^15,22^ It is associated with a highly penetrant and arrhythmogenic subtype of ARVC in which biventricular involvement can often be appreciated. Evidence of pathogenicity of other *TMEM43* variants remains limited.

### Moderate Evidence Genes

*DES* and *PLN* had moderate evidence for ARVC causality. *DES* was initially proposed as an ARVC gene based on data from 27 Dutch individuals in 5 families segregating a rare missense variant (NM_001927.4(DES):c.38C>T; p.Ser13Phe).^23^ Cases had right ventricular involvement consistent with ARVC but also conduction disease which is atypical for ARVC. Additional families carrying *DES* LP/P variants with a clinical ARVC diagnosis as well as families with left-predominant disease have been described.^18,24,25^ Experimental evidence including expression systems integrating variants found in these families showed phenotypic alterations consistent with histological examinations of skeletal and cardiac muscle of ARVC cases^18^ and disruption of cellular adhesion.^26^ Nonetheless, *DES* variants associated with ARVC appear to be very rare and have not been observed in some large ARVC cohorts.

The *PLN* p.Arg14del variant (NM_002667.5(PLN):c.37_39AGA[1]) was first identified in ARVC in a cohort of 12/97 patients fulfilling TFC.^27^ Histology showed the typical fibro-fatty replacement and interstitial fibrosis yet compared with desmosomal gene-positive patients, *PLN* p.Arg14del showed significantly more severe fibrotic changes in the left ventricle, underscoring its biventricular character.^28^

### Limited/No Human Evidence Genes

Ten genes had limited evidence for ARVC causality (1–6 points): *SCN5A*, *LMNA*, *CDH2*, *CTNNA3*, *TGFB3*, *TTN*, *TJP1*, *MYH7*, *MYBPC3*, and *MYL3*. In comparison to the wealth of literature linking desmosomal genes with ARVC, evidence for these gene:disease relationships had been generated by relatively few research groups. For some of these genes, assertions of ARVC causality are relatively new and further data from larger cohorts might lead to an upgraded level of evidence in the future. For others, the assertion of ARVC causality was published some time ago, leading the panel to give additional weight to the failure to confirm the observation across ARVC cohorts. Our review strongly suggested that none of these genes account for a substantial fraction of patients with ARVC.

### Genes With Variants Identified in Classic ARVC Families but Yet Limited Evidence

*CDH2*, *CTNNA3*, and *TJP1* showed evidence for a classic ARVC phenotype and segregation in several relatively small families but with human data as yet limited. *CDH2* and *CTNNA3* encode the area composita proteins cadherin-2 and α T catenin, respectively. *TJP1* encodes a scaffolding protein, tight junction protein 1, which localizes to the intercalated discs in cardiomyocytes.

Evidence for *CDH2* included identification of 2 LP rare missense variants in 3 probands meeting TFC.^29,30^ One variant (NM_001792.4(CDH2): c.1219G>A; p.Asp407Asn) was identified in both a South African and a Norwegian family. Another (NM_001792.4(CDH2): c.686A>C; p.Gln229Pro) segregated among 5 affected family members. A murine knockout model showed disrupted desmosomes, cardiomyopathy, ventricular tachycardia, and sudden death, suggestive of a phenotype compatible with ARVC.

Similarly, for *CTNNA3*, 2 variants were reported in 2 ARVC probands: one likely de novo missense variant absent from gnomAD (NM_013266.3(CTNNA3): c.281T>A; p.Val94Asp) and one in-frame deletion (NM_013266.3(CTNNA3): c.2296_2298del; p.Leu766del) with limited segregation.^31^ A germline knockout mouse showed altered PKP2 distribution without affecting other junctional components of the area composita. These mice had progressive dilated cardiomyopathy (DCM), and the GCEP judged the phenotype not completely convincing for ARVC. Furthermore, no *CTNNA3* LP/P variants were reported in 2 series of gene-elusive ARVC patients.

Finally, 2 probands with ARVC (as well as 2 probands with DCM) had variants in *TJP1.*^32^ Modest segregation data allowed this to be counted as limited human evidence.

### Genes Associated With Other Cardiomyopathies/Arrhythmia Syndromes

The remaining genes curated were each strongly associated with other cardiomyopathies or arrhythmia syndromes. The GCEP concluded each had limited or no evidence for ARVC causality. The gene with the most evidence (6 points) was *SCN5A* which encodes the Nav 1.5 sodium channel and previously curated as definitive for both Brugada syndrome and long QT syndrome.^9,10^ The most robust evidence for *SCN5A* as an ARVC gene comes from identification of a variant (NM_198056.2(SCN5A): c.5693G>A; p.Arg1898His) in a gene-elusive ARVC patient via exome sequencing followed by derivation of an induced pluripotent stem cell-derived cardiomyocyte model.^33^ This model showed a one-third reduction in peak sodium current and reduced abundance of both SCN5A and CDH2 clusters at the intercalated disk which normalized in a CRISPR/Cas9 corrected line. The authors subsequently identified 5 *SCN5A* variants among 281 ARVC probands. One variant was excluded for being too common and 2 were found in probands who also had pathogenic desmosomal variants. One proband had an in-frame deletion (NM_198056.2(SCN5A): c.2184-2186del; p.Leu729del) which segregated with the phenotype, but most family members did not fulfill definite TFC.

Potentially pathogenic variants in *LMNA* have been published in several ARVC cohorts.^34,35^ While evidence was sufficient to merit a limited evidence classification, the GCEP noted that while most probands did meet TFC, many of their affected family members did not. The phenotypes observed overlapped with DCM and were characterized by prominent conduction system abnormalities and atrial arrhythmias.

Eleven articles were reviewed to assess the relationship of *TGFB3* with ARVC, the majority from the same research group. *TGFB3* emerged as a candidate gene based on linkage to 14q23-q24 in several Italian families. Sequencing found variants in the 3′ untranslated region in one family and a second regulatory noncoding variant in an unrelated family.^36^ These variants were both associated with increased activity compared with wild type in an expression assay. However, 2 of the initial families with significant linkage to the candidate region had no P/LP variants in the *TGFB3* coding sequences, UTRs, and promoter regions. No definitively pathogenic variants have been subsequently reported in ARVC probands. Nowadays, *TGFB3* is believed to underlie Loeys-Dietz syndrome type 5, a connective tissue disease phenotype with features of Marfan syndrome, including aortic abnormalities.^37^ The GCEP concluded that while *TGFB3* merited a limited evidence classification for ARVC, a variant detected in *TGFB3* should be treated with great caution and is unlikely the cause of a patient’s ARVC.

*TTN*, encoding titin, is a frequent cause of familial DCM. Nine papers were evaluated for the role of *TTN* variants in ARVC causation. Many reported missense variants which were relatively common in gnomAD. Furthermore, in study reporting 11/35 ARVC probands with *TTN* missense variants, relatives carrying the variant had no evidence of disease leading them to conclude these *TTN* variants had very low penetrance or negligible pathogenicity.^38^ One article did describe a rare missense variant NM_133378.4 (TTN): c.8678C>T; p.Thr2896Ile that segregated among 9 family members—6 of whom met TFC.^39^ An in vitro functional assay by 2 independent groups found that the variant introduced aberrant function. Further evidence has shown that *TTN*-associated DCM is not particularly associated with an arrhythmogenic phenotype.^40^ The GCEP thus concluded there was very limited evidence for *TTN* as ARVC causative.

The sarcomere genes have been considered by several research groups as a potential cause of ARVC.^41^ *MYH7*, *MYBPC3*, and *MYL3* were scored as having very limited evidence. The other sarcomere genes had no evidence. These genes are well-established as causative for hypertrophic cardiomyopathy.^8^

### Refuted and Disputed Genes—*RYR2* Is Not an ARVC Gene

The GCEP refuted the association of *RYR2* with ARVC. A thorough review, including 57 articles, showed that the assertion of ARVC causality was initially derived from 3 publications from the same research group who first established linkage to chromosome 1q42-43, and subsequently to *RYR2* in families with a phenotype called arrhythmogenic right ventricular dysplasia 2 described as CPVT with fibro-fatty replacement of the right ventricle.^42^ The clinical features described in these manuscripts reflect CPVT rather than ARVC with cases not meeting TFC. This was confirmed by collaborators from the original research group (C. Basso and G. Thiene personal communication). A mouse model with one of the variants also showed a CPVT-like phenotype with no evidence of fibro-fatty infiltration or structural alterations characteristic of ARVC.^43^ In articles reporting *RYR2* missense variants in possible ARVC probands the minor allele frequency was often too high, cases did not have a clear ARVC diagnosis, segregation information was often not informative, and in several cases, CPVT was said to also be present in the family. Rare cases of *RYR2* deletions associated with DCM with CPVT-like arrhythmias have been described,^44^ but none associated with ARVC. Taken together, the evidence is convincing that pathogenic variants in *RYR2* do not cause ARVC, rather they cause CPVT.

*LDB3* was disputed as a cause for ARVC. The only variant reported in an ARVC family had a minor allele frequency higher than the cutoff established, particularly in the relevant ethnic population (Europeans).

### Prevalence of Variants Reported in ClinVar for Each Gene

**Figure 3.**
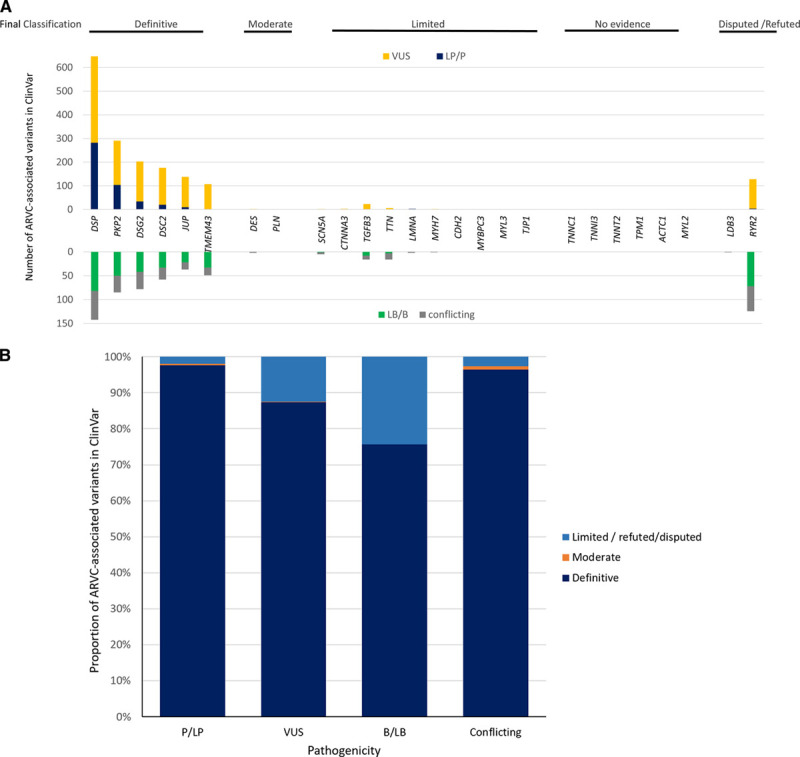
**Variants in ClinVar in arrhythmogenic right ventricular cardiomyopathy (ARVC) gene curation expert panel (GCEP) curated genes.** **A**, Distribution of variants in each gene curated—pathogenic and likely pathogenic (P/LP) variants (blue) were reported primarily in genes encoding the cardiac desmosome. **B**, Nearly all P/LP variants reported in ClinVar for ARVC are in the genes categorized as definitive or moderate evidence ARVC genes while refuted/disputed/limited evidence genes account for a higher proportion of variant of uncertain significance (VUS) and benign/likely benign (B/LB) variants.

Figure 3 shows the distribution of variants reported to ClinVar associated with ARVC (Figure 3A) and the proportion of P/LP variants found in the genes with definitive or moderate evidence for ARVC in comparison to the other genes (Figure 3B). As can be appreciated, ARVC-associated P/LP variants were nearly exclusively reported in the desmosomal genes (450/462, 97.4%) with the established founder variants in *PLN* and *TMEM43* also reported. Notably, only 5 P/LP variants (1.1%) were reported in genes with limited evidence, including one variant in *CTNNA3*, 3 in *LMNA*, and one in *TGFB3*.

## Discussion

The data presented here, derived from a rigorous, international evaluation of 26 genes published as ARVC causing using the ClinGen framework, confirm that ARVC is primarily a disease of the cardiac desmosome, with *PKP2*, *DSP*, *DSC2*, *DSG2*, and *JUP*, definitively associated with ARVC and these genes accounting for nearly all reported ARVC-associated P/LP variants. *PLN* and *TMEM43* contribute to disease pathogenesis, particularly in geographic regions with well-characterized founder variants. This study also demonstrates that the majority of published ARVC genes had only limited (N=10) or no (N=8) evidence and contribute little to the classic ARVC phenotype. While there has been extensive discussion of the genetic heterogeneity of ARVC and overlap syndromes, P/LP variants in genes with strong/definitive evidence for another cardiovascular disease do not substantially contribute to ARVC causation. In particular, this analysis disqualified *RYR2* as an ARVC gene, finding cases and model systems instead had CPVT. Taken together, these findings call into question the extent of genetic heterogeneity truly contributing to classic ARVC as defined by the TFC.

This reappraisal of ARVC genes is strengthened by our methodological approach that included an extensive literature review, dual, blinded, independent curation, and final adjudication of evidence by an international multidisciplinary panel with substantial experience with ARVC. The semiquantitative ClinGen framework for evidence classification was effective. The independent curation teams had a high degree of uniformity in applying the framework with 100% concordance of preliminary classification of the level of evidence and no disagreement between the curation teams’ conclusions and the final opinion of the other GCEP members in arriving at the final level of evidence.

Eighteen of the 26 published ARVC genes had either limited or no evidence for ARVC causation. Excluded genes fell into 2 categories: (1) recently published genes with rare variants detected in small families with clear ARVC but for which data was limited and (2) genes known to be associated with other cardiomyopathies or arrhythmia syndromes where a thorough literature review showed limited or no evidence for ARVC causality. For the genes in the first category (*CDH2*, *CTNNA3*, and *TJP1*) multicenter studies, some currently underway, will address whether the identification of segregating P/LP variants in ARVC families will be replicated and if so in what proportion of gene-elusive ARVC cases. These data could lead to the level of evidence for these genes being upgraded in the future.

This misattribution of the latter group of genes to ARVC illustrates the well-known challenges of ascertaining, and then attributing, clinical data to a specific cardiac disease. This challenge, particularly before 2010 when the ARVC diagnostic criteria were refined, was the primary reason for the erroneous assertion that *RYR2* caused ARVC, with our review revealing affected individuals in published pedigrees segregating *RYR2* variants had clinical characteristics consistent with CPVT rather than ARVC. Diagnostic challenges also emerged in papers that described associations of *LMNA*, *TTN*, *SCN5A*, and even the moderate evidence gene *DES* with ARVC. In most, while several cases met 2010 TFC, others did not and the pedigrees often included clinical features not typically seen in ARVC.

Older genetic/genomic methodologies also contributed to incorrect assertions of ARVC causality. Some articles identified variants of interest as LP based on small control cohorts. Reassessment revealed the minor allele frequency of quite a few variants was too high given current understanding of the frequency of rare variants in the general population.

### Clinical Implications

Genetic testing is recommended for patients with ARVC and genetic test results are part of the 2010 TFC.^5,6^ Optimal genetic testing requires both wise genetic test selection and accurate interpretation of results. By defining the genetic architecture of ARVC, this study informs both.

### Interpretation and Utilization of Genetic Test Results for Diagnosis and Cascade Testing

A pathogenic variant categorized as associated or probably associated with ARVC constitutes a major ARVC diagnostic criterion.^5^ Based on our results, we recommend that only P/LP variants in genes with definitive and moderate evidence for ARVC causation (*PKP2*, *DSP*, *DSC2*, *DSG2, JUP, TMEM43, PLN*, and *DES*) should yield a major criterion for ARVC diagnosis.

We found that genes with strong or definitive evidence for other cardiovascular diseases had at-most moderate (*DES*) and usually limited or no evidence for ARVC causation. This suggests skepticism is warranted when a P/LP variant in one of these genes is identified in a putative ARVC patient. The American College of Medical Genetics and Genomics explicitly warns against relying on their guidelines for interpretation of pathogenicity in genes of unknown significance.^45^ Reevaluation of the patient and family for features suggesting an alternate clinical diagnosis may be useful and the full associated clinical spectrum (eg, desminopathy, laminopathy) should inform medical care and familial cascade screening. In the absence of evidence suggesting an alternate diagnosis, reevaluating the pathogenicity of the variant may be warranted.

A few percent of patients with ARVC have multiple P/LP variants in strong/moderate evidence ARVC genes, leading to earlier and more severe ARVC manifestations. The rare patients with definite ARVC and also definitively pathogenic variants in limited evidence genes typically associated with other cardiovascular diseases are particularly likely to harbor additional genetic variants. Real harm can be done by cascade testing of a variant which does not (fully) explain the disease in a family. These second variants in other cardiomyopathy or arrhythmia related genes may also drive the phenotype towards an atypical yet severe, manifestation of ARVC. This underscores that while ARVC is not a condition with substantial genetic heterogeneity, the paradigm of one gene-one disease is challenged.^46^ Evidence suggests oligogenic inheritance with multiple additional variants that may or may not reach the LP/P status (or in genes that are not definitive for ARVC) could also contribute to disease expression.

Furthermore, recent publications show at least one-third of ARVC cases are gene elusive,^12^ and these patients are disproportionately high-level athletes with no family history of ARVC, pointing to exercise as contributing to cause.^47^ Among relatives of patients with ARVC with P/LP desmosomal variants, exercise increases penetrance and risk of incident arrhythmias, but not all athletic relatives develop ARVC.^48^. Additionally, a recent study^49^ showed 0.23% of a general clinical population harbored a loss of function desmosomal variant. These patients had extremely low ARVC penetrance (estimated at 6%) and were no more likely than controls to have ECG or echocardiography findings that met TFC. Taken together this evidence strongly suggests a threshold model of ARVC pathogenesis in which multiple hits, both environmental and genetic, are required for disease expression.^2^ Thus, while detection of a P/LP variant in a definitive or moderate evidence gene in a patient with ARVC features is highly indicative of disease, it does not fully predict the clinical features or course of individual patients. The fact that these different aspects influence disease expression, and are not accounted for in the ClinGen framework which is built around true penetrant Mendelian disease, can be considered a limitation and are important to keep in mind when using the results of this analysis to interpret genetic tests.

In summary, substantial caution is required for the interpretation of variants in limited evidence genes which are unlikely to be the sole cause of disease in a patient with ARVC. Our results suggest such variants should not be used to assign the patient a major TFC criterion.

### Panel Selection for Genetic Testing

This study also challenges the inclusion of several genes frequently present in ARVC panels—foremost among them *RYR2*. Our results identify genes with definitive and moderate evidence for ARVC and show that most P/LP variants in patients with ARVC occur in these genes. However, a clinical ARVC diagnosis can be challenging, particularly in early stages of disease where structural abnormalities can be subtle but arrhythmic risk is nonetheless significant.^1^ Careful use of a larger panel can, therefore, facilitate correct genetic diagnosis of a family. Using a large panel responsibly in this context requires multidisciplinary expertise.^6^

### Limitations

Genes were curated for ARVC per 2010 TFC. Updated diagnostic criteria are being considered, particularly for the left-dominant form of ARVC,^50^ thus a need to update ARVC curation is anticipated.

Although genes accounting for most familial ARVC have been identified, expanded sequencing efforts and new analytic approaches will identify rare or family specific variants in novel putative ARVC genes that will require adjudication (foremost among these *FLNC*). Recuration of limited and moderate evidence genes will be done per ClinGen procedures as new data emerges (https://clinicalgenome.org/site/assets/files/2164/clingen_standard_gene-disease_validity_recuration_procedures_v1.pdf).

### Conclusions

This evidence-based reevaluation of published ARVC genes by experts in the field shows that only a small number of genes (*PKP2*, *DSP*, *DSG2*, *DSC2*, *JUP*, *TMEM43*, *PLN*, and *DES*) are definitively or moderately associated with ARVC and these genes account for the overwhelming majority of P/LP variants in patients with ARVC. We recommend only P/LP variants in these 8 genes should yield a major criterion for ARVC diagnosis by TFC. This analysis is expected to further refine the utility of genetic data in caring for families with ARVC by assisting the clinician in determining what test to order and also by quantifying the strength of evidence underlying the gene:disease relationship relevant to a genetic result.

## Sources of Funding

This work was financially supported by grants from the National Institutes of Health (NIH; U41HG009650) and by the Netherlands Cardiovascular Research Initiative (Dr van Tintelen) an initiative supported by the Dutch Heart Foundation (CVON2018-30 PREDICT2 and CVON 2015-12 eDETECT). The Johns Hopkins ARVD/C Program (Dr James and B. Murray) is supported by the Leonie-Wild Foundation, the Leyla Erkan Family Fund for ARVD Research, the Dr Satish, Rupal, and Robin Shah ARVD Fund at Johns Hopkins, the Bogle Foundation, the Healing Hearts Foundation, the Campanella family, the Patrick J. Harrison Family, the Peter French Memorial Foundation, and the Wilmerding Endowments. Dr Syrris was supported by Fondation Leducq Transatlantic Networks of Excellence Program grant no 14CVD03 and the National Institute for Health Research University College London Hospitals Biomedical Research Centre (United Kingdom). Dr Protonotarios is supported by a British Heart Foundation clinical research training fellowship grant (FS/18/82/34024).

## Disclosures

B. Murray and E. Brown are consultants for MyGeneCounsel. The other authors report no conflicts.

## Supplemental Materials

Supplemental Methods

Supplemental Tables I–IV

References ^51,52^

## Supplementary Material


