# Supplementary material for: International Evidence Based Reappraisal of Genes Associated With Arrhythmogenic Right Ventricular Cardiomyopathy Using the Clinical Genome Resource Framework
Source: Circ Genom Precis Med. 2021 Apr 8;14(3):e003273. doi: 10.1161/CIRCGEN.120.003273 (PMC8205996; doi:10.1161/CIRCGEN.120.003273)
Supplement: Supplementary file 1 [file hcg-14-e003273-s001.pdf]

SUPPLEMENTAL MATERIAL

Supplemental Methods

Supplementary Tables

Table I: Curation teams

Table II: Concordance of Team 1 and Team 2 initial curation scores

Table III: Genes with limited or no contribution to ARVC pathogenesis – Final expert panel classification

Table IV: Final ARVC gene validity curations in the gene curation interface (GCI) including granular scores, manuscripts reviewed, and evidence summaries

Supplemental Methods

Definition of ARVC

ARVC was defined by fulfillment of the 2010 Task Force Criteria[5].

Identification of genes to be curated

Genes were selected for evaluation by a PubMed and OMIM search in December 2017 to identify genes putatively causative for ARVC. This search was repeated in 2019 to identify genes newly associated with ARVC.

To identify genes for which an assertion for a role in human ARVC causation had been made we first conducted a search in the Gene resource of NCBI (https://www.ncbi.nlm.nih.gov/gene). We conducted the following search:

("arrhythmogenic right ventricular" OR "arrhythmogenic cardiomyopathy" OR "right ventricular dysplasia") AND "Homo sapiens"[porgn:__txid9606]

Genes on the resulting list were investigated via PubMed to confirm that an assertion for ARVC-causation had indeed been made. Genes in which the relevant study reported lack of association (eg. *PLEC*, *SCN10A*, *PERP*, etc) or had been potentially implicated in a downstream pathway or model system only were discarded. Additional assertions made for the sarcomere genes were discovered during this process when investigating *MYBPC3* and *MYH7* so these genes were added to the list to be curated.

We then confirmed completeness of our list by querying OMIM

In OMIM (https://www.omim.org/), the following disease entities were searched:

arrhythmogenic right ventricular cardiomyopathy, arrhythmogenic right ventricular dysplasia, arrhythmogenic right ventricular dysplasia/cardiomyopathy, arrhythmogenic right ventricular cardiomyopathy/dysplasia, arrhythmogenic cardiomyopathy, right ventricular dysplasia, and abbreviations: ARVC, ARVD, ARVD/C, ARVC/D, ACM, AC

Gene curation approach

Our gene curation approach is summarized in **Figure 1**. To enhance the rigor of our evaluation of putative ARVC genes we conducted dual independent, blinded, curation. We assembled six 2-person gene curation teams. Each curation team included members with expertise in both ARVC diagnosis and clinical care and in genetic analysis. Two curation teams independently evaluated each gene and worked blinded to each other. Curation teams were excluded from primary or secondary evaluation of a gene if either member had published an assertion regarding ARVC causality of the gene. Curation team composition, expertise, and genes curated by each team are shown in **Supplementary Table I**.

Curation teams used a standard operating procedure (SOP) for gene curation per the ClinGen Gene Curation Framework, described in detail below, to preliminarily categorize gene-disease relationships into different clinical validity levels including Strong (12-18 points), Moderate (7-11 points), Limited (1-6 points), or no reported (human) evidence (0 points) (11). In this semi-quantitative scoring system genetic and experimental evidence are reviewed separately, each to saturation, and tallied for a final score.

For each gene, curation teams independently presented their results during biweekly teleconferences of the ARVC Gene Curation Expert Panel (GCEP) who discussed the evidence to determine the final clinical validity of each gene. Final classifications included the categories listed above plus options to dispute or refute the relationship of the gene with ARVC, or to consider the

gene:disease relationship definitive. Definitive-evidence genes were those garnering 12-18 points with clear evidence of replication in the literature. Disputed genes were those in which evidence had arisen since the initial publication calling into question ARVC causality while for refuted genes the evidence against ARVC causality significantly outweighed any evidence supporting causality.

Application of the ClinGen gene curation framework

To ensure consistency in the data curated, pre-curation of each gene was performed by a PhD-level ClinGen staff biocurator and GCEP member. Details of the ClinGen precuration approach are available at: https://clinicalgenome.org/working-groups/lumping-and-splitting/. Articles of interest and PMIDs (Pubmed identifier) were circulated to each curation team. Curation teams also independently reviewed the literature and shared any additional PMIDs with the corresponding team.

ARVC gene curation followed ClinGen Gene Curation Framework standard operating procedures (SOP) (https://clinicalgenome.org/docs/summary-of-updates-to-the-clingen-gene-clinical-validity-curation-sop-version-7/). Broadly speaking, this framework provides a systematic approach to evaluating the strength of evidence underpinning gene:disease associations. In this semi-quantitative scoring system points are accrued based on published genetic and experimental evidence. Genetic evidence includes assessment of clinical phenotype, variant pathogenicity, study design, type and extent of sequencing performed, and evidence of familial segregation of variants. Experimental evidence includes protein expression, in vitro assays assessing functional implications of disease-associated variants, and data from model organism and rescue studies. For both genetic and experimental evidence publications were scored until the curation team reached saturation (maximum 12 points of genetic evidence and 6 points of experimental evidence). All team members received training in the application of this framework.

Furthermore, several disease-specific guidelines for scoring evidence were required that are summarized below.

Minor allele frequency cutoff

To curate human genetic evidence, it was necessary to define a minor allele frequency (MAF) cut-off for considering a variant potentially causative. We used two approaches to defining this value. Our first approach queried evidence from ARVC cohorts. We reasoned that the MAF of any variant of interest should be less common than the most frequent variants identified in the ARVC population. We reviewed the 15 most frequent variants detected in a cohort of ARVC probands from a combined Dutch, US, and German registry[51]. These were all *PKP2* variants, primarily premature terminating and splice variants. The variant with the highest allele frequency in gnomAD was c.2146-1G>C with an MAF of 0.000036. The second approach utilized a recently developed allele frequency calculator that integrates inheritance, allelic and genetic heterogeneity and penetrance[52]. This calculator generated an MAF of 0.00004, entirely consistent with the population-generated value. Only variants with a MAF < 0.000036 were scored during curation.

Phenotype in animal models

Experimental evidence scores were assigned per the ClinGen framework. A cardiomyopathic phenotype preferably with fibrofatty replacement of cardiomyocytes was considered sufficient evidence in an animal model, however, models showing ventricular arrhythmias alone were not scored.

<u>Gene curation and classification process</u>

As shown in **Figure 1**, each curation team entered data directly into an electronic gene curation interface (GCI) that records and tracks data entry. Team 1 used the GCI of record. A copy GCI (demo version) was built to allow Team 2 data to be recorded for research purposes. Preliminary clinical

validity classifications generated by each team using the automated GCI scoring included: Strong (12-18 points), Moderate (7-11 points), Limited (1-6 points), or no reported (human) evidence (0 points).

For each gene, both teams independently presented their curation results via power point presentations during biweekly teleconferences of the ARVC GCEP. The GCEP discussed the presented evidence to arrive at consensus on the final clinical validity of each gene. Final classifications included the categories listed above plus options to dispute or refute the relationship of the gene with ARVC, or to consider the gene:disease relationship definitive. Definitive-evidence genes were those garnering 12-18 points with extensive replication in the literature. Disputed genes were those in which evidence had arisen calling into question ARVC causality. Per the ClinGen framework, this evidence need not outweigh the evidence for the gene:disease relationship. In contrast, refuted genes were those in which the evidence against ARVC causality significantly outweighed any evidence supporting causality. This designation was used when the GCEP considered it had been proven to the extent possible that there is no valid evidence for ARVC causality of a gene.

Several genes (*PKP2*, *DSG2*, *DSC2, TMEM43*) had been previously curated for ARVC as part of the ClinGen evaluation of genes recommended by the American College of Medical Genetics and Genomics (ACMG) for return when detected as secondary findings. For these genes, an updated literature review was performed and the evidence was presented by a ClinGen curator. The evidence was then reviewed and the GCEP decided on a final classification.

<u>Unique variants in ClinVar per ARVC gene</u>

We analyzed ClinVar submissions (0-3 stars) for ARVC for all 26 genes to assess the relative frequency of variants adjudicated as P/LP for each gene and compared this frequency among different final curation classifications. ClinVar was queried on March 4, 2020.

Supplementary Table I: Curation teams

Curation teams	Curators	Affiliation	Genes curated
**Team 1**	Brittney Murray MS, CGC<br>Cynthia A. James PhD, CGC	Division of Cardiology, Dept. of Medicine, Johns Hopkins University, Baltimore, Maryland, USA	*TJP1*, *LMNA*, *CDH2*, *TNNI3*, *TNNT2*, *DSP*
**Team 2**	Ronald Lekanne Deprez PhD	Dept. of Clinical Genetics, Amsterdam UMC, University of Amsterdam, Amsterdam, The Netherlands	*RYR2*, *CTNNA3*, *TTN*, *TGFB3*, *CDH2*
	J. Peter van Tintelen MD, PhD	Department of Genetics, University of Utrecht, University Medical Center Utrecht, Utrecht, the Netherlands	
**Team 3**	Alexandros Protonotarios MD<br>Petros Syrris PhD	Centre for Heart Muscle Disease, Institute of Cardiovascular Science, University College London, London, UK	*DES*, *ACTC1*, *MYH7*, *MYL2*, *LDB3*, *PLN*, *MYBPC3*
**Team 4**	Babken Asatryan MD, PhD<br>Argelia Medeiros Domingo MD, PhD	Dept. for Cardiology, Inselspital, Bern University Hospital, University of Bern, Bern, Switzerland	*DSP*, *DES*, *LMNA*, *TTN,* *TNNI3*, *TNNT2*, *ACTC1*, *MYL3*, *MYL2*, *SCN5A*
**Team 5**	Julia Cadrin-Tourigny MD	Cardiovascular Genetics Center, Montreal Heart Institute, Université de Montréal, Montréal, Canada	*JUP*, *PLN*, *RYR2*, *TGFB3*, *TJP1*, *TPM1*, *TNNC1*
	Daniel P. Judge MD	Division of Cardiology, Department of Medicine Medical University of South Carolina, Charleston, South Carolina, USA	
**Team 6**	Rudy Celeghin PhD<br>Kalliopi Pilichou PhD	University of Padua, Department of Cardiac-Thoracic-Vascular Sciences and Public Health, Padua, Italy	*LDB3*, *SCN5A*, *MYL3*, *TPM1*, *TNNC1*, *JUP*, *CTNNA3*, *MYBPC3*, *MYH7*

Supplementary Table II: Concordance of Team 1 and Team 2 initial curation scores

Gene	Team 1 score	Team 1 classification	Team 2 score	Team 2 classification	Concordant preliminary classification?
ACTC1	0	no reported evidence	0	no reported evidence	YES
CDH2	5.5	limited	6.0	limited	YES
CTNNA3	3.5	limited	3.0	limited	YES
DES	9.5	moderate	10.5	moderate	YES
DSC2	14	definitive	Prior ClinGen adjudication	definitive	YES – concordant with ClinGen review of ACMG59
DSP	14.5	definitive	15.5	definitive	YES
DSG2	18.0	definitive	Prior ClinGen adjudication	definitive	YES – concordant with ClinGen review of ACMG59
JUP	16.0	strong	15.5	strong	YES
LDB3	0.1	limited	0.1	limited	YES
LMNA	5.0	limited	6.0	limited	YES
MYBPC3	1.0	limited	0.75	limited	YES
MYH7	1.75	limited	1.75	limited	YES
MYL2	0	no reported evidence	0	no reported evidence	YES
MYL3	0.25	limited	0.25	limited	YES
PKP2	18.0	definitive	Prior ClinGen adjudication	definitive	YES – concordant with ClinGen review of ACMG59
PLN	11.0	moderate	11.0	moderate	YES
RYR2	1.0	refuted	1.25	refuted	YES
SCN5A	6.0	limited	2.75	limited	YES
TGFB3	2.2	limited	1.5	limited	YES
TJP1	1.0	limited	1.0	limited	YES
TMEM43	13.1	definitive	11.6 (prior ClinGen review)	strong	YES – additional points from recent publications not initially reviewed.
TNNI3	0	no reported evidence	0	no reported evidence	YES
TNNC1	0	no reported evidence	0	no reported evidence	YES
TNNT2	0	no reported evidence	0	no reported evidence	YES
TPM1	0	no reported evidence	0	no reported evidence	YES
TTN	1.0	limited	1.5	limited	YES

Supplementary Table III: Genes with limited or no contribution to ARVC pathogenesis – Final expert panel classification

	**Evidence** Genetic	**Evidence** experimental	**Evidence total**	**Inheritance**	**Phenotype notes**
**Limited**					
SCN5A	3	3	6	AD	ARVC. Typically associated with Brugada syndrome, dilated cardiomyopathy, long QT syndrome
LMNA	4	1	5	AD	Typically associated with dilated cardiomyopathy with conduction disease
CDH2	3.5	2	5.5	AD	ARVC – few cases reported
CTNNA3	1.5	2	3.5	AD	ARVC – few cases reported
TGFB3	1.2	1	2.2	AD	ARVC – few cases reported, Loeys Dietz gene
TTN	0.5	0.5	1	AD	Typically associated with dilated cardiomyopathy
TJP1	1	0	1	AD	ARVC and dilated cardiomyopathy – few cases reported
MYH7	1.75	0	1.75	AD	Definitive evidence HCM gene
MYBPC3	1	0	1	AD	Definitive evidence HCM gene
MYL3	0.25	0	0.25	AD	Definitive evidence HCM gene
**Refuted**					
RYR2	1.25	0	1.25	AD	Of 55 papers reviewed, cases overwhelmingly had a CPVT phenotype
**Disputed**					
LDB3	0	0.1	0.1	AD	ARVC phenotype, but MAF too high
**No human evidence**					
TNNI3	0	0	0	AD	Definitive evidence HCM gene
TNNT2	0	0	0	AD	Definitive evidence HCM gene
ACTC1	0	0	0	AD	Definitive evidence HCM gene
MYL2	0	0	0	AD	Definitive evidence HCM gene
TMP1	0	0	0	AD	Definitive evidence HCM gene
TNNC1	0	0	0	AD	Moderate evidence HCM gene

AD: Autosomal dominant

The most up-to-date curation data for each gene can be accessed at https://clinicalgenome.org/

Gene Validity Curations

ACTC1 - arrhythmogenic right ventricular cardiomyopathy

Gene:	ACTC1 (HGNC:143)
Disease:	arrhythmogenic right ventricular cardiomyopathy (MONDO_0016587)
Mode of Inheritance:	Autosomal dominant inheritance (HP:0000006)

No Known Disease Relationship

Classification - 03/15/2019

Replication over time: NO　　Contradictory Evidence: NO

Expert Panel: Arrhythmogenic Right Ventricular Cardiomyopathy EP

Evidence Summary: ACTC1: Arrhythmogenic Right Ventricular Cardiomyopathy (ARVC) HGNC: 143 MONDO_0016587 Mode of Inheritance: Autosomal dominant inheritance (HP:0000006) Expert Panel: Arrhythmogenic Right Ventricular Cardiomyopathy SOP: Gene Clinical Validity Standard Operating Procedures (SOP), Version 6 Calculated Classification (date) No reported evidence 22/02/2019 Evidence Summary: There is no evidence that ACTC1 is involved in ARVC. This gene has been investigated for pathogenic variants in three populations of patients meeting 2010 Task Force criteria for ARVC: a cohort of 14 Spanish unrelated patients (22421524), a cohort of 14 cases (27194543) and a cohort of 137 ARVC patients (29709087). No variants in ACTC1 were reported.
Gene Clinical Validity Standard Operating Procedures (SOP) - Version 7

			Guidelines			Points			
	Evidence Type	Case Information Type	Default	Range	Max	Count	Total	Counted	PMIDs/Notes
Genetic Evidence / Case-Level Data / Variant Evidence	Autosomal Dominant or X-linked Disorder	Variant is de novo	2	0-3	12				
		Proband with predicted or proven null variant	1.5	0-2	10				
		Proband with other variant type with some evidence of gene impact	0.5	0-1.5	7				
	Autosomal Recessive Disease	Two variants in trans and at least one de novo or a predicted/proven null variant	2	0-3	12				
		Two variants (not predicted/proven null) with some evidence of gene impact in trans	1	0-1.5					
Segregation Evidence				Summed LOD		Family Count			
		Candidate gene sequencing							
		Exome/genome or all genes sequenced in linkage region							
		Total Summed LOD Score							

	Case-Control Study Type	Case-Control Quality Criteria	Guidelines Points/Study	Max	Count	Points	Points Counted	PMIDs/Notes
Case-Control Data	Single Variant Analysis	1. Variant Detection Methodology<br>2. Power<br>3. Bias and confounding<br>4. Statistical Significance	0-6	12				
	Aggregate Variant Analysis		0-6					
	**Total Genetic Evidence Points (Maximum 12)**						0	

Evidence Type		Guidelines Default	Range	Max	Points Count	Total	Counted	PMIDs/Notes
Function	Biochemical Function	0.5	0 - 2	2				
	Protein Interaction	0.5	0 - 2					
	Expression	0.5	0 - 2					
Functional Alteration	Patient cells	1	0 - 2	2				
	Non-patient cells	0.5	0 - 1					

Experimental Evidence								
Models	Non-human model organism	2	0 - 4	4				
	Cell culture model	1	0 - 2					
Rescue	Rescue in human	2	0 - 4					
	Rescue in non-human model organism	2	0 - 4					
	Rescue in cell culture model	1	0 - 2					
	Rescue in patient cells	1	0 - 2					
	**Total Experimental Evidence Points (Maximum 6)**						0	

**Assertion criteria**	**Genetic Evidence (0-12 points)**	**Experimental Evidence (0-6 points)**	**Total Points (0-18)**	**Replication Over Time (Y/N)**
**Description**	Case-level, family segregation, or case-control data that support the gene-disease association	Gene-level experimental evidence that support the gene-disease association	Sum of Genetic & Experimental Evidence	> 2 pubs w/ convincing evidence over time (>3 yrs)
**Assigned Points**	**0**	**0**	**0**	**NO**
**CALCULATED CLASSIFICATION**		**LIMITED**	**1-6**	
		**MODERATE**	**7-11**	
		**STRONG**	**12-18**	
		**DEFINITIVE**	**12-18 AND replication over time**	
**Valid contradictory evidence (Y/N)***	NO			

CALCULATED CLASSIFICATION (DATE) **No Known Disease Relationship** **05/08/2020**

EXPERT CURATION (DATE) **No Known Disease Relationship** **03/15/2019**

EVIDENCE SUMMARY

ACTC1: Arrhythmogenic Right Ventricular Cardiomyopathy (ARVC) HGNC: 143 MONDO_0016587 Mode of Inheritance: Autosomal dominant inheritance (HP:0000006) Expert Panel: Arrhythmogenic Right Ventricular Cardiomyopathy SOP: Gene Clinical Validity Standard Operating Procedures (SOP), Version 6 Calculated Classification (date) No reported evidence 22/02/2019 Evidence Summary: There is no evidence that ACTC1 is involved in ARVC. This gene has been investigated for pathogenic variants in three populations of patients meeting 2010 Task Force criteria for ARVC: a cohort of 14 Spanish unrelated patients (22421524), a cohort of 14 cases (27194543) and a cohort of 137 ARVC patients (29709087). No variants in ACTC1 were reported.

CDH2 - arrhythmogenic right ventricular cardiomyopathy

Gene:	CDH2 (HGNC:1759)		
Disease:	arrhythmogenic right ventricular cardiomyopathy (MONDO_0016587)	Limited	
Mode of Inheritance:	Autosomal dominant inheritance (HP:0000006)	Classification - 07/13/2018	
Replication over time:	NO	Contradictory Evidence:	NO
Expert Panel:	Arrhythmogenic Right Ventricular Cardiomyopathy EP		

Evidence Summary: There is moderate evidence that CDH2 is associated with ARVC. The first publication that associated TJP1 with ARVC was published in 2017 (28280076) and described two families: one family with five affected with p.Gln229Pro. The phenotype was consistent with ARVC. The second family was one proband with clear ARVC phenotype that carried p.Asp407Asn. A second publication in 2017 also identified in a Norwegian family the same variant, p.Asp407Asn in a family with biventricular ARVC phenotype. There is some experimental evidence in a KO mouse (15662031) that that CDH2 disruption leads to dissolution of desmosomes and area composite, cardiomyopathy, VT, and SCD. However, these are not missense variants as reported in the patients. There is emerging evidence that CDH2 mutations are associated with an ARVC phenotype, but additional information on mechanisms of disease, and pathogenicity of missense vs. truncating variants is still missing.
Gene Clinical Validity Standard Operating Procedures (SOP) - Version 7

			Evidence Type	Case Information Type	Guidelines Default	Range	Max	Count	Points Total	Points Counted	PMIDs/Notes
Genetic Evidence	Case-Level Data	Variant Evidence	Autosomal Dominant or X-linked Disorder	Variant is de novo	2	0-3	12				
				Proband with predicted or proven null variant	1.5	0-2	10				
				Proband with other variant type with some evidence of gene impact	0.5	0-1.5	7	3	2.5	2.5	Mayosi BM et al. 2017 Apr (PMID:28280076); Turkowski KL et al. 2017 Mar (PMID:28326674);
			Autosomal Recessive Disease	Two variants in trans and at least one de novo or a predicted/proven null variant	2	0-3	12				
				Two variants (not predicted/proven null) with some evidence of gene impact in trans	1	0-1.5					
		Segregation Evidence			Summed LOD		Family Count		1	1	
				Candidate gene sequencing							
				Exome/genome or all genes sequenced in linkage region	2.24		1				Mayosi BM et al. 2017 Apr (PMID:28280076);
				Total Summed LOD Score	2.24						
	Case-Control Data		Case-Control Study Type	Case-Control Quality Criteria	Guidelines Points/Study		Max	Count	Points	Points Counted	PMIDs/Notes
			Single Variant Analysis	1. Variant Detection Methodology 2. Power 3. Bias and confounding 4. Statistical Significance	0-6		12				
			Aggregate Variant Analysis		0-6						
			Total Genetic Evidence Points (Maximum 12)							3.5	

	Evidence Category	Evidence Type	Guidelines Default	Range	Max	Count	Points Total	Points Counted	PMIDs/Notes
Experimental Evidence	Function	Biochemical Function	0.5	0 - 2	2				
		Protein Interaction	0.5	0 - 2					
		Expression	0.5	0 - 2					
	Functional Alteration	Patient cells	1	0 - 2	2				
		Non-patient cells	0.5	0 - 1					
	Models	Non-human model organism	2	0 - 4		1	2		Kostetskii I et al. 2005 Feb 18 (PMID:15662031);
		Cell culture model	1	0 - 2					

Rescue	Rescue in human	2	0 - 4	4			2	
	Rescue in non-human model organism	2	0 - 4					
	Rescue in cell culture model	1	0 - 2					
	Rescue in patient cells	1	0 - 2					
**Total Experimental Evidence Points (Maximum 6)**							**2**	

**Assertion criteria**	**Genetic Evidence (0-12 points)**	**Experimental Evidence (0-6 points)**	**Total Points (0-18)**	**Replication Over Time (Y/N)**
**Description**	Case-level, family segregation, or case-control data that support the gene-disease association	Gene-level experimental evidence that support the gene-disease association	Sum of Genetic & Experimental Evidence	> 2 pubs w/ convincing evidence over time (>3 yrs)
**Assigned Points**	**3.5**	**2**	**5.5**	**NO**
**CALCULATED CLASSIFICATION**		**LIMITED**	**1-6**	
		**MODERATE**	**7-11**	
		**STRONG**	**12-18**	
		**DEFINITIVE**	**12-18 AND replication over time**	
**Valid contradictory evidence (Y/N)***	NO			

**CALCULATED CLASSIFICATION (DATE)**	Limited	05/08/2020
**EXPERT CURATION (DATE)**	**Limited**	**07/13/2018**
**EVIDENCE SUMMARY**	**There is moderate evidence that CDH2 is associated with ARVC. The first publication that associated TJP1 with ARVC was published in 2017 (28280076) and described two families: one family with five affected with p.Gln229Pro. The phenotype was consistent with ARVC. The second family was one proband with clear ARVC phenotype that carried p.Asp407Asn. A second publication in 2017 also identified in a Norwegian family the same variant, p.Asp407Asn in a family with biventricular ARVC phenotype. There is some experimental evidence in a KO mouse (15662031) that that CDH2 disruption leads to dissolution of desmosomes and area composite, cardiomyopathy, VT, and SCD. However, these are not missense variants as reported in the patients. There is emerging evidence that CDH2 mutations are associated with an ARVC phenotype, but additional information on mechanisms of disease, and pathogenicity of missense vs. truncating variants is still missing.**	

CTNNA3 - arrhythmogenic right ventricular cardiomyopathy

Gene:	CTNNA3 (HGNC:2511)	**Limited**	
Disease:	arrhythmogenic right ventricular cardiomyopathy (MONDO_0016587)		
Mode of Inheritance:	Autosomal dominant inheritance (HP:0000006)	Classification - 08/06/2019	
Replication over time:	NO	Contradictory Evidence:	NO
Expert Panel:	Arrhythmogenic Right Ventricular Cardiomyopathy EP		

Evidence Summary: CTNNA3: Arrythmogenic Right Ventricular Cardiomyopathy Mode of Inheritance: Autosomal dominant inheritance (HP:0000006) HGNC: 2511 MONDO:0016587 Expert Panel: Arrythmogenic Right Ventricular Cardiomyopathy Three papers were reviewed related to CTNNA3 and ARVD (23136403, 21254927, 224213630). In one paper the CTNNA3 gene was analyzed in 76 ARVD probands that were negative for PKP2, DSP, DSG2, DSC2, and JUP. In one of the probands the heterozygous NM_013266.3(CTNNA3):c.281T>A p.(Val94Asp) variant was found. This variant was not observed in 245296 reference alleles from gnomAD and was not present in both healthy parents and brother suggesting that the variant occurred de novo. Unfortunately no information was given about paternity testing or family history for ARVD. Another proband showed the heterozygous variant NM_013266.3(CTNNA3):c.2296_2298del p.(Leu766del). This variant was not found in 30971 reference alleles from gnomAD but was inherited from her father that only showed a mild right ventricular dilation. Her ant inherited the variant as well but she was asymptomatic. Yeast two-hybrid studies with both variants showed some aberrations (23136403). In another paper a missense variant was found in one of 55 Danish ARVD patients. The variant was found 37 times in 276338 (1 times homozygous) reference alleles in gnomAD making it less likely as a causal variant. In addition the proband did carry a missense variant of unknown significance in DSP (21254927). Germline knockout of alphaT-catenin encoded by ctnna3 in the mouse alters PKP2 distribution without affecting other junctional components of the areae Compositae. These mutant mice exhibit progressive dilated cardiomyopathy, gap junction remodelling, and increased sensitivity to ventricular arrhythmia following acute ischaemia, but not spontaneous ARVC (224213630). Total genetic evidence points calculated = 1.5; Total experimental evidence points calculated = 2; Total awarded points = 3.5; Final classification = Limited.
Gene Clinical Validity Standard Operating Procedures (SOP) - Version 7

			Evidence Type	Case Information Type	Guidelines Default	Guidelines Range	Guidelines Max	Count	Points Total	Points Counted	PMIDs/Notes
Genetic Evidence	Case-Level Data	Variant Evidence	Autosomal Dominant or X-linked Disorder	Variant is de novo	2	0-3	12	1	1.5	1.5	van Hengel J et al. 2013 Jan (PMID:23136403);
				Proband with predicted or proven null variant	1.5	0-2	10				
				Proband with other variant type with some evidence of gene impact	0.5	0-1.5	7				
			Autosomal Recessive Disease	Two variants in trans and at least one de novo or a predicted/proven null variant	2	0-3	12				
				Two variants (not predicted/proven null) with some evidence of gene impact in trans	1	0-1.5					
		Segregation Evidence				Summed LOD		Family Count			
				Candidate gene sequencing							
				Exome/genome or all genes sequenced in linkage region							
				Total Summed LOD Score							

	Case-Control Study Type	Case-Control Quality Criteria	Guidelines Points/Study	Guidelines Max	Points Count	Points Counted	PMIDs/Notes
Case-Control Data	Single Variant Analysis	1. Variant Detection Methodology<br>2. Power<br>3. Bias and confounding<br>4. Statistical Significance	0-6	12			
	Aggregate Variant Analysis		0-6				

Total Genetic Evidence Points (Maximum 12)						1.5	

Evidence Category	Evidence Type	Guidelines Default	Guidelines Range	Guidelines Max	Points Count	Points Total	Points Counted	PMIDs/Notes
Function	Biochemical Function	0.5	0 - 2	2			0.5	
	Protein Interaction	0.5	0 - 2		1	0.5		van Hengel J et al. 2013 Jan (PMID:23136403);
	Expression	0.5	0 - 2					
Functional Alteration	Patient cells	1	0 - 2	2			0.5	
	Non-patient cells	0.5	0 - 1		1	0.5		van Hengel J et al. 2013 Jan (PMID:23136403);
Models	Non-human model organism	2	0 - 4	4	1	1	1	Li J et al. 2012 Feb 15 (PMID:22421363);
	Cell culture model	1	0 - 2					
Rescue	Rescue in human	2	0 - 4					
	Rescue in non-human model organism	2	0 - 4					
	Rescue in cell culture model	1	0 - 2					
	Rescue in patient cells	1	0 - 2					
Total Experimental Evidence Points (Maximum 6)							2	

(Side label: Experimental Evidence)

Assertion criteria	Genetic Evidence (0-12 points)	Experimental Evidence (0-6 points)	Total Points (0-18)	Replication Over Time (Y/N)
Description	Case-level, family segregation, or case-control data that support the gene-disease association	Gene-level experimental evidence that support the gene-disease association	Sum of Genetic & Experimental Evidence	> 2 pubs w/ convincing evidence over time (>3 yrs)
Assigned Points	1.5	2	3.5	NO
CALCULATED CLASSIFICATION		LIMITED	1-6	
		MODERATE	7-11	
		STRONG	12-18	
		DEFINITIVE	12-18 AND replication over time	
Valid contradictory evidence (Y/N)*	NO			
CALCULATED CLASSIFICATION (DATE)		Limited	02/04/2020	
EXPERT CURATION (DATE)		Limited	08/06/2019	
EVIDENCE SUMMARY		CTNNA3: Arrythmogenic Right Ventricular Cardiomyopathy Mode of Inheritance: Autosomal dominant inheritance (HP:0000006) HGNC: 2511 MONDO:0016587 Expert Panel: Arrythmogenic Right Ventricular Cardiomyopathy Three papers were reviewed related to CTNNA3 and ARVD (23136403, 21254927, 224213630). In one paper the CTNNA3 gene was analyzed in 76 ARVD probands that were negative for PKP2, DSP, DSG2, DSC2, and JUP. In one of the probands the heterozygous NM_013266.3(CTNNA3):c.281T>A p.(Val94Asp) variant was found. This variant was not observed in 245296 reference alleles from gnomAD and was not present in both healthy parents and brother suggesting that the variant occurred de novo. Unfortunately no information was given about paternity testing or family history for ARVD. Another proband showed the heterozygous variant NM_013266.3(CTNNA3):c.2296_2298del p.(Leu766del). This variant was not found in 30971 reference alleles from gnomAD but was inherited from her father that only showed a mild right ventricular dilation. Her ant inherited the variant as well but she was asymptomatic. Yeast two-hybrid studies with both variants showed some aberrations (23136403). In another paper a missense variant was found in one of 55 Danish ARVD patients. The variant was found 37 times in 276338 (1 times homozygous) reference alleles in gnomAD making it less likely as a causal variant. In addition the proband did carry a missense variant of unknown significance in DSP (21254927). Germline knockout of alphaT-catenin encoded by ctnna3 in the mouse alters PKP2 distribution without affecting other junctional components of the areae Compositae. These mutant mice exhibit progressive dilated cardiomyopathy, gap junction remodelling, and increased sensitivity to ventricular arrhythmia following acute ischaemia, but not spontaneous ARVC (224213630). Total genetic evidence points calculated = 1.5; Total experimental evidence points calculated = 2; Total awarded points = 3.5; Final classification = Limited.		

DES - arrhythmogenic right ventricular cardiomyopathy

Gene:	DES (HGNC:2770)
Disease:	arrhythmogenic right ventricular cardiomyopathy (MONDO_0016587)
Mode of Inheritance:	Autosomal dominant inheritance (HP:0000006)

Moderate

Classification - 09/11/2018

Replication over time:	NO	Contradictory Evidence:	NO
Expert Panel:	Arrhythmogenic Right Ventricular Cardiomyopathy EP		

Evidence Summary: DES: Arrhythmogenic Right Ventricular Cardiomyopathy (ARVC) HGNC: 2770 MONDO_0016587 Mode of Inheritance: Autosomal dominant inheritance (HP:0000006) Expert Panel: Arrhythmogenic Right Ventricular Cardiomyopathy SOP: Gene Clinical Validity Standard Operating Procedures (SOP), Version 6 Calculated Classification (date) Moderate 09/11/2018 Evidence Summary: The first publication that associated DES variants with ARVC was published in 2009 and reported a missense variant (p.Ser13Phe) in 27 individuals in five families with a severe cardiac phenotype characterized by conduction disease and right ventricular involvement (19879535). Further studies in families with ARVC have identified additional missense variants: the p.Asn342Asp variant was reported in two pedigrees (20423733); p.His326Arg in one family (24200904); p.Pro419Ser in a large Swedish pedigree (22395865) and p.Glu401Asp was detected in a family with 23 affected individuals showing predominant left ventricular arrhythmogenic cardiomyopathy with a high incidence of adverse clinical events (29212896). Two cohort studies of 91 and 22 ARVC index cases respectively have performed genetic screening of DES and identified a total of three missense variants (23168288, 20829228). Immunohistochemistry analysis of cardiac specimens from affected individuals with the p.Arg454Trp or p.Asn116Ser variant has shown severe disruption of desmin distribution at the intercalated discs and formation of cytoplasmic and perinuclear aggregates in cardiomyocytes (20423733, 20829228). Experimental data from three studies support the causative role of DES variants in ARVC (20829228, 22403400, 29212896). In particular, Klauke B et al. characterized the p.Asn116Ser variant in in vitro expression systems using a variety of methods including viscosity analysis of desmin mutant protein and atomic force microscopic imaging. Data from these experiments confirmed the presence of fibrous protein aggregates in mutant cultured cells and were consistent with histological examinations of skeletal and cardiac muscle of ARVC cases carrying the same DES variants (20829228). Similarly, functional studies on the p.Glu401Asp variant in transfected cells showed disruption of cellular adhesion and intermediate filament structure and formation of desmin cytoplasmic aggregates (29212896). Finally, the deleterious structural effects of five heterozygous DES mutations on filament formation in vitro and in living cells were investigated in a study that utilized dual color photoactivation localization microscopy (22403400). In summary, there is moderate evidence to support this gene-disease association. Desmin mutations associated with ARVC appear to be very rare and the observed phenotypes frequently overlap with dilated cardiomyopathy with conduction system abnormalities and occasionally skeletal myopathy.

Gene Clinical Validity Standard Operating Procedures (SOP) - Version 7

	Evidence Type	Case Information Type	Guidelines Default	Guidelines Range	Guidelines Max	Count	Points Total	Points Counted	PMIDs/Notes
Genetic Evidence / Case-Level Data / Variant Evidence	Autosomal Dominant or X-linked Disorder	Variant is de novo	2	0-3	12	1	2	2	Klauke B et al. 2010 Dec 1 (PMID:20829228);
		Proband with predicted or proven null variant	1.5	0-2	10				
		Proband with other variant type with some evidence of gene impact	0.5	0-1.5	7	5	2.5	2.5	van Tintelen JP et al. 2009 Nov (PMID:19879535); Otten E et al. 2010 Aug (PMID:20423733); Brodehl A et al. 2013 Dec (PMID:24200904); Bermúdez-Jiménez FJ et al. 2018 Apr 10 (PMID:29212896);
	Autosomal Recessive Disease	Two variants in trans and at least one de novo or a predicted/proven null variant	2	0-3	12				
		Two variants (not predicted/proven null) with some evidence of gene impact in trans	1	0-1.5					
Genetic Evidence / Case-Level Data	Segregation Evidence			Summed LOD		Family Count	1.5	1.5	
		Candidate gene sequencing		8.43		2			van Tintelen JP et al. 2009 Nov (PMID:19879535); Bermúdez-Jiménez FJ et al. 2018 Apr 10 (PMID:29212896);

	Exome/genome or all genes sequenced in linkage region						
	Total Summed LOD Score	8.43					

Case-Control Study Type	Case-Control Quality Criteria	Guidelines Points/Study	Max	Count	Points	Points Counted	PMIDs/Notes
Single Variant Analysis	1. Variant Detection Methodology	0-6	12				
Aggregate Variant Analysis	2. Power<br>3. Bias and confounding<br>4. Statistical Significance	0-6					
	Total Genetic Evidence Points (Maximum 12)					6	

Case-Control Data

Evidence Category	Evidence Type	Guidelines Default	Range	Max	Count	Points Total	Counted	PMIDs/Notes
Function	Biochemical Function	0.5	0 - 2	2			1.5	
	Protein Interaction	0.5	0 - 2					
	Expression	0.5	0 - 2		3	1.5		van Tintelen JP et al. 2009 Nov (PMID:19879535); Otten E et al. 2010 Aug (PMID:20423733); Klauke B et al. 2010 Dec 1 (PMID:20829228);
Functional Alteration	Patient cells	1	0 - 2	2			2	
	Non-patient cells	0.5	0 - 1		8	4		Klauke B et al. 2010 Dec 1 (PMID:20829228); Bermúdez-Jiménez FJ et al. 2018 Apr 10 (PMID:29212896); Brodehl A et al. 2012 May 4 (PMID:22403400);
Models	Non-human model organism	2	0 - 4	4				
	Cell culture model	1	0 - 2					
Rescue	Rescue in human	2	0 - 4					
	Rescue in non-human model organism	2	0 - 4					
	Rescue in cell culture model	1	0 - 2					
	Rescue in patient cells	1	0 - 2					
	Total Experimental Evidence Points (Maximum 6)						3.5	

Experimental Evidence

Assertion criteria	Genetic Evidence (0-12 points)	Experimental Evidence (0-6 points)	Total Points (0-18)	Replication Over Time (Y/N)
Description	Case-level, family segregation, or case-control data that support the gene-disease association	Gene-level experimental evidence that support the gene-disease association	Sum of Genetic & Experimental Evidence	> 2 pubs w/ convincing evidence over time (>3 yrs)
Assigned Points	**6**	**3.5**	**9.5**	**NO**

CALCULATED CLASSIFICATION		
	LIMITED	1-6
	MODERATE	7-11
	STRONG	12-18
	DEFINITIVE	12-18 AND replication over time

Valid contradictory evidence (Y/N)*	NO	

CALCULATED CLASSIFICATION (DATE)	Moderate	02/04/2020
EXPERT CURATION (DATE)	**Moderate**	09/11/2018

DES: Arrhythmogenic Right Ventricular Cardiomyopathy (ARVC) HGNC: 2770 MONDO_0016587 Mode of Inheritance: Autosomal dominant inheritance (HP:0000006) Expert Panel: Arrhythmogenic Right Ventricular Cardiomyopathy SOP: Gene Clinical Validity Standard Operating Procedures (SOP), Version 6 Calculated Classification (date) Moderate 09/11/2018 Evidence Summary: The first publication that associated DES variants with ARVC was published in 2009 and reported a missense variant (p.Ser13Phe) in 27 individuals in five families with a severe cardiac phenotype characterized by conduction disease and right ventricular involvement (19879535). Further studies in families with ARVC have identified additional missense variants: the p.Asn342Asp variant was reported in two pedigrees (20423733); p.His326Arg in one family (24200904); p.Pro419Ser in a large Swedish pedigree (22395865) and p.Glu401Asp was detected in a family with 23 affected individuals showing predominant left ventricular arrhythmogenic cardiomyopathy with a high incidence of adverse clinical events (29212896). Two cohort studies of 91 and 22 ARVC index cases respectively have performed genetic screening of DES and identified a total of three missense variants (23168288, 20829228). Immunohistochemistry analysis of cardiac specimens from affected individuals with the p.Arg454Trp or p.Asn116Ser variant has shown severe disruption of desmin distribution at the intercalated discs and formation of cytoplasmic and

EVIDENCE SUMMARY	perinuclear aggregates in cardiomyocytes (20423733, 20829228). Experimental data from three studies support the causative role of DES variants in ARVC (20829228, 22403400, 29212896). In particular, Klauke B et al. characterized the p.Asn116Ser variant in in vitro expression systems using a variety of methods including viscosity analysis of desmin mutant protein and atomic force microscopic imaging. Data from these experiments confirmed the presence of fibrous protein aggregates in mutant cultured cells and were consistent with histological examinations of skeletal and cardiac muscle of ARVC cases carrying the same DES variants (20829228). Similarly, functional studies on the p.Glu401Asp variant in transfected cells showed disruption of cellular adhesion and intermediate filament structure and formation of desmin cytoplasmic aggregates (29212896). Finally, the deleterious structural effects of five heterozygous DES mutations on filament formation in vitro and in living cells were investigated in a study that utilized dual color photoactivation localization microscopy (22403400). In summary, there is moderate evidence to support this gene-disease association. Desmin mutations associated with ARVC appear to be very rare and the observed phenotypes frequently overlap with dilated cardiomyopathy with conduction system abnormalities and occasionally skeletal myopathy.

DSC2 - familial isolated arrhythmogenic right ventricular dysplasia

Gene:	DSC2 (HGNC:3036)	**Definitive**	
Disease:	familial isolated arrhythmogenic right ventricular dysplasia (MONDO_0016342)		
Mode of Inheritance:	Autosomal dominant inheritance (HP:0000006)	Classification - 09/14/2018	
Replication over time:	YES	Contradictory Evidence:	NO
Expert Panel:	Arrhythmogenic Right Ventricular Cardiomyopathy EP	Contributors:	UNC Biocuration Core

Evidence Summary: The relationship between DSC2 and arrhythmogenic right ventricular dysplasia (autosomal dominant) and arrhythmogenic right ventricular dysplasia with mild palmoplantar keratoderma with or without woolly hair (autosomal recessive) was evaluated using the ClinGen Clinical Validity Framework as of July, 2018. Variants in DSC2 were first reported in humans with this disease as early as 2006 (Syrris et al., PMID: 17033975). At least 13 variants (e.g. missense, nonsense, frameshift) have been reported in humans. Evidence supporting this gene-disease relationship includes case-level data and experimental data. Summary of Case Level Data: 8.5 points. Variants in this gene have been reported in at least 13 probands in 6 publications (PMIDs: 17963498, 21062920, 23863954, 17186466, 18957847, 17033975). This gene-disease relationship is supported by animal models, expression studies, and protein interactions. In summary, DSC2 is definitively associated with ARVD (AD) and ARVD with mild palmoplantar keratoderma with or without woolly hair (AR). This has been repeatedly demonstrated in both the research and clinical diagnostic settings, and has been upheld over time. This classification was approved by the ClinGen Arrythmogenic Right Ventricular Cardiomyopathy Gene Curation Expert Panel on September 14, 2018 (SOP Version 6).

Gene Clinical Validity Standard Operating Procedures (SOP) - Version 7

Genetic Evidence		Evidence Type		Case Information Type	Guidelines Default	Guidelines Range	Guidelines Max	Count	Points Total	Points Counted	PMIDs/Notes
Case-Level Data	Variant Evidence	Autosomal Dominant or X-linked Disorder		Variant is de novo	2	0-3	12				
				Proband with predicted or proven null variant	1.5	0-2	10	9	7	7	Syrris P et al. 2006 Nov (PMID:17033975); Heuser A et al. 2006 Dec (PMID:17186466); Gerull B et al. 2013 Aug (PMID:23863954); Gehmlich K et al. 2011 Apr 01 (PMID:21062920); Simpson MA et al. 2008 Oct 29 (PMID:18957847);
				Proband with other variant type with some evidence of gene impact	0.5	0-1.5	7	4	1.5	1.5	Beffagna G et al. 2007 Oct 26 (PMID:17963498); Gehmlich K et al. 2011 Apr 01 (PMID:21062920);
		Autosomal Recessive Disease		Two variants in trans and at least one de novo or a predicted/proven null variant	2	0-3	12				
				Two variants (not predicted/proven null) with some evidence of gene impact in trans	1	0-1.5					
	Segregation Evidence				Summed LOD	Family Count					
				Candidate gene sequencing							
				Exome/genome or all genes sequenced in linkage region							
				Total Summed LOD Score							
Case-Control Data	Case-Control Study Type			Case-Control Quality Criteria	Guidelines Points/Study		Max	Count	Points	Counted	PMIDs/Notes
	Single Variant Analysis			1. Variant Detection Methodology<br>2. Power<br>3. Bias and confounding<br>4. Statistical Significance	0-6		12				
	Aggregate Variant Analysis				0-6						
Total Genetic Evidence Points (Maximum 12)										8.5	

Evidence Category	Evidence Type	Guidelines Default	Guidelines Range	Guidelines Max	Count	Points Total	Points Counted	PMIDs/Notes
	Biochemical Function	0.5	0 - 2					

Experimental Evidence	Function	Protein Interaction	0.5	0 - 2	2	1	1	2	Gehmlich K et al. 2011 Apr 01 (PMID:21062920);
		Expression	0.5	0 - 2		2	1		De Bortoli M et al. 2010 Jul (PMID:20197793); Lorimer JE et al. 1994 Oct-Dec (PMID:7711832);
	Functional Alteration	Patient cells	1	0 - 2	2				
		Non-patient cells	0.5	0 - 1					
	Models	Non-human model organism	2	0 - 4	4	1	1.5	3.5	Brodehl A et al. 2017 Mar 24 (PMID:28339476);
		Cell culture model	1	0 - 2					
	Rescue	Rescue in human	2	0 - 4					
		Rescue in non-human model organism	2	0 - 4		1	2		Heuser A et al. 2006 Dec (PMID:17186466);
		Rescue in cell culture model	1	0 - 2					
		Rescue in patient cells	1	0 - 2					
	**Total Experimental Evidence Points (Maximum 6)**							5.5	

Assertion criteria	Genetic Evidence (0-12 points)	Experimental Evidence (0-6 points)	Total Points (0-18)	Replication Over Time (Y/N)
Description	Case-level, family segregation, or case-control data that support the gene-disease association	Gene-level experimental evidence that support the gene-disease association	Sum of Genetic & Experimental Evidence	> 2 pubs w/ convincing evidence over time (>3 yrs)
Assigned Points	**8.5**	**5.5**	**14**	**YES**

CALCULATED CLASSIFICATION	LIMITED	1-6
	MODERATE	7-11
	STRONG	12-18
	DEFINITIVE	12-18 AND replication over time

Valid contradictory evidence (Y/N)*	NO	

CALCULATED CLASSIFICATION (DATE)	Definitive	08/06/2019
EXPERT CURATION (DATE)	**Definitive**	09/14/2018
EVIDENCE SUMMARY	The relationship between DSC2 and arrhythmogenic right ventricular dysplasia (autosomal dominant) and arrhythmogenic right ventricular dysplasia with mild palmoplantar keratoderma with or without woolly hair (autosomal recessive) was evaluated using the ClinGen Clinical Validity Framework as of July, 2018. Variants in DSC2 were first reported in humans with this disease as early as 2006 (Syrris et al., PMID: 17033975). At least 13 variants (e.g. missense, nonsense, frameshift) have been reported in humans. Evidence supporting this gene-disease relationship includes case-level data and experimental data. Summary of Case Level Data: 8.5 points. Variants in this gene have been reported in at least 13 probands in 6 publications (PMIDs: 17963498, 21062920, 23863954, 17186466, 18957847, 17033975). This gene-disease relationship is supported by animal models, expression studies, and protein interactions. In summary, DSC2 is definitively associated with ARVD (AD) and ARVD with mild palmoplantar keratoderma with or without woolly hair (AR). This has been repeatedly demonstrated in both the research and clinical diagnostic settings, and has been upheld over time. This classification was approved by the ClinGen Arrythmogenic Right Ventricular Cardiomyopathy Gene Curation Expert Panel on September 14, 2018 (SOP Version 6).	

DSG2 - arrhythmogenic right ventricular cardiomyopathy

Gene:	DSG2 (HGNC:3049)	Definitive	
Disease:	arrhythmogenic right ventricular cardiomyopathy (MONDO_0016587)		
Mode of Inheritance:	Autosomal dominant inheritance (HP:0000006)	Classification - 09/14/2018	
Replication over time:	YES	Contradictory Evidence:	NO
Expert Panel:	Arrhythmogenic Right Ventricular Cardiomyopathy EP	Contributors:	UNC Biocuration Core

Evidence Summary: The relationship between DSG2 and arrhythmogenic right ventricular cardiomyopathy (autosomal dominant) was evaluated using the ClinGen Clinical Validity Framework as of July, 2018. Variants in DSG2 were first reported in humans with this disease as early as 2006 (Pilchou et al., PMID: 16505173). Variation in DSG2 is a well-known cause of ARVC and accounts for 5%-26% of cases (McNAlly et al., 2005; PMID: 20301310). Since this gene-disease relationship is well-known, there is a significant amount of case-level data, segregation data and experimental data available in the literature, therefore the maximum score for both genetic evidence and experimental evidence has been reached. Note, this curation effort may not be exhaustive of all literature related to this gene-disease relationship. This gene-disease relationship is supported by animal models, in vitro assays, expression assays, and protein interactions. In summary, DSG2 is definitively associated with autosomal dominant ARVC. This has been repeatedly demonstrated in both the research and clinical diagnostic settings, and has been upheld over time. This classification was approved by the ClinGen Arrythmogenic Right Ventricular Cardiomyopathy Gene Curation Expert Panel on September 14, 2018 (SOP Version 6).

Gene Clinical Validity Standard Operating Procedures (SOP) - Version 7

			Evidence Type	Case Information Type	Guidelines Default	Guidelines Range	Guidelines Max	Count	Points Total	Points Counted	PMIDs/Notes
Genetic Evidence	Case-Level Data	Variant Evidence	Autosomal Dominant or X-linked Disorder	Variant is de novo	2	0-3	12	1	2	2	Gandjbakhch E et al. 2009 Mar (PMID:19151369);
				Proband with predicted or proven null variant	1.5	0-2	10	6	8	8	Pilichou K et al. 2006 Mar 07 (PMID:16505173); Awad MM et al. 2006 Jul (PMID:16773573); Syrris P et al. 2007 Mar (PMID:17105751);
				Proband with other variant type with some evidence of gene impact	0.5	0-1.5	7	10	2.3	2.3	Pilichou K et al. 2006 Mar 07 (PMID:16505173); Awad MM et al. 2006 Jul (PMID:16773573); Rasmussen TB et al. 2013 May (PMID:23381804);
			Autosomal Recessive Disease	Two variants in trans and at least one de novo or a predicted/proven null variant	2	0-3	12				
				Two variants (not predicted/proven null) with some evidence of gene impact in trans	1	0-1.5					
		Segregation Evidence			Summed LOD			Family Count			
				Candidate gene sequencing							
				Exome/genome or all genes sequenced in linkage region							
				Total Summed LOD Score							

	Case-Control Study Type	Case-Control Quality Criteria	Guidelines Points/Study	Guidelines Max	Points Count	Points Counted	PMIDs/Notes
Case-Control Data	Single Variant Analysis	1. Variant Detection Methodology<br>2. Power<br>3. Bias and confounding<br>4. Statistical Significance	0-6	12			
	Aggregate Variant Analysis		0-6				
		Total Genetic Evidence Points (Maximum 12)				12	

Evidence Category	Evidence Type	Guidelines Default	Guidelines Range	Guidelines Max	Count	Points Total	Points Counted	PMIDs/Notes
Function	Biochemical Function	0.5	0 - 2	2	1	1	2	Harmon RM et al. 2013 Dec (PMID:24205984);
	Protein Interaction	0.5	0 - 2		1	0.5		Chen X et al. 2002 Mar 22 (PMID:11790773);
	Expression	0.5	0 - 2		1	0.5		Schäfer S et al. 1994 Apr (PMID:8143788);

Experimental Evidence								
Functional Alteration	Patient cells	1	0 - 2	2				
	Non-patient cells	0.5	0 - 1					
Models	Non-human model organism	2	0 - 4	4	3	4	4	Rizzo S et al. 2012 Sep 01 (PMID:22764152); Pilichou K et al. 2009 Aug 03 (PMID:19635863); Kant S et al. 2015 Aug (PMID:26085008);
	Cell culture model	1	0 - 2					
Rescue	Rescue in human	2	0 - 4					
	Rescue in non-human model organism	2	0 - 4					
	Rescue in cell culture model	1	0 - 2					
	Rescue in patient cells	1	0 - 2					
	Total Experimental Evidence Points (Maximum 6)						6	

Assertion criteria	Genetic Evidence (0-12 points)	Experimental Evidence (0-6 points)	Total Points (0-18)	Replication Over Time (Y/N)
Description	Case-level, family segregation, or case-control data that support the gene-disease association	Gene-level experimental evidence that support the gene-disease association	Sum of Genetic & Experimental Evidence	> 2 pubs w/ convincing evidence over time (>3 yrs)
Assigned Points	12	6	18	YES

CALCULATED CLASSIFICATION		
	LIMITED	1-6
	MODERATE	7-11
	STRONG	12-18
	DEFINITIVE	12-18 AND replication over time

Valid contradictory evidence (Y/N)*	NO	

CALCULATED CLASSIFICATION (DATE)	Definitive	08/06/2019
EXPERT CURATION (DATE)	Definitive	09/14/2018
EVIDENCE SUMMARY	The relationship between DSG2 and arrhythmogenic right ventricular cardiomyopathy (autosomal dominant) was evaluated using the ClinGen Clinical Validity Framework as of July, 2018. Variants in DSG2 were first reported in humans with this disease as early as 2006 (Pilchou et al., PMID: 16505173). Variation in DSG2 is a well-known cause of ARVC and accounts for 5%-26% of cases (McNAlly et al., 2005; PMID: 20301310). Since this gene-disease relationship is well-known, there is a significant amount of case-level data, segregation data and experimental data available in the literature, therefore the maximum score for both genetic evidence and experimental evidence has been reached. Note, this curation effort may not be exhaustive of all literature related to this gene-disease relationship. This gene-disease relationship is supported by animal models, in vitro assays, expression assays, and protein interactions. In summary, DSG2 is definitively associated with autosomal dominant ARVC. This has been repeatedly demonstrated in both the research and clinical diagnostic settings, and has been upheld over time. This classification was approved by the ClinGen Arrythmogenic Right Ventricular Cardiomyopathy Gene Curation Expert Panel on September 14, 2018 (SOP Version 6).	

DSP - arrhythmogenic cardiomyopathy with woolly hair and keratoderma

Gene:	DSP (HGNC:3052)	**Definitive**	
Disease:	arrhythmogenic cardiomyopathy with woolly hair and keratoderma (MONDO_0011581)		
Mode of Inheritance:	Autosomal dominant inheritance (HP:0000006)	Classification - 07/12/2019	
Replication over time:	YES	Contradictory Evidence:	NO
Expert Panel:	Arrhythmogenic Right Ventricular Cardiomyopathy EP		

Evidence Summary: The DSP gene was the first ARVC-gene to be associated to the disease, the initial mutation description was done in Carvajal syndrome characterized by woolly hair, keratoderma and ARVC, it is transmitted in a autosomal recessive pattern, homozygous mutations in DSP were described in in the year 2000, PMID 11063735. This was followed by the description of a heterozygous mutation in DSP in an Italian family with ARVC and clear co-segregation of the variant with the disease, PMID 12373648. These findings have been replicated worldwide in several studies performed in different ethnicities, PMID 15941723, PMID 25765472, PMID 23954618, PMID 20864495, PMID 21397041, PMID 24938629. The initial descriptions recognized also a high frequency of left ventricular compromise in families with DSP mutations, PMID 16061754, PMID 28527814. A murine model was generated able to replicate the arrhythmia phenotype and Cx43 mislocalization, PMID 22240500. A transgenic mouse overexpressing a mutant DSP had increased cardiomyocyte apoptosis, cardiac fibrosis and lipid accumulation PMID 16917092. Abnormal DSP protein expression in DSP mutation carriers has also been reported, PMID 23137101. The role of this gene in this particular disease has been repeatedly demonstrated in both the research and clinical diagnostic settings, and has been upheld over time (in general, at least 3 years). No convincing evidence has emerged that contradicts the role of the gene in the specified disease. In summary, based on this overwhelming evidence, DSP is definitely associated with arrhythmogenic cardiomyopathy with woolly hair and keratoderma, maximum association score was achieved rapidly after the analysis of few main reports.

Gene Clinical Validity Standard Operating Procedures (SOP) - Version 7

Genetic Evidence — Case-Level Data — Variant Evidence

Evidence Type	Case Information Type	Guidelines Default	Guidelines Range	Guidelines Max	Points Count	Points Total	Points Counted	PMIDs/Notes
Autosomal Dominant or X-linked Disorder	Variant is de novo	2	0-3	12				
	Proband with predicted or proven null variant	1.5	0-2	10	8	12	10	Castelletti S et al. 2017 Dec 15 (PMID:28527814);
	Proband with other variant type with some evidence of gene impact	0.5	0-1.5	7	2	1	1	Castelletti S et al. 2017 Dec 15 (PMID:28527814); Rampazzo A et al. 2002 Nov (PMID:12373648);
Autosomal Recessive Disease	Two variants in trans and at least one de novo or a predicted/proven null variant	2	0-3	12				
	Two variants (not predicted/proven null) with some evidence of gene impact in trans	1	0-1.5					

Evidence Type	Case Information Type	Summed LOD	Family Count	Points Total	Points Counted	PMIDs/Notes
Segregation Evidence	Candidate gene sequencing	3.91	1	1	1	Rampazzo A et al. 2002 Nov (PMID:12373648);
	Exome/genome or all genes sequenced in linkage region					
	Total Summed LOD Score	3.91				

Case-Control Data

Case-Control Study Type	Case-Control Quality Criteria	Guidelines Points/Study	Guidelines Max	Points Count	Points	Points Counted	PMIDs/Notes
Single Variant Analysis	1. Variant Detection Methodology	0-6	12				
Aggregate Variant Analysis	2. Power<br>3. Bias and confounding<br>4. Statistical Significance	0-6					

Total Genetic Evidence Points (Maximum 12) 12

Evidence Category	Evidence Type	Guidelines Default	Guidelines Range	Guidelines Max	Points Count	Points Total	Points Counted	PMIDs/Notes

Experimental Evidence	Function	Biochemical Function	0.5	0 - 2	2			0.5	
		Protein Interaction	0.5	0 - 2					
		Expression	0.5	0 - 2		1	0.5		Rasmussen TB et al. 2013 Jul (PMID:23137101);
	Functional Alteration	Patient cells	1	0 - 2	2				
		Non-patient cells	0.5	0 - 1					
	Models	Non-human model organism	2	0 - 4	4	1	2	2	Gomes J et al. 2012 Aug (PMID:22240500);
		Cell culture model	1	0 - 2					
	Rescue	Rescue in human	2	0 - 4					
		Rescue in non-human model organism	2	0 - 4					
		Rescue in cell culture model	1	0 - 2					
		Rescue in patient cells	1	0 - 2					
	**Total Experimental Evidence Points (Maximum 6)**							**2.5**	

Assertion criteria	Genetic Evidence (0-12 points)	Experimental Evidence (0-6 points)	Total Points (0-18)	Replication Over Time (Y/N)
**Description**	Case-level, family segregation, or case-control data that support the gene-disease association	Gene-level experimental evidence that support the gene-disease association	Sum of Genetic & Experimental Evidence	> 2 pubs w/ convincing evidence over time (>3 yrs)
**Assigned Points**	**12**	**2.5**	**14.5**	**YES**

**CALCULATED CLASSIFICATION**	**LIMITED**	**1-6**
	**MODERATE**	**7-11**
	**STRONG**	**12-18**
	**DEFINITIVE**	**12-18 AND replication over time**

**Valid contradictory evidence (Y/N)***	NO	

**CALCULATED CLASSIFICATION (DATE)**	Definitive	02/03/2020
**EXPERT CURATION (DATE)**	**Definitive**	**07/12/2019**
**EVIDENCE SUMMARY**	**The DSP gene was the first ARVC-gene to be associated to the disease, the initial mutation description was done in Carvajal syndrome characterized by woolly hair, keratoderma and ARVC, it is transmitted in a autosomal recessive pattern, homozygous mutations in DSP were described in in the year 2000, PMID 11063735. This was followed by the description of a heterozygous mutation in DSP in an Italian family with ARVC and clear co-segregation of the variant with the disease, PMID 12373648. These findings have been replicated worldwide in several studies performed in different ethnicities, PMID 15941723, PMID 25765472, PMID 23954618, PMID 20864495, PMID 21397041, PMID 24938629. The initial descriptions recognized also a high frequency of left ventricular compromise in families with DSP mutations, PMID 16061754, PMID 28527814. A murine model was generated able to replicate the arrhythmia phenotype and Cx43 mislocalization, PMID 22240500. A transgenic mouse overexpressing a mutant DSP had increased cardiomyocyte apoptosis, cardiac fibrosis and lipid accumulation PMID 16917092. Abnormal DSP protein expression in DSP mutation carriers has also been reported, PMID 23137101. The role of this gene in this particular disease has been repeatedly demonstrated in both the research and clinical diagnostic settings, and has been upheld over time (in general, at least 3 years). No convincing evidence has emerged that contradicts the role of the gene in the specified disease. In summary, based on this overwhelming evidence, DSP is definitely associated with arrhythmogenic cardiomyopathy with woolly hair and keratoderma, maximum association score was achieved rapidly after the analysis of few main reports.**	

JUP - arrhythmogenic right ventricular cardiomyopathy

Gene:	JUP (HGNC:6207)	Definitive	
Disease:	arrhythmogenic right ventricular cardiomyopathy (MONDO_0016587)		
Mode of Inheritance:	Autosomal dominant inheritance (HP:0000006)	Classification - 07/27/2018	
Replication over time:	YES	Contradictory Evidence:	NO
Expert Panel:	Arrhythmogenic Right Ventricular Cardiomyopathy EP		

Evidence Summary: Twenty-one papers were reviewed related to JUP and ARVC (2945574, 21668431, 2945574, 9610536, 18937352, 10902626, 15851108, 27170944, 11691526, 16893920, 29802319, 31275992, 25820315, 25820315, 25765472, 25705887, 25087486, 21668431, 20130592, 17924338, 20031617). The initial discovery of a homozygous two-nucleotide deletion (c.2038_2039delTG; p.Trp680Gly_fs) in JUP underlying Naxos disease, which includes ARVC with palmoplantar keratoderma and woolly hair, led to further focus on this and other desmosome genes in non-syndromic ARVC (McCoy et al, PMID 10902626). Murine models support the functional, arrhythmic, and histopathologic cardiac effects from loss-of-function variants in Jup (PMID 27170944; 25705887). Heterozygous pathogenic variants in JUP account for a low percentage of non-syndromic ARVC (0.5%; PMID 25820315). Despite the relatively low percentage, null and missense variants have evidence of impact in multiple families with ARVC. This leads to strong genetic evidence for the role of pathogenic variants in JUP for dominant, non-syndromic ARVC (score 10.5). The experimental evidence supports this association (score 5.5), with final classification: Strong.
Gene Clinical Validity Standard Operating Procedures (SOP) - Version 7

Genetic Evidence			Evidence Type	Case Information Type	Guidelines Default	Range	Max	Count	Points Total	Points Counted	PMIDs/Notes
	Case-Level Data	Variant Evidence	Autosomal Dominant or X-linked Disorder	Variant is de novo	2	0-3	12				
				Proband with predicted or proven null variant	1.5	0-2	10	9	13.5	10	McKoy G et al. 2000 Jun 17 (PMID:10902626);
				Proband with other variant type with some evidence of gene impact	0.5	0-1.5	7	1	0.5	0.5	Asimaki A et al. 2007 Nov (PMID:17924338);
			Autosomal Recessive Disease	Two variants in trans and at least one de novo or a predicted/proven null variant	2	0-3	12				
				Two variants (not predicted/proven null) with some evidence of gene impact in trans	1	0-1.5					
			Segregation Evidence		Summed LOD		Family Count		0	0	
				Candidate gene sequencing	0.6		2				McKoy G et al. 2000 Jun 17 (PMID:10902626);
				Exome/genome or all genes sequenced in linkage region							
				Total Summed LOD Score	0.6						

Genetic Evidence		Case-Control Study Type	Case-Control Quality Criteria	Guidelines Points/Study	Max	Count	Points	Counted	PMIDs/Notes
	Case-Control Data	Single Variant Analysis	1. Variant Detection Methodology<br>2. Power<br>3. Bias and confounding<br>4. Statistical Significance	0-6	12				
		Aggregate Variant Analysis		0-6					
		Total Genetic Evidence Points (Maximum 12)						10.5	

Evidence Type		Guidelines Default	Range	Max	Count	Points Total	Points Counted	PMIDs/Notes
Function	Biochemical Function	0.5	0 - 2	2			0.5	
	Protein Interaction	0.5	0 - 2					
	Expression	0.5	0 - 2		1	0.5		Kaplan SR et al. 2004 May (PMID:15851108);
Functional Alteration	Patient cells	1	0 - 2	2	1	1	1	Kaplan SR et al. 2004 May (PMID:15851108);

Experimental Evidence								
		Non-patient cells	0.5	0 - 1				
	Models	Non-human model organism	2	0 - 4	4	2	4	4	Zhang Z et al. 2015 Apr (PMID:25705887);
		Cell culture model	1	0 - 2					
	Rescue	Rescue in human	2	0 - 4					
		Rescue in non-human model organism	2	0 - 4		1	2		Zhang Z et al. 2015 Apr (PMID:25705887);
		Rescue in cell culture model	1	0 - 2					
		Rescue in patient cells	1	0 - 2					
		Total Experimental Evidence Points (Maximum 6)						5.5	

Assertion criteria	Genetic Evidence (0-12 points)	Experimental Evidence (0-6 points)	Total Points (0-18)	Replication Over Time (Y/N)
Description	Case-level, family segregation, or case-control data that support the gene-disease association	Gene-level experimental evidence that support the gene-disease association	Sum of Genetic & Experimental Evidence	> 2 pubs w/ convincing evidence over time (>3 yrs)
Assigned Points	10.5	5.5	16	YES
CALCULATED CLASSIFICATION		LIMITED	1-6	
		MODERATE	7-11	
		STRONG	12-18	
		DEFINITIVE	12-18 AND replication over time	
Valid contradictory evidence (Y/N)*	NO			
	CALCULATED CLASSIFICATION (DATE)	Definitive	05/13/2020	
	EXPERT CURATION (DATE)	Definitive	07/27/2018	
	EVIDENCE SUMMARY	Twenty-one papers were reviewed related to JUP and ARVC (2945574, 21668431, 2945574, 9610536, 18937352, 10902626, 15851108, 27170944, 11691526, 16893920, 29802319, 31275992, 25820315, 25820315, 25765472, 25705887, 25087486, 21668431, 20130592, 17924338, 20031617). The initial discovery of a homozygous two-nucleotide deletion (c.2038_2039delTG; p.Trp680Gly_fs) in JUP underlying Naxos disease, which includes ARVC with palmoplantar keratoderma and woolly hair, led to further focus on this and other desmosome genes in non-syndromic ARVC (McCoy et al, PMID 10902626). Murine models support the functional, arrhythmic, and histopathologic cardiac effects from loss-of-function variants in Jup (PMID 27170944; 25705887). Heterozygous pathogenic variants in JUP account for a low percentage of non-syndromic ARVC (0.5%; PMID 25820315). Despite the relatively low percentage, null and missense variants have evidence of impact in multiple families with ARVC. This leads to strong genetic evidence for the role of pathogenic variants in JUP for dominant, non-syndromic ARVC (score 10.5). The experimental evidence supports this association (score 5.5), with final classification: Strong.		

LDB3 - arrhythmogenic right ventricular cardiomyopathy

Gene:	LDB3 (HGNC:15710)
Disease:	arrhythmogenic right ventricular cardiomyopathy (MONDO_0016587)
Mode of Inheritance:	Autosomal dominant inheritance (HP:0000006)

Disputed

Classification - 09/12/2019

Replication over time: NO

Contradictory Evidence: NO

Expert Panel: Arrhythmogenic Right Ventricular Cardiomyopathy EP

Evidence Summary: The association of LDB3 with ARVC is disputed. A study (25041374) investigated a family affected by ARVC on which Sanger sequencing failed to find a disease–causing mutation in any of the five desmosomal genes in the proband (DSP, JUP, DSC2, DSG2, PKP2). The authors performed Next Generation Sequencing by using a 134 genes panel in order to discover a candidate disease–causing gene. Genetic screening identified a missense variant on LDB3 causing the aminoacid substitution Thr351Ala, this variant was reported in ClinVar as conflicting interpretation (VUS, LB). This variant shows a high MAF in the general population to be associated with the disease even though the proband and family carriers do have ARVC. There is disputed evidence that the identified LDB3 variant contribute to the ARVC phenotype and there is no known disease mechanism that would link LDB3 with ARVC.

Gene Clinical Validity Standard Operating Procedures (SOP) - Version 7

Genetic Evidence – Case-Level Data – Variant Evidence

Evidence Type	Case Information Type	Guidelines Default	Guidelines Range	Guidelines Max	Points Count	Points Total	Points Counted	PMIDs/Notes
Autosomal Dominant or X-linked Disorder	Variant is de novo	2	0-3	12				
	Proband with predicted or proven null variant	1.5	0-2	10				
	Proband with other variant type with some evidence of gene impact	0.5	0-1.5	7	1	0.1	0.1	Lopez-Ayala JM et al. 2015 Aug (PMID:25041374);
Autosomal Recessive Disease	Two variants in trans and at least one de novo or a predicted/proven null variant	2	0-3	12				
	Two variants (not predicted/proven null) with some evidence of gene impact in trans	1	0-1.5					

Segregation Evidence

Segregation Evidence		Summed LOD	Family Count	Points Total	Points Counted	PMIDs/Notes
	Candidate gene sequencing					
	Exome/genome or all genes sequenced in linkage region					
	Total Summed LOD Score					

Genetic Evidence – Case-Control Data

Case-Control Study Type	Case-Control Quality Criteria	Guidelines Points/Study	Guidelines Max	Points Count	Points Points	Points Counted	PMIDs/Notes
Single Variant Analysis	1. Variant Detection Methodology<br>2. Power<br>3. Bias and confounding<br>4. Statistical Significance	0-6	12				
Aggregate Variant Analysis		0-6					
**Total Genetic Evidence Points (Maximum 12)**						**0.1**	

Experimental Evidence

Evidence Category	Evidence Type	Guidelines Default	Guidelines Range	Guidelines Max	Points Count	Points Total	Points Counted	PMIDs/Notes
Function	Biochemical Function	0.5	0 - 2	2				
	Protein Interaction	0.5	0 - 2					
	Expression	0.5	0 - 2					
Functional Alteration	Patient cells	1	0 - 2	2				
	Non-patient cells	0.5	0 - 1					
Models	Non-human model organism	2	0 - 4					
	Cell culture model	1	0 - 2					

Rescue	Rescue in human	2	0 - 4	4			
	Rescue in non-human model organism	2	0 - 4				
	Rescue in cell culture model	1	0 - 2				
	Rescue in patient cells	1	0 - 2				
Total Experimental Evidence Points (Maximum 6)				0			

Assertion criteria	Genetic Evidence (0-12 points)	Experimental Evidence (0-6 points)	Total Points (0-18)	Replication Over Time (Y/N)
Description	Case-level, family segregation, or case-control data that support the gene-disease association	Gene-level experimental evidence that support the gene-disease association	Sum of Genetic & Experimental Evidence	> 2 pubs w/ convincing evidence over time (>3 yrs)
Assigned Points	0.1	0	0.1	NO

CALCULATED CLASSIFICATION	LIMITED	1-6
	MODERATE	7-11
	STRONG	12-18
	DEFINITIVE	12-18 AND replication over time

Valid contradictory evidence (Y/N)*	NO	

CALCULATED CLASSIFICATION (DATE)	Limited	02/04/2020
MODIFY CALCULATED CLASSIFICATION	YES	
MODIFIED CLASSIFICATION (DATE)	Disputed	02/04/2020
REASON(S) FOR CHANGE	Only a single variant was found, and the MAF of that variant was above the ARVC EP cut-off.	
EXPERT CURATION (DATE)	Disputed	09/12/2019
EVIDENCE SUMMARY	The association of LDB3 with ARVC is disputed. A study (25041374) investigated a family affected by ARVC on which Sanger sequencing failed to find a disease–causing mutation in any of the five desmosomal genes in the proband (DSP, JUP, DSC2, DSG2, PKP2). The authors performed Next Generation Sequencing by using a 134 genes panel in order to discover a candidate disease–causing gene. Genetic screening identified a missense variant on LDB3 causing the aminoacid substitution Thr351Ala, this variant was reported in ClinVar as conflicting interpretation (VUS, LB). This variant shows a high MAF in the general population to be associated with the disease even though the proband and family carriers do have ARVC. There is disputed evidence that the identified LDB3 variant contribute to the ARVC phenotype and there is no known disease mechanism that would link LDB3 with ARVC.	

LMNA - arrhythmogenic right ventricular cardiomyopathy

Gene:	LMNA (HGNC:6636)
Disease:	arrhythmogenic right ventricular cardiomyopathy (MONDO_0016587)
Mode of Inheritance:	Autosomal dominant inheritance (HP:0000006)

Limited

Classification - 09/06/2019

Replication over time:	NO	Contradictory Evidence:	NO
Expert Panel:	Arrhythmogenic Right Ventricular Cardiomyopathy EP		

Evidence Summary: The first publication that associated LMNA variants with ARVC was published in 2012 and reported four patients without desmosomal variants that carried LMNA variants (22199124). These patients met 2010 TCC for ARVC and two had microscopic tissue evaluation confirming interstitial fibrosis and fatty replacement suggesting ARVC phenotype. One variant, p. Arg644Cys under 2015 ACMG criteria no longer is classified as a pathogenic variant. This study also included experimental evidence as immunohistochemistry staining of the biopsies did show delocalization of plakoglobin, which has been reported as pathologic for ARVC phenotype. Another study (25837155) in 2015 showed segregation in a large Italian family; however only the proband met ARVC TFC, the remaining 7 affected individuals only met borderline criteria for ARVC. Another study in 2016 (26620845) identified two LMNA variants in two probands. One only met borderline ARVC criteria with severe RV involvement and frequent PVCs. Experimental evidence was included as RV endomyocardial biopsy did demonstrate fibrous tissue replacement of the myocardium. LMNA mutations associated with ARVC appear to be very rare and the observed phenotypes frequently overlap with dilated cardiomyopathy with conduction system abnormalities and atrial arrhythmias.

Gene Clinical Validity Standard Operating Procedures (SOP) - Version 7

			Evidence Type	Case Information Type	Guidelines Default	Guidelines Range	Guidelines Max	Count	Points Total	Points Counted	PMIDs/Notes
Genetic Evidence	Case-Level Data	Variant Evidence	Autosomal Dominant or X-linked Disorder	Variant is de novo	2	0-3	12				
				Proband with predicted or proven null variant	1.5	0-2	10	1	1.5	1.5	Kato K et al. 2016 Oct (PMID:26620845);
				Proband with other variant type with some evidence of gene impact	0.5	0-1.5	7	4	1.5	1.5	Quarta G et al. 2012 May (PMID:22199124); Kato K et al. 2016 Oct (PMID:26620845);
			Autosomal Recessive Disease	Two variants in trans and at least one de novo or a predicted/proven null variant	2	0-3	12				
				Two variants (not predicted/proven null) with some evidence of gene impact in trans	1	0-1.5					
			Segregation Evidence		Summed LOD		Family Count		1	1	
				Candidate gene sequencing	3.61		4				Quarta G et al. 2012 May (PMID:22199124); Kato K et al. 2016 Oct (PMID:26620845); Forleo C et al. 2015 Apr 2 (PMID:25837155);
				Exome/genome or all genes sequenced in linkage region							
				Total Summed LOD Score	3.61						

	Case-Control Study Type	Case-Control Quality Criteria	Guidelines Points/Study	Guidelines Max	Count	Points	Points Counted	PMIDs/Notes
Case-Control Data	Single Variant Analysis	1. Variant Detection Methodology<br>2. Power<br>3. Bias and confounding<br>4. Statistical Significance	0-6	12				
	Aggregate Variant Analysis		0-6					
	**Total Genetic Evidence Points (Maximum 12)**						4	

Evidence Category	Evidence Type	Guidelines Default	Guidelines Range	Guidelines Max	Count	Points Total	Points Counted	PMIDs/Notes
Function	Biochemical Function	0.5	0 - 2	2				
	Protein Interaction	0.5	0 - 2					

Experimental Evidence								
	Expression	0.5	0 - 2					
Functional Alteration	Patient cells	1	0 - 2	2	1	1	1	Quarta G et al. 2012 May (PMID:22199124);
	Non-patient cells	0.5	0 - 1					
Models	Non-human model organism	2	0 - 4	4				
	Cell culture model	1	0 - 2					
Rescue	Rescue in human	2	0 - 4					
	Rescue in non-human model organism	2	0 - 4					
	Rescue in cell culture model	1	0 - 2					
	Rescue in patient cells	1	0 - 2					
**Total Experimental Evidence Points (Maximum 6)**							**1**	

**Assertion criteria**	**Genetic Evidence (0-12 points)**	**Experimental Evidence (0-6 points)**	**Total Points (0-18)**	**Replication Over Time (Y/N)**
**Description**	Case-level, family segregation, or case-control data that support the gene-disease association	Gene-level experimental evidence that support the gene-disease association	Sum of Genetic & Experimental Evidence	> 2 pubs w/ convincing evidence over time (>3 yrs)
**Assigned Points**	**4**	**1**	**5**	**NO**

**CALCULATED CLASSIFICATION**	**LIMITED**	**1-6**
	**MODERATE**	**7-11**
	**STRONG**	**12-18**
	**DEFINITIVE**	**12-18 AND replication over time**

**Valid contradictory evidence (Y/N)***	NO	

**CALCULATED CLASSIFICATION (DATE)**	**Limited**	**05/08/2020**
**EXPERT CURATION (DATE)**	**Limited**	**09/06/2019**
**EVIDENCE SUMMARY**	**The first publication that associated LMNA variants with ARVC was published in 2012 and reported four patients without desmosomal variants that carried LMNA variants (22199124). These patients met 2010 TCC for ARVC and two had microscopic tissue evaluation confirming interstitial fibrosis and fatty replacement suggesting ARVC phenotype. One variant, p. Arg644Cys under 2015 ACMG criteria no longer is classified as a pathogenic variant. This study also included experimental evidence as immunohistochemistry staining of the biopsies did show delocalization of plakoglobin, which has been reported as pathologic for ARVC phenotype. Another study (25837155) in 2015 showed segregation in a large Italian family; however only the proband met ARVC TFC, the remaining 7 affected individuals only met borderline criteria for ARVC. Another study in 2016 (26620845) identified two LMNA variants in two probands. One only met borderline ARVC criteria with severe RV involvement and frequent PVCs. Experimental evidence was included as RV endomyocardial biopsy did demonstrate fibrous tissue replacement of the myocardium. LMNA mutations associated with ARVC appear to be very rare and the observed phenotypes frequently overlap with dilated cardiomyopathy with conduction system abnormalities and atrial arrhythmias.**	

MYBPC3 - arrhythmogenic right ventricular cardiomyopathy

Gene:	MYBPC3 (HGNC:7551)	**Limited**	
Disease:	arrhythmogenic right ventricular cardiomyopathy (MONDO_0016587)		
Mode of Inheritance:	Autosomal dominant inheritance (HP:0000006)	Classification - 08/06/2019	
Replication over time:	NO	Contradictory Evidence:	NO
Expert Panel:	Arrhythmogenic Right Ventricular Cardiomyopathy EP		

Evidence Summary: MYBPC3: Arrhythmogenic Right Ventricular Cardiomyopathy (ARVC) HGNC: 7551 MONDO_0016587 Mode of Inheritance: Autosomal dominant inheritance (HP:0000006) Expert Panel: Arrhythmogenic Right Ventricular Cardiomyopathy SOP: Gene Clinical Validity Standard Operating Procedures (SOP), Version 6 Calculated Classification (date) Limited 22/02/2019 Evidence Summary: There is limited evidence that MYBPC3 is associated with ARVC. Screening of ARVC patient cohorts for MYBPC3 mutations has been performed in two studies (29709087, 27194543). In these studies, a total of 151 ARVC patients were investigated and three individuals were reported carrying MYBPC3 missense variants. However, the significance of these variants in the context of ARVC is unclear as they were either VUS (e.g. p.Arg238Cys) or MYBPC3 variant carriers were also double heterozygotes for variants in other genes associated with cardiomyopathies (29709087, 27194543). In one ARVC pedigree the identified MYBPC3 p.Phe305Profs variant did not segregate with ARVC in the family (28699631). A recent publication presented a single ARVC case with a missense variant (p.Glu1179Lys) in MYBPC3 (28843747). In summary, there is limited evidence to support this gene-disease association. There is no clear evidence that the identified MYBPC3 variants contribute to the ARVC phenotype and there is no known disease mechanism that would link MYBPC3 with ARVC.
Gene Clinical Validity Standard Operating Procedures (SOP) - Version 7

			Evidence Type	Case Information Type	Guidelines Default	Guidelines Range	Guidelines Max	Count	Points Total	Points Counted	PMIDs/Notes
Genetic Evidence	Case-Level Data	Variant Evidence	Autosomal Dominant or X-linked Disorder	Variant is de novo	2	0-3	12				
				Proband with predicted or proven null variant	1.5	0-2	10				
				Proband with other variant type with some evidence of gene impact	0.5	0-1.5	7	4	1	1	Murray B et al. 2018 Jul (PMID:29709087); Medeiros-Domingo A et al. 2017 Jun 1 (PMID:27194543); Choung HYG et al. 2017 Oct (PMID:28843747);
			Autosomal Recessive Disease	Two variants in trans and at least one de novo or a predicted/proven null variant	2	0-3	12				
				Two variants (not predicted/proven null) with some evidence of gene impact in trans	1	0-1.5					
			Segregation Evidence			Summed LOD		Family Count			
				Candidate gene sequencing							
				Exome/genome or all genes sequenced in linkage region							
				Total Summed LOD Score							

	Case-Control Study Type	Case-Control Quality Criteria	Guidelines Points/Study	Guidelines Max	Points Count	Points Counted	PMIDs/Notes
Case-Control Data	Single Variant Analysis	1. Variant Detection Methodology<br>2. Power<br>3. Bias and confounding<br>4. Statistical Significance	0-6	12			
	Aggregate Variant Analysis		0-6				
		**Total Genetic Evidence Points (Maximum 12)**				1	

Evidence Category	Evidence Type	Guidelines Default	Guidelines Range	Guidelines Max	Count	Points Total	Points Counted	PMIDs/Notes
Function	Biochemical Function	0.5	0 - 2	2				
	Protein Interaction	0.5	0 - 2					

Experimental Evidence		Expression	0.5	0 - 2					
	Functional Alteration	Patient cells	1	0 - 2	2				
		Non-patient cells	0.5	0 - 1					
	Models	Non-human model organism	2	0 - 4	4				
		Cell culture model	1	0 - 2					
	Rescue	Rescue in human	2	0 - 4					
		Rescue in non-human model organism	2	0 - 4					
		Rescue in cell culture model	1	0 - 2					
		Rescue in patient cells	1	0 - 2					
	Total Experimental Evidence Points (Maximum 6)							0	

**Assertion criteria**	**Genetic Evidence (0-12 points)**	**Experimental Evidence (0-6 points)**	**Total Points (0-18)**	**Replication Over Time (Y/N)**
**Description**	Case-level, family segregation, or case-control data that support the gene-disease association	Gene-level experimental evidence that support the gene-disease association	Sum of Genetic & Experimental Evidence	> 2 pubs w/ convincing evidence over time (>3 yrs)
**Assigned Points**	**1**	**0**	**1**	**NO**
**CALCULATED CLASSIFICATION**		**LIMITED**	**1-6**	
		**MODERATE**	**7-11**	
		**STRONG**	**12-18**	
		**DEFINITIVE**	**12-18 AND replication over time**	
**Valid contradictory evidence (Y/N)***	NO			

**CALCULATED CLASSIFICATION (DATE)**	Limited	02/04/2020
**EXPERT CURATION (DATE)**	**Limited**	08/06/2019
**EVIDENCE SUMMARY**	**MYBPC3: Arrhythmogenic Right Ventricular Cardiomyopathy (ARVC) HGNC: 7551 MONDO_0016587 Mode of Inheritance: Autosomal dominant inheritance (HP:0000006) Expert Panel: Arrhythmogenic Right Ventricular Cardiomyopathy SOP: Gene Clinical Validity Standard Operating Procedures (SOP), Version 6 Calculated Classification (date) Limited 22/02/2019 Evidence Summary: There is limited evidence that MYBPC3 is associated with ARVC. Screening of ARVC patient cohorts for MYBPC3 mutations has been performed in two studies (29709087, 27194543). In these studies, a total of 151 ARVC patients were investigated and three individuals were reported carrying MYBPC3 missense variants. However, the significance of these variants in the context of ARVC is unclear as they were either VUS (e.g. p.Arg238Cys) or MYBPC3 variant carriers were also double heterozygotes for variants in other genes associated with cardiomyopathies (29709087, 27194543). In one ARVC pedigree the identified MYBPC3 p.Phe305Profs variant did not segregate with ARVC in the family (28699631). A recent publication presented a single ARVC case with a missense variant (p.Glu1179Lys) in MYBPC3 (28843747). In summary, there is limited evidence to support this gene-disease association. There is no clear evidence that the identified MYBPC3 variants contribute to the ARVC phenotype and there is no known disease mechanism that would link MYBPC3 with ARVC.**	

MYH7 - arrhythmogenic right ventricular cardiomyopathy

Gene:	MYH7 (HGNC:7577)
Disease:	arrhythmogenic right ventricular cardiomyopathy (MONDO_0016587)
Mode of Inheritance:	Autosomal dominant inheritance (HP:0000006)

Limited

Classification - 08/06/2019

Replication over time: NO

Contradictory Evidence: NO

Expert Panel: Arrhythmogenic Right Ventricular Cardiomyopathy EP

Evidence Summary: MYH7: Arrhythmogenic Right Ventricular Cardiomyopathy (ARVC) HGNC: 7577 MONDO_0016587 Mode of Inheritance: Autosomal dominant inheritance (HP:0000006) Expert Panel: Arrhythmogenic Right Ventricular Cardiomyopathy SOP: Gene Clinical Validity Standard Operating Procedures (SOP), Version 6 Calculated Classification (date) Limited 22/03/2019 Evidence Summary: There is limited evidence that MYH7 is associated with ARVC. Screening of ARVC patient cohorts for MYH7 mutations has been performed in four studies (29709087, 27194543, 29253866 and 30385303). In these studies, a total of 245 ARVC patients were investigated and eight individuals were reported carrying MYH7 variants (a deletion and seven missense). However, the significance of these variants in the context of ARVC is unclear as they were either VUS (e.g. p.Arg1846Cys) or previously reported as causative in hypertrophic cardiomyopathy (e.g. p.Lys847del). In one ARVC pedigree the identified MYH7 p.Met877Ile variant did not segregate with ARVC in the family (28699631). In summary, there is limited evidence to support this gene-disease association. There is no clear evidence that the identified MYH7 variants contribute to the ARVC phenotype and there is no known disease mechanism that would link MYH7 with ARVC.
Gene Clinical Validity Standard Operating Procedures (SOP) - Version 7

Genetic Evidence — Case-Level Data — Variant Evidence

Evidence Type	Case Information Type	Guidelines Default	Guidelines Range	Guidelines Max	Count	Points Total	Points Counted	PMIDs/Notes
Autosomal Dominant or X-linked Disorder	Variant is de novo	2	0-3	12				
	Proband with predicted or proven null variant	1.5	0-2	10	1	0.5	0.5	Murray B et al. 2018 Jul (PMID:29709087);
	Proband with other variant type with some evidence of gene impact	0.5	0-1.5	7	5	1.25	1.25	Murray B et al. 2018 Jul (PMID:29709087); Klauke B et al. 2017 Dec 18 (PMID:29253866); Medeiros-Domingo A et al. 2017 Jun 1 (PMID:27194543);
Autosomal Recessive Disease	Two variants in trans and at least one de novo or a predicted/proven null variant	2	0-3	12				
	Two variants (not predicted/proven null) with some evidence of gene impact in trans	1	0-1.5					

Case-Level Data — Segregation Evidence

Evidence Type	Case Information Type	Summed LOD	Family Count	Points Total	Points Counted	PMIDs/Notes
Segregation Evidence	Candidate gene sequencing					
	Exome/genome or all genes sequenced in linkage region					
	Total Summed LOD Score					

Case-Control Data

Case-Control Study Type	Case-Control Quality Criteria	Guidelines Points/Study	Guidelines Max	Count	Points	Points Counted	PMIDs/Notes
Single Variant Analysis	1. Variant Detection Methodology 2. Power 3. Bias and confounding 4. Statistical Significance	0-6	12				
Aggregate Variant Analysis		0-6					

Total Genetic Evidence Points (Maximum 12) 1.75

Evidence Type		Guidelines Default	Guidelines Range	Guidelines Max	Count	Points Total	Points Counted	PMIDs/Notes
Function	Biochemical Function	0.5	0 - 2	2				
	Protein Interaction	0.5	0 - 2					
	Expression	0.5	0 - 2					

Experimental Evidence	Functional Alteration	Patient cells	1	0 - 2	2				
		Non-patient cells	0.5	0 - 1					
	Models	Non-human model organism	2	0 - 4	4				
		Cell culture model	1	0 - 2					
	Rescue	Rescue in human	2	0 - 4					
		Rescue in non-human model organism	2	0 - 4					
		Rescue in cell culture model	1	0 - 2					
		Rescue in patient cells	1	0 - 2					
	**Total Experimental Evidence Points (Maximum 6)**						**0**		

**Assertion criteria**	**Genetic Evidence (0-12 points)**	**Experimental Evidence (0-6 points)**	**Total Points (0-18)**	**Replication Over Time (Y/N)**
**Description**	Case-level, family segregation, or case-control data that support the gene-disease association	Gene-level experimental evidence that support the gene-disease association	Sum of Genetic & Experimental Evidence	> 2 pubs w/ convincing evidence over time (>3 yrs)
**Assigned Points**	**1.75**	**0**	**1.75**	**NO**
**CALCULATED CLASSIFICATION**		**LIMITED**	**1-6**	
		**MODERATE**	**7-11**	
		**STRONG**	**12-18**	
		**DEFINITIVE**	**12-18 AND replication over time**	
**Valid contradictory evidence (Y/N)***	NO			

**CALCULATED CLASSIFICATION (DATE)**	Limited	02/04/2020
**EXPERT CURATION (DATE)**	**Limited**	**08/06/2019**
**EVIDENCE SUMMARY**	**MYH7: Arrhythmogenic Right Ventricular Cardiomyopathy (ARVC) HGNC: 7577 MONDO_0016587 Mode of Inheritance: Autosomal dominant inheritance (HP:0000006) Expert Panel: Arrhythmogenic Right Ventricular Cardiomyopathy SOP: Gene Clinical Validity Standard Operating Procedures (SOP), Version 6 Calculated Classification (date) Limited 22/03/2019 Evidence Summary: There is limited evidence that MYH7 is associated with ARVC. Screening of ARVC patient cohorts for MYH7 mutations has been performed in four studies (29709087, 27194543, 29253866 and 30385303). In these studies, a total of 245 ARVC patients were investigated and eight individuals were reported carrying MYH7 variants (a deletion and seven missense). However, the significance of these variants in the context of ARVC is unclear as they were either VUS (e.g. p.Arg1846Cys) or previously reported as causative in hypertrophic cardiomyopathy (e.g. p.Lys847del). In one ARVC pedigree the identified MYH7 p.Met877Ile variant did not segregate with ARVC in the family (28699631). In summary, there is limited evidence to support this gene-disease association. There is no clear evidence that the identified MYH7 variants contribute to the ARVC phenotype and there is no known disease mechanism that would link MYH7 with ARVC.**	

MYL2 - arrhythmogenic right ventricular cardiomyopathy

Gene:	MYL2 (HGNC:7583)
Disease:	arrhythmogenic right ventricular cardiomyopathy (MONDO_0016587)
Mode of Inheritance:	Autosomal dominant inheritance (HP:0000006)

No Known Disease Relationship

Classification - 05/24/2019

Replication over time:	NO	Contradictory Evidence:	NO
Expert Panel:	Arrhythmogenic Right Ventricular Cardiomyopathy EP		

Evidence Summary: MYL2: Arrhythmogenic Right Ventricular Cardiomyopathy (ARVC) HGNC: 7583 MONDO_0016587 Mode of Inheritance: Autosomal dominant inheritance (HP:0000006) Expert Panel: Arrhythmogenic Right Ventricular Cardiomyopathy SOP: Gene Clinical Validity Standard Operating Procedures (SOP), Version 6 Calculated Classification (date) No reported evidence 22/03/2019 Evidence Summary: There is no evidence that MYL2 is involved in ARVC. This gene has been investigated for pathogenic variants in two populations of gene-elusive ARVC patients: a cohort of 14 Spanish unrelated patients (22421524) and a cohort of 137 ARVC patients meeting 2010 Task Force criteria for ARVC (29709087). No variants in MYL2 were reported.
Gene Clinical Validity Standard Operating Procedures (SOP) - Version 7

			Evidence Type	Case Information Type	Guidelines Default	Guidelines Range	Guidelines Max	Points Count	Points Total	Points Counted	PMIDs/Notes
Genetic Evidence	Case-Level Data	Variant Evidence	Autosomal Dominant or X-linked Disorder	Variant is de novo	2	0-3	12				
				Proband with predicted or proven null variant	1.5	0-2	10				
				Proband with other variant type with some evidence of gene impact	0.5	0-1.5	7				
			Autosomal Recessive Disease	Two variants in trans and at least one de novo or a predicted/proven null variant	2	0-3	12				
				Two variants (not predicted/proven null) with some evidence of gene impact in trans	1	0-1.5					
		Segregation Evidence				Summed LOD	Family Count				
				Candidate gene sequencing							
				Exome/genome or all genes sequenced in linkage region							
				Total Summed LOD Score							

	Case-Control Study Type	Case-Control Quality Criteria	Guidelines Points/Study	Guidelines Max	Points Count	Points Counted	PMIDs/Notes
Case-Control Data	Single Variant Analysis	1. Variant Detection Methodology<br>2. Power<br>3. Bias and confounding<br>4. Statistical Significance	0-6	12			
	Aggregate Variant Analysis		0-6				

Evidence Category

Total Genetic Evidence Points (Maximum 12) 0

Evidence Type	Guidelines	Points	PMIDs/Notes

Experimental Evidence		Default	Range	Max	Count	Total	Counted	
**Function**	Biochemical Function	0.5	0 - 2	2				
	Protein Interaction	0.5	0 - 2					
	Expression	0.5	0 - 2					
**Functional Alteration**	Patient cells	1	0 - 2	2				
	Non-patient cells	0.5	0 - 1					
**Models**	Non-human model organism	2	0 - 4	4				
	Cell culture model	1	0 - 2					
**Rescue**	Rescue in human	2	0 - 4					
	Rescue in non-human model organism	2	0 - 4					
	Rescue in cell culture model	1	0 - 2					
	Rescue in patient cells	1	0 - 2					
**Total Experimental Evidence Points (Maximum 6)**							**0**	

**Assertion criteria**	**Genetic Evidence (0-12 points)**	**Experimental Evidence (0-6 points)**	**Total Points (0-18)**	**Replication Over Time (Y/N)**
**Description**	Case-level, family segregation, or case-control data that support the gene-disease association	Gene-level experimental evidence that support the gene-disease association	Sum of Genetic & Experimental Evidence	> 2 pubs w/ convincing evidence over time (>3 yrs)
**Assigned Points**	**0**	**0**	**0**	**NO**
**CALCULATED CLASSIFICATION**		**LIMITED**	**1-6**	
		**MODERATE**	**7-11**	
		**STRONG**	**12-18**	
		**DEFINITIVE**	**12-18 AND replication over time**	
**Valid contradictory evidence (Y/N)***	NO			

**CALCULATED CLASSIFICATION (DATE)**	**No Known Disease Relationship**	**05/08/2020**
**EXPERT CURATION (DATE)**	**No Known Disease Relationship**	**05/24/2019**
**EVIDENCE SUMMARY**	**MYL2: Arrhythmogenic Right Ventricular Cardiomyopathy (ARVC) HGNC: 7583 MONDO_0016587 Mode of Inheritance: Autosomal dominant inheritance (HP:0000006) Expert Panel: Arrhythmogenic Right Ventricular Cardiomyopathy SOP: Gene Clinical Validity Standard Operating Procedures (SOP), Version 6 Calculated Classification (date) No reported evidence 22/03/2019 Evidence Summary: There is no evidence that MYL2 is involved in ARVC. This gene has been investigated for pathogenic variants in two populations of gene-elusive ARVC patients: a cohort of 14 Spanish unrelated patients (22421524) and a cohort of 137 ARVC patients meeting 2010 Task Force criteria for ARVC (29709087). No variants in MYL2 were reported.**	

MYL3 - arrhythmogenic right ventricular cardiomyopathy

Gene:	MYL3 (HGNC:7584)	Limited	
Disease:	arrhythmogenic right ventricular cardiomyopathy (MONDO_0016587)		
Mode of Inheritance:	Autosomal dominant inheritance (HP:0000006)	Classification - 09/13/2019	
Replication over time:	NO	Contradictory Evidence:	NO
Expert Panel:	Arrhythmogenic Right Ventricular Cardiomyopathy EP		

Evidence Summary: There is limited evidence that MYL3 is associated with ARVC. Screening of ARVC patient cohorts for MYL3 mutations has been performed in two studies (29709087, 22421524). In first study (29709087) 137 ARVC patients were investigated for putative pathogenic variants. A missense variant causing the aminoacid substitution Arg154His was identified in one patient. This previously report variant is classified as likely pathogenic according to current ACMG criteria and has been demonstrate in vitro to disturb the binding of this protein to myosin. However, this variant has not been linked to ARVC phenotype and no segregation data were available. In the second study (22421524) 14 ARVC patients were investigated but no MYL3 variants were reported. There is no clear evidence that the identified MYL3 contribute to the ARVC phenotype and there is no known disease mechanism that would link MYL3 with ARVC.

Gene Clinical Validity Standard Operating Procedures (SOP) - Version 7

		Evidence Type	Case Information Type	Guidelines Default	Guidelines Range	Guidelines Max	Count	Points Total	Points Counted	PMIDs/Notes
Genetic Evidence	Case-Level Data – Variant Evidence	Autosomal Dominant or X-linked Disorder	Variant is de novo	2	0-3	12				
			Proband with predicted or proven null variant	1.5	0-2	10				
			Proband with other variant type with some evidence of gene impact	0.5	0-1.5	7	1	0.25	0.25	Murray B et al. 2018 Jul (PMID:29709087);
		Autosomal Recessive Disease	Two variants in trans and at least one de novo or a predicted/proven null variant	2	0-3	12				
			Two variants (not predicted/proven null) with some evidence of gene impact in trans	1	0-1.5					
	Case-Level Data	Segregation Evidence		Summed LOD		Family Count				
			Candidate gene sequencing							
			Exome/genome or all genes sequenced in linkage region							
			Total Summed LOD Score							

	Case-Control Study Type	Case-Control Quality Criteria	Guidelines Points/Study	Guidelines Max	Count	Points	Points Counted	PMIDs/Notes
Case-Control Data	Single Variant Analysis	1. Variant Detection Methodology 2. Power 3. Bias and confounding 4. Statistical Significance	0-6	12				
	Aggregate Variant Analysis		0-6					
	Total Genetic Evidence Points (Maximum 12)						0.25	

	Evidence Category	Evidence Type	Guidelines Default	Guidelines Range	Guidelines Max	Count	Points Total	Points Counted	PMIDs/Notes
Experimental Evidence	Function	Biochemical Function	0.5	0 - 2	2				
		Protein Interaction	0.5	0 - 2					
		Expression	0.5	0 - 2					
	Functional Alteration	Patient cells	1	0 - 2	2				
		Non-patient cells	0.5	0 - 1					
	Models	Non-human model organism	2	0 - 4					
		Cell culture model	1	0 - 2					

Rescue	Rescue in human	2	0 - 4	4			
	Rescue in non-human model organism	2	0 - 4				
	Rescue in cell culture model	1	0 - 2				
	Rescue in patient cells	1	0 - 2				
Total Experimental Evidence Points (Maximum 6)				0			

Assertion criteria	Genetic Evidence (0-12 points)	Experimental Evidence (0-6 points)	Total Points (0-18)	Replication Over Time (Y/N)
Description	Case-level, family segregation, or case-control data that support the gene-disease association	Gene-level experimental evidence that support the gene-disease association	Sum of Genetic & Experimental Evidence	> 2 pubs w/ convincing evidence over time (>3 yrs)
Assigned Points	0.25	0	0.25	NO

CALCULATED CLASSIFICATION	LIMITED	1-6
	MODERATE	7-11
	STRONG	12-18
	DEFINITIVE	12-18 AND replication over time

Valid contradictory evidence (Y/N)*	NO	

CALCULATED CLASSIFICATION (DATE)	Limited	02/04/2020
EXPERT CURATION (DATE)	Limited	09/13/2019
EVIDENCE SUMMARY	There is limited evidence that MYL3 is associated with ARVC. Screening of ARVC patient cohorts for MYL3 mutations has been performed in two studies (29709087, 22421524). In first study (29709087) 137 ARVC patients were investigated for putative pathogenic variants. A missense variant causing the aminoacid substitution Arg154His was identified in one patient. This previously report variant is classified as likely pathogenic according to current ACMG criteria and has been demonstrate in vitro to disturb the binding of this protein to myosin. However, this variant has not been linked to ARVC phenotype and no segregation data were available. In the second study (22421524) 14 ARVC patients were investigated but no MYL3 variants were reported. There is no clear evidence that the identified MYL3 contribute to the ARVC phenotype and there is no known disease mechanism that would link MYL3 with ARVC.	

PKP2 - arrhythmogenic right ventricular cardiomyopathy

Gene:	PKP2 (HGNC:9024)	Definitive	
Disease:	arrhythmogenic right ventricular cardiomyopathy (MONDO_0016587)		
Mode of Inheritance:	Autosomal dominant inheritance (HP:0000006)	Classification - 03/08/2018	
Replication over time:	YES	Contradictory Evidence:	NO
Expert Panel:	Arrhythmogenic Right Ventricular Cardiomyopathy EP	Contributors:	UNC Biocuration Core

Evidence Summary: The relationship between PKP2 and arrhythmogenic right ventricular cardiomyopathy (autosomal dominant) was evaluated using the ClinGen Clinical Validity Framework as of September, 2018. Variants in PKP2 were first reported in humans with this disease as early as 2004 (Gerull et al., PMID: 15489853). PKP2 is the major causative gene for ARVC and accounts for 34%-74% of cases (McNAlly et al., 2005; PMID: 20301310). There are over 250 PKP2 variants listed in ClinVar for ARVC (missense, nonsense, frameshift, complex rearrangements, etc) (Novelli et al., 2018; PMID: 30619891). This gene-disease relationship is well-known and therefore a significant amount of case-level data, segregation data and experimental data is available in the literature, therefore the maximum score for both genetic evidence and experimental evidence has been reached. Note, this curation effort may not be exhaustive of all literature related to this gene-disease relationship. This gene-disease relationship is supported by animal models, in vitro assays, and protein interactions. In summary, PKP2 is definitively associated with autosomal dominant ARVC. This has been repeatedly demonstrated in both the research and clinical diagnostic settings, and has been upheld over time. This classification was approved by the ClinGen Arrythmogenic Right Ventricular Cardiomyopathy Gene Curation Expert Panel on October, 26, 2018 (SOP Version 6).
Gene Clinical Validity Standard Operating Procedures (SOP) - Version 7

			Evidence Type	Case Information Type	Guidelines Default	Range	Max	Count	Points Total	Points Counted	PMIDs/Notes
Genetic Evidence	Case-Level Data	Variant Evidence	Autosomal Dominant or X-linked Disorder	Variant is de novo	2	0-3	12				
				Proband with predicted or proven null variant	1.5	0-2	10	17	25.5	10	Gerull B et al. 2004 Nov (PMID:15489853); Syrris P et al. 2006 Jan 24 (PMID:16415378);
				Proband with other variant type with some evidence of gene impact	0.5	0-1.5	7	2	1	1	Gerull B et al. 2004 Nov (PMID:15489853); Syrris P et al. 2006 Jan 24 (PMID:16415378);
			Autosomal Recessive Disease	Two variants in trans and at least one de novo or a predicted/proven null variant	2	0-3	12				
				Two variants (not predicted/proven null) with some evidence of gene impact in trans	1	0-1.5					
		Segregation Evidence			Summed LOD		Family Count		1	1	
				Candidate gene sequencing	4.52		3				Syrris P et al. 2006 Jan 24 (PMID:16415378);
				Exome/genome or all genes sequenced in linkage region							
				Total Summed LOD Score	4.52						
	Case-Control Data		Case-Control Study Type	Case-Control Quality Criteria	Guidelines Points/Study		Max	Count	Points	Points Counted	PMIDs/Notes
			Single Variant Analysis	1. Variant Detection Methodology<br>2. Power<br>3. Bias and confounding<br>4. Statistical Significance	0-6		12				
			Aggregate Variant Analysis		0-6						
			Total Genetic Evidence Points (Maximum 12)							12	

Evidence Category	Evidence Type	Guidelines Default	Range	Max	Count	Points Total	Points Counted	PMIDs/Notes
Function	Biochemical Function	0.5	0 - 2	2			2	
	Protein Interaction	0.5	0 - 2		1	1.5		Chen X et al. 2002 Mar 22 (PMID:11790773);
	Expression	0.5	0 - 2		1	0.5		Mertens C et al. 1996 Nov (PMID:8922383);

Experimental Evidence								
Functional Alteration	Patient cells	1	0 - 2	2	1	1	1.5	Caspi O et al. 2013 Dec (PMID:24200905);
	Non-patient cells	0.5	0 - 1		1	0.5		Hall C et al. 2009 (PMID:19533476);
Models	Non-human model organism	2	0 - 4	4	2	3	3	Grossmann KS et al. 2004 Oct 11 (PMID:15479741); Cruz FM et al. 2015 Apr 14 (PMID:25857910);
	Cell culture model	1	0 - 2					
Rescue	Rescue in human	2	0 - 4					
	Rescue in non-human model organism	2	0 - 4					
	Rescue in cell culture model	1	0 - 2					
	Rescue in patient cells	1	0 - 2					
Total Experimental Evidence Points (Maximum 6)							6	

Assertion criteria	Genetic Evidence (0-12 points)	Experimental Evidence (0-6 points)	Total Points (0-18)	Replication Over Time (Y/N)
Description	Case-level, family segregation, or case-control data that support the gene-disease association	Gene-level experimental evidence that support the gene-disease association	Sum of Genetic & Experimental Evidence	> 2 pubs w/ convincing evidence over time (>3 yrs)
Assigned Points	12	6	18	YES

CALCULATED CLASSIFICATION		
	LIMITED	1-6
	MODERATE	7-11
	STRONG	12-18
	DEFINITIVE	12-18 AND replication over time

Valid contradictory evidence (Y/N)*	NO	

CALCULATED CLASSIFICATION (DATE)	Definitive	02/04/2020
EXPERT CURATION (DATE)	**Definitive**	03/08/2018
EVIDENCE SUMMARY	The relationship between PKP2 and arrhythmogenic right ventricular cardiomyopathy (autosomal dominant) was evaluated using the ClinGen Clinical Validity Framework as of September, 2018. Variants in PKP2 were first reported in humans with this disease as early as 2004 (Gerull et al., PMID: 15489853). PKP2 is the major causative gene for ARVC and accounts for 34%-74% of cases (McNAlly et al., 2005; PMID: 20301310). There are over 250 PKP2 variants listed in ClinVar for ARVC (missense, nonsense, frameshift, complex rearrangements, etc) (Novelli et al., 2018; PMID: 30619891). This gene-disease relationship is well-known and therefore a significant amount of case-level data, segregation data and experimental data is available in the literature, therefore the maximum score for both genetic evidence and experimental evidence has been reached. Note, this curation effort may not be exhaustive of all literature related to this gene-disease relationship. This gene-disease relationship is supported by animal models, in vitro assays, and protein interactions. In summary, PKP2 is definitively associated with autosomal dominant ARVC. This has been repeatedly demonstrated in both the research and clinical diagnostic settings, and has been upheld over time. This classification was approved by the ClinGen Arrythmogenic Right Ventricular Cardiomyopathy Gene Curation Expert Panel on October, 26, 2018 (SOP Version 6).	

RYR2 - arrhythmogenic right ventricular cardiomyopathy

Gene:	RYR2 (HGNC:10484)	**Refuted**	
Disease:	arrhythmogenic right ventricular cardiomyopathy (MONDO_0016587)		
Mode of Inheritance:	Autosomal dominant inheritance (HP:0000006)	Classification - 07/19/2019	
Replication over time:	NO	Contradictory Evidence:	YES
Expert Panel:	Arrhythmogenic Right Ventricular Cardiomyopathy EP		

Evidence Summary: Fifty-seven papers were reviewed related to RYR2 and ARVD (9628868, 11078270, 11159936, 11807805, 12015469, 12106942, 12169647, 12459180, 12596074, 12919952, 15046075, 15176428, 15336972, 15451514, 15544015, 15831148, 15951021, 16084945, 16096717, 16239587, 16407108, 16733711, 16769042, 16873551, 16873551, 17363426, 17980246, 18326664, 18483626, 18515204, 19029124, 19167631, 19863545, 20045464, 20301310, 21977247, 22068070, 22090716, 22374134, 22450909, 22453944, 23978697, 24447446, 24978818, 25041964, 25411383, 25445213, 26082335, 26743400, 27000522, 27005929, 27548259, 27635072, 28750076, 29497013, 29543670, Rampazzo A et al., Hum Mol Genet 1995). Three publications from the same research group found linkage to chromosome 1q42-q43 and variants in RYR2 in families with right ventricular cardiomyopathy (ARVC2) also described as CPVT with fibro-fatty replacement of the right ventricle (Rampazzo A et al., Hum Mol Genet 1995; 4(11):2151–2154; 11078270; 11159936). A mouse model made from one of the variants (RyR2 R176Q/_) found in family 105 that has two variants on one allele (R176Q/T2504M), showed a CPVT like phenotype without evidence of fibrofatty infiltration or structural abnormalities characteristic of arrhythmogenic right ventricular dysplasia (16873551). In several papers RYR2 missense variants were described in possible ARVD index patients. Some of these variants were also relatively often present in reference alleles from the gnomAD database, clear ARVD diagnosis is not provided, segregation information is not informative and/or CPVT was also present in the family (28750076, 26743400, 25041964, 16084945, 27005929). In a recent review it was also recognized that the observed phenotype in the original three publications that reported RYR2 variants in ARVD for the first time should be catecholamine-induced ventricular tachycardia rather than ARVD, and this gene is no longer considered as ARVD causing (29543670). This gene is therefore refuted as a candidate for ARVD.
Gene Clinical Validity Standard Operating Procedures (SOP) - Version 7

			Evidence Type	Case Information Type	Guidelines Default	Range	Max	Count	Points Total	Points Counted	PMIDs/Notes
Genetic Evidence	Case-Level Data	Variant Evidence	Autosomal Dominant or X-linked Disorder	Variant is de novo	2	0-3	12				
				Proband with predicted or proven null variant	1.5	0-2	10				
				Proband with other variant type with some evidence of gene impact	0.5	0-1.5	7	8	1.25	1.25	Roux-Buisson N et al. 2014 Nov (PMID:25041964); Deshpande SR et al. 2016 Apr (PMID:26743400); Forleo C et al. 2017 July 27 (PMID:28750076); d'Amati G et al. 2005 Jul (PMID:16084945); Hata Y et al. 2016 July (PMID:27005929); Akilzhanova A et al. 2014 Jun 30 (PMID:24978818);
			Autosomal Recessive Disease	Two variants in trans and at least one de novo or a predicted/proven null variant	2	0-3	12				
				Two variants (not predicted/proven null) with some evidence of gene impact in trans	1	0-1.5					
			Segregation Evidence		Summed LOD		Family Count				
				Candidate gene sequencing							
				Exome/genome or all genes sequenced in linkage region							
				Total Summed LOD Score							

Case-Control Study Type	Case-Control Quality Criteria	Guidelines Points/Study	Max	Count	Points	Counted	PMIDs/Notes
Single Variant Analysis		0-6					
	1. Variant Detection						

Case-Control Data	Aggregate Variant Analysis	Methodology<br>2. Power<br>3. Bias and confounding<br>4. Statistical Significance	0-6	12	1	0	0	Milting H et al. 2006 Aug 1 (PMID:16769042);
	**Total Genetic Evidence Points (Maximum 12)**						**1.25**	

	Evidence Category	Evidence Type	Guidelines Default	Guidelines Range	Max	Count	Points Total	Points Counted	PMIDs/Notes
Experimental Evidence	**Function**	Biochemical Function	0.5	0 - 2	2			0	
		Protein Interaction	0.5	0 - 2		1	0		Tiso N et al. 2002 Dec 13 (PMID:12459180);
		Expression	0.5	0 - 2					
	**Functional Alteration**	Patient cells	1	0 - 2	2				
		Non-patient cells	0.5	0 - 1					
	**Models**	Non-human model organism	2	0 - 4	4				
		Cell culture model	1	0 - 2					
	**Rescue**	Rescue in human	2	0 - 4					
		Rescue in non-human model organism	2	0 - 4					
		Rescue in cell culture model	1	0 - 2					
		Rescue in patient cells	1	0 - 2					
	**Total Experimental Evidence Points (Maximum 6)**							**0**	

**Assertion criteria**	**Genetic Evidence (0-12 points)**	**Experimental Evidence (0-6 points)**	**Total Points (0-18)**	**Replication Over Time (Y/N)**
**Description**	Case-level, family segregation, or case-control data that support the gene-disease association	Gene-level experimental evidence that support the gene-disease association	Sum of Genetic & Experimental Evidence	> 2 pubs w/ convincing evidence over time (>3 yrs)
**Assigned Points**	**1.25**	**0**	**1.25**	**NO**
**CALCULATED CLASSIFICATION**		**LIMITED**	**1-6**	
		**MODERATE**	**7-11**	
		**STRONG**	**12-18**	
		**DEFINITIVE**	**12-18 AND replication over time**	
**Valid contradictory evidence (Y/N)***	YES	Kannankeril PJ et al. 2006 Aug 8 (PMID:16873551);		

**CALCULATED CLASSIFICATION (DATE)**	**Limited**	**05/13/2020**
**MODIFY CALCULATED CLASSIFICATION**	**YES**	
**MODIFIED CLASSIFICATION (DATE)**	**Refuted**	**05/13/2020**
**REASON(S) FOR CHANGE**	**This classification was refuted because the observed phenotype in the original publications that reported RYR2 variants in ARVD should be catecholamine-induced ventricular tachycardia (CPVT) rather than ARVD. Subsequent variants found in this gene in ARVD patients were uninformative or too frequent to be disease-causing.**	
**EXPERT CURATION (DATE)**	**Refuted**	**07/19/2019**

Fifty-seven papers were reviewed related to RYR2 and ARVD (9628868, 11078270, 11159936, 11807805, 12015469, 12106942, 12169647, 12459180, 12596074, 12919952, 15046075, 15176428, 15336972, 15451514, 15544015, 15831148, 15951021, 16084945, 16096717, 16239587, 16407108, 16733711, 16769042, 16873551, 16873551, 17363426, 17980246, 18326664, 18483626, 18515204, 19029124, 19167631, 19863545, 20045464, 20301310, 21977247, 22068070, 22090716, 22374134, 22450909, 22453944, 23978697, 24447446, 24978818, 25041964, 25411383, 25445213, 26082335, 26743400, 27000522, 27005929, 27548259, 27635072, 28750076, 29497013, 29543670, Rampazzo A et al., Hum Mol Genet 1995). Three publications from the same research group found linkage to chromosome 1q42-q43 and variants in RYR2 in families with right ventricular cardiomyopathy (ARVC2) also described as CPVT with fibro-fatty replacement of the right ventricle (Rampazzo A et al., Hum Mol Genet 1995; 4(11):2151–2154; 11078270; 11159936). A mouse model made from one of the variants (RyR2 R176Q/_) found in family 105 that has two variants on one allele (R176Q/T2504M), showed a CPVT like phenotype without evidence of fibrofatty infiltration or structural abnormalities characteristic of arrhythmogenic right ventricular dysplasia (16873551). In several papers RYR2 missense variants were described in possible ARVD index patients. Some of these variants were also relatively often present in reference alleles from the gnomAD database, clear ARVD diagnosis is not provided, segregation information is not

EVIDENCE SUMMARY	informative and/or CPVT was also present in the family (28750076, 26743400, 25041964, 16084945, 27005929). In a recent review it was also recognized that the observed phenotype in the original three publications that reported RYR2 variants in ARVD for the first time should be catecholamine-induced ventricular tachycardia rather than ARVD, and this gene is no longer considered as ARVD causing (29543670). This gene is therefore refuted as a candidate for ARVD.

SCN5A - arrhythmogenic right ventricular cardiomyopathy

Gene:	SCN5A (HGNC:10593)	Limited	Classification - 06/06/2019
Disease:	arrhythmogenic right ventricular cardiomyopathy (MONDO_0016587)		
Mode of Inheritance:	Autosomal dominant inheritance (HP:0000006)		

Replication over time: NO

Contradictory Evidence: NO

Expert Panel: Arrhythmogenic Right Ventricular Cardiomyopathy EP

Evidence Summary: There is limited evidence that SCN5A is associated with ARVC. Screening of ARVC patient cohorts for SCN5A mutations has been performed in four studies (2589161, 24388542, 18375968, 28069705). The first study (2589161) reported the investigation on 6 young patients who were electrically resuscitated after ventricular fibrillation. One of these patients died suddenly during a four years follow-up and autopsy revealed fibrofatty changes in the right ventricle, a decrease number of myocardial cells as typical features of ARVC. Genetic screening revealed a variant on SCN5A, however no further information about genomic localization and classification of the variant was present. The second study (24388542) reported the case of a 50 year old female patient presenting an enlarged right ventricle with structural myocardial changes. Right ventricular angiography showed sacculations in the so-called triangle of dysplasia resembling ARVC structural changes. Genetic testing identified a loss-of-function variant on SCN5A, however no further information about genomic localization and classification of the variant was present. The third study (18375968) reported the case of 58 years old man presenting monomorphic and polymorphic non-sustained ventricular tachycardias, chest pain and dyspnea at rest, complete RBBB, mild dilatation and hypokinesia of the right ventricular and bioptic investigation revealed cardiac hypertrophy and fibrosis, but no myocardial tissue degeneration. Genetic screening identified a splice error on intron 21 of SCN5A gene c.3840+1 G>A, which results in a loss of function of the sodium channel. However, no segregation data were available and patient's phenotype does not fulfil a definite ARVC diagnosis. The most recent study (28069705) investigated the entire exome for pathogenic variants among a 'discovery cohort' of six unrelated Caucasian patients with a clinical diagnosis of ARVC. Genetic screening identified a heterozygous SCN5A missense variant in a female patient causing the aminoacid substitution Arg1898His. Subsequently, authors analysed a 'validation cohort' of 281 unrelated patients for SCN5A variants. Authors identified 5 different variants in 5 patients; among these a missense variant causing the aminoacid substitution Ser1787Asn was excluded due its high frequency in the general population. Two patients were digenic variants carriers, both presenting a different pathogenic variant on PKP2 and an additional variant on SCN5A. Another patient carried a heterozygous in frame deletion (Leu729del), however cascade genetic screening revealed that family members carrying this variants did not fulfill definite ARVC criteria. Finally genetic screening identified a missense variant Tyr416Cys in a 55 years old female but segregation study in the family don't demonstrate the link of this variant with the clinical phenotype. Experimental data from this last study (28069705) analyzed the role of SCN5A Arg1898His variant. Authors generated an induced pluripotent stem cell-derived cardiomyocytes and then corrected them by using CRISP/Cas9 technology. Whole-cell patch clamping revealed a 36% reduction in peak sodium current and super-resolution and fluorescence microscopy showed reduced abundance of both SCN5A and CDH2 clusters at the intercalated disc. However, experimental data do not showed the link of electrical abnormalities to structural changes typical of ARVC alterations such as myocardial fibrotic replacement. In summary, there is limited evidence to support gene-disease association. There is no clear evidence that the identified SCN5A variants contribute to the ARVC phenotype and there is no known disease mechanism that would link SCN5A with ARVC.

Gene Clinical Validity Standard Operating Procedures (SOP) - Version 7

		Evidence Type	Case Information Type	Guidelines			Points			PMIDs/Notes
				Default	Range	Max	Count	Total	Counted	
Case-Level Data	Variant Evidence	Autosomal Dominant or X-linked Disorder	Variant is de novo	2	0-3	12				
			Proband with predicted or proven null variant	1.5	0-2	10	1	1	1	Te Riele AS et al. 2017 Jan (PMID:28069705);
			Proband with other variant type with some evidence of gene impact	0.5	0-1.5	7	6	2	2	Erkapic D et al. 2008 Jul (PMID:18375968); Te Riele AS et al. 2017 Jan (PMID:28069705);
		Autosomal Recessive	Two variants in trans and at least one de novo or a predicted/proven null variant	2	0-3	12				

Genetic Evidence	Disease	Two variants (not predicted/proven null) with some evidence of gene impact in trans	1	0-1.5				
	Segregation Evidence		Summed LOD	Family Count	0	0		
		Candidate gene sequencing	1.2	1			Te Riele AS et al. 2017 Jan (PMID:28069705);	
		Exome/genome or all genes sequenced in linkage region						
		Total Summed LOD Score	1.2					

Case-Control Data	Case-Control Study Type	Case-Control Quality Criteria	Guidelines Points/Study	Max	Count	Points	Points Counted	PMIDs/Notes
	Single Variant Analysis	1. Variant Detection Methodology<br>2. Power<br>3. Bias and confounding<br>4. Statistical Significance	0-6	12				
	Aggregate Variant Analysis		0-6					
	Total Genetic Evidence Points (Maximum 12)						3	

Experimental Evidence	Evidence Category	Evidence Type	Guidelines Default	Range	Max	Count	Points Total	Points Counted	PMIDs/Notes
	Function	Biochemical Function	0.5	0 - 2	2				
		Protein Interaction	0.5	0 - 2					
		Expression	0.5	0 - 2					
	Functional Alteration	Patient cells	1	0 - 2	2	2	2	2	Te Riele AS et al. 2017 Jan (PMID:28069705);
		Non-patient cells	0.5	0 - 1					
	Models	Non-human model organism	2	0 - 4	4			1	
		Cell culture model	1	0 - 2		1	1		Te Riele AS et al. 2017 Jan (PMID:28069705);
	Rescue	Rescue in human	2	0 - 4					
		Rescue in non-human model organism	2	0 - 4					
		Rescue in cell culture model	1	0 - 2					
		Rescue in patient cells	1	0 - 2					
	Total Experimental Evidence Points (Maximum 6)							3	

Assertion criteria	Genetic Evidence (0-12 points)	Experimental Evidence (0-6 points)	Total Points (0-18)	Replication Over Time (Y/N)
Description	Case-level, family segregation, or case-control data that support the gene-disease association	Gene-level experimental evidence that support the gene-disease association	Sum of Genetic & Experimental Evidence	> 2 pubs w/ convincing evidence over time (>3 yrs)
Assigned Points	3	3	6	NO
CALCULATED CLASSIFICATION		LIMITED	1-6	
		MODERATE	7-11	
		STRONG	12-18	
		DEFINITIVE	12-18 AND replication over time	
Valid contradictory evidence (Y/N)*	NO			

CALCULATED CLASSIFICATION (DATE)	Limited	01/24/2020
EXPERT CURATION (DATE)	Limited	06/06/2019

There is limited evidence that SCN5A is associated with ARVC. Screening of ARVC patient cohorts for SCN5A mutations has been performed in four studies (2589161, 24388542, 18375968, 28069705). The first study (2589161) reported the investigation on 6 young patients who were electrically resuscitated after ventricular fibrillation. One of these patients died suddenly during a four years follow-up and autopsy revealed fibrofatty changes in the right ventricle, a decrease number of myocardial cells as typical features of ARVC. Genetic screening revealed a variant on SCN5A, however no further information about genomic localization and classification of the variant was present. The second study (24388542)

EVIDENCE SUMMARY	reported the case of a 50 year old female patient presenting an enlarged right ventricle with structural myocardial changes. Right ventricular angiography showed sacculations in the so-called triangle of dysplasia resembling ARVC structural changes. Genetic testing identified a loss-of-function variant on SCN5A, however no further information about genomic localization and classification of the variant was present. The third study (18375968) reported the case of 58 years old man presenting monomorphic and polymorphic non-sustained ventricular tachycardias, chest pain and dyspnea at rest, complete RBBB, mild dilatation and hypokinesia of the right ventricular and bioptic investigation revealed cardiac hypertrophy and fibrosis, but no myocardial tissue degeneration. Genetic screening identified a splice error on intron 21 of SCN5A gene c.3840+1 G>A, which results in a loss of function of the sodium channel. However, no segregation data were available and patient's phenotype does not fulfil a definite ARVC diagnosis. The most recent study (28069705) investigated the entire exome for pathogenic variants among a 'discovery cohort' of six unrelated Caucasian patients with a clinical diagnosis of ARVC. Genetic screening identified a heterozygous SCN5A missense variant in a female patient causing the aminoacid substitution Arg1898His. Subsequently, authors analysed a 'validation cohort' of 281 unrelated patients for SCN5A variants. Authors identified 5 different variants in 5 patients; among these a missense variant causing the aminoacid substitution Ser1787Asn was excluded due its high frequency in the general population. Two patients were digenic variants carriers, both presenting a different pathogenic variant on PKP2 and an additional variant on SCN5A. Another patient carried a heterozygous in frame deletion (Leu729del), however cascade genetic screening revealed that family members carrying this variants did not fulfill definite ARVC criteria. Finally genetic screening identified a missense variant Tyr416Cys in a 55 years old female but segregation study in the family don't demonstrate the link of this variant with the clinical phenotype. Experimental data from this last study (28069705) analyzed the role of SCN5A Arg1898His variant. Authors generated an induced pluripotent stem cell-derived cardiomyocytes and then corrected them by using CRISP/Cas9 technology. Whole-cell patch clamping revealed a 36% reduction in peak sodium current and super-resolution and fluorescence microscopy showed reduced abundance of both SCN5A and CDH2 clusters at the intercalated disc. However, experimental data do not showed the link of electrical abnormalities to structural changes typical of ARVC alterations such as myocardial fibrotic replacement. In summary, there is limited evidence to support gene-disease association. There is no clear evidence that the identified SCN5A variants contribute to the ARVC phenotype and there is no known disease mechanism that would link SCN5A with ARVC.

TGFB3 - arrhythmogenic right ventricular cardiomyopathy

Gene:	TGFB3 (HGNC:11769)
Disease:	arrhythmogenic right ventricular cardiomyopathy (MONDO_0016587)
Mode of Inheritance:	Autosomal dominant inheritance (HP:0000006)

Limited

Classification - 08/16/2019

Replication over time: NO

Contradictory Evidence: NO

Expert Panel: Arrhythmogenic Right Ventricular Cardiomyopathy EP

Evidence Summary: TGFB: Arrythmogenic Right Ventricular Cardiomyopathy Mode of Inheritance: Autosomal dominant inheritance (HP:0000006) HGNC: 11769 MONDO:0016587 Expert Panel: Arrythmogenic Right Ventricular Cardiomyopathy Eleven papers were reviewed related to TGFB and ARVD (7951245, 15639475, 20864495, 222232248, 20124997, 15639468, 11127465, 12529708, 22878021, 24238504, Rampazzo A. Cardiovasc Res. 2012; 96: 191-194). It all started in 1994 with linkage to markers on chromosome 14q42.3 in a large Italian family (7951245) and followed by linkage to this region in several other Italian families and sequence analysis of candidate genes by the same group in 2000, 2003, 2005 and reviewed in 2012 (11127465, 12529708, 15639475, Rampazzo A. Cardiovasc Res. 2012; 96: 191-194). In six of 28 families linkage to 14q23-q24 was found; in two families, significantly positive lod scores were obtained with markers closely linked to ARVD1 locus (11127465). One family 112 had a LOD score of 5.2 (affected only LOD score of 2.71) another family 119 had a LOD score of 1.51. The critical interval for ARVD1 contained 40 known genes; 6 of them (POMT2, KIAA0759, KIAA1036, C14orf4, TAIL1 and TGFb3) were unsuccessfully screened for pathogenic ARVC mutations in the coding exons (12529708). In family 112 a non-coding variant (Chr14(GRCh37):g.76447266C>T; NM_003239.4(TGFB3):c.-30G>A, in the paper indicated as c.-36G>A) was identified in TGFB. This variant is observed 10 times in 249028 reference alleles from gnomAD. In an unrelated ARVD proband a TGFB variant was found in the 3′UTR (Chr14(GRCh37):g.76425035G>A: NM_003239.4(TGFB3):c.*495C>T, in the paper indicated as c.1723C>T) . This variant was not observed in 30272 reference alleles from gnomAD. Both variants were tested in a Luciferase C2C12 expression assay and both showed a 2.5 times increased activity compared to wildtype (15639475). In the review paper Rampazzo describes that in two unrelated families with linkage to this region showed no mutations in TGFB3 coding sequences, UTRs and promoter region and that in one of these two families, a large deletion encompassing the entire PKP2 gene was identified (15639475). Two coding variants of unknown significance were observed in this gene in two ARVD probands in another study (24238504). Total genetic evidence points calculated = 1.2; Total experimental evidence points calculated = 1; Total awarded points = 2.2; Final classification = Limited.
Gene Clinical Validity Standard Operating Procedures (SOP) - Version 7

			Evidence Type	Case Information Type	Guidelines Default	Guidelines Range	Guidelines Max	Count	Points Total	Points Counted	PMIDs/Notes
Genetic Evidence	Case-Level Data	Variant Evidence	Autosomal Dominant or X-linked Disorder	Variant is de novo	2	0-3	12				
				Proband with predicted or proven null variant	1.5	0-2	10				
				Proband with other variant type with some evidence of gene impact	0.5	0-1.5	7	2	0.2	0.2	Beffagna G et al. 2005 Feb 1 (PMID:15639475);
			Autosomal Recessive Disease	Two variants in trans and at least one de novo or a predicted/proven null variant	2	0-3	12				
				Two variants (not predicted/proven null) with some evidence of gene impact in trans	1	0-1.5					
			Segregation Evidence			Summed LOD		Family Count			
				Candidate gene sequencing		4.41		1	1	1	Beffagna G et al. 2005 Feb 1 (PMID:15639475);
				Exome/genome or all genes sequenced in linkage region							
				Total Summed LOD Score		4.41					

Case-Control Study Type	Case-Control Quality Criteria	Guidelines Points/Study	Guidelines Max	Count	Points Counted	PMIDs/Notes

Case-Control Data	Single Variant Analysis	1. Variant Detection Methodology 2. Power 3. Bias and confounding 4. Statistical Significance	0-6		12				
	Aggregate Variant Analysis		0-6						
Total Genetic Evidence Points (Maximum 12)								1.2	

	Evidence Category	Evidence Type	Guidelines Default	Range	Max	Points Count	Total	Counted	PMIDs/Notes
Experimental Evidence	Function	Biochemical Function	0.5	0 - 2	2				
		Protein Interaction	0.5	0 - 2					
		Expression	0.5	0 - 2					
	Functional Alteration	Patient cells	1	0 - 2	2			1	
		Non-patient cells	0.5	0 - 1		1	1		Beffagna G et al. 2005 Feb 1 (PMID:15639475);
	Models	Non-human model organism	2	0 - 4	4				
		Cell culture model	1	0 - 2					
	Rescue	Rescue in human	2	0 - 4					
		Rescue in non-human model organism	2	0 - 4					
		Rescue in cell culture model	1	0 - 2					
		Rescue in patient cells	1	0 - 2					
Total Experimental Evidence Points (Maximum 6)								1	

Assertion criteria	Genetic Evidence (0-12 points)	Experimental Evidence (0-6 points)	Total Points (0-18)	Replication Over Time (Y/N)
Description	Case-level, family segregation, or case-control data that support the gene-disease association	Gene-level experimental evidence that support the gene-disease association	Sum of Genetic & Experimental Evidence	> 2 pubs w/ convincing evidence over time (>3 yrs)
Assigned Points	**1.2**	**1**	**2.2**	**NO**
CALCULATED CLASSIFICATION		LIMITED	1-6	
		MODERATE	7-11	
		STRONG	12-18	
		DEFINITIVE	12-18 AND replication over time	
Valid contradictory evidence (Y/N)*	NO			
	CALCULATED CLASSIFICATION (DATE)	Limited	02/04/2020	
	EXPERT CURATION (DATE)	**Limited**	08/16/2019	

TGFB: Arrythmogenic Right Ventricular Cardiomyopathy Mode of Inheritance: Autosomal dominant inheritance (HP:0000006) HGNC: 11769 MONDO:0016587 Expert Panel: Arrythmogenic Right Ventricular Cardiomyopathy Eleven papers were reviewed related to TGFB and ARVD (7951245, 15639475, 20864495, 222232248, 20124997, 15639468, 11127465, 12529708, 22878021, 24238504, Rampazzo A. Cardiovasc Res. 2012; 96: 191-194). It all started in 1994 with linkage to markers on chromosome 14q42.3 in a large Italian family (7951245) and followed by linkage to this region in several other Italian families and sequence analysis of candidate genes by the same group in 2000, 2003, 2005 and reviewed in 2012 (11127465, 12529708, 15639475, Rampazzo A. Cardiovasc Res. 2012; 96: 191-194). In six of 28 families linkage to 14q23-q24 was found; in two families, significantly positive lod scores were obtained with markers closely linked to ARVD1 locus (11127465). One family 112 had a LOD score of 5.2 (affected only LOD score of 2.71) another family 119 had a LOD score of 1.51. The critical interval for ARVD1 contained 40 known genes; 6 of them (POMT2, KIAA0759, KIAA1036, C14orf4, TAIL1 and TGFb3) were unsuccessfully screened for pathogenic ARVC mutations in the coding exons (12529708). In family 112 a non-coding variant (Chr14(GRCh37):g.76447266C>T; NM_003239.4(TGFB3):c.-30G>A, in the paper indicated as c.-36G>A) was identified in TGFB. This variant is observed 10 times in 249028 reference alleles from gnomAD. In an unrelated ARVD proband a TGFB variant was found in the 3′UTR (Chr14(GRCh37):g.76425035G>A: NM_003239.4(TGFB3):c.*495C>T, in the paper indicated as c.1723C>T) . This variant was not observed in 30272 reference alleles from gnomAD. Both variants were tested in a Luciferase C2C12 expression assay and both showed a 2.5 times increased activity compared to wildtype (15639475). In the review paper Rampazzo describes that in two unrelated families with linkage to this region showed no mutations in TGFB3 coding sequences, UTRs and promoter region and that in one of these two families, a large deletion encompassing the entire PKP2

EVIDENCE SUMMARY	gene was identified (15639475). Two coding variants of unknown significance were observed in this gene in two ARVD probands in another study (24238504). Total genetic evidence points calculated = 1.2; Total experimental evidence points calculated = 1; Total awarded points = 2.2; Final classification = Limited.

TJP1 - arrhythmogenic right ventricular cardiomyopathy

Gene:	TJP1 (HGNC:11827)
Disease:	arrhythmogenic right ventricular cardiomyopathy (MONDO_0016587)
Mode of Inheritance:	Autosomal dominant inheritance (HP:0000006)

Limited

Classification - 02/08/2019

Replication over time:	NO	Contradictory Evidence:	NO
Expert Panel:	Arrhythmogenic Right Ventricular Cardiomyopathy EP		

Evidence Summary: There is limited evidence that TJP1 is associated with ARVC. Published in 2018 (30354300), there was one family meeting 2010 TFC that was found to have a variant in TJP1: p.Y669C. Targeted sequencing of an additional 40 ACM patients found another patient with a variant in TJP1 p.R265W. An additional 43 Dutch/German patients were sequenced and in 2 patients with DCM and VT, TJP1 variants were found but one had a pathogenic DSP variant. Of these cases, two probands clearly met 2010 TFC. One proband had histology consistent with ARVC. Functional characterization in this paper showed that it interacts with the gap junction, but not more specific phenotype. Therefore, limited experimental studies, and lack of proof of segregation in families leaves TJP1 with only limited evidence for ARVC.

Gene Clinical Validity Standard Operating Procedures (SOP) - Version 7

Evidence	Evidence Type		Case Information Type	Guidelines Default	Guidelines Range	Guidelines Max	Count	Points Total	Points Counted	PMIDs/Notes
Genetic Evidence / Case-Level Data / Variant Evidence	Autosomal Dominant or X-linked Disorder		Variant is de novo	2	0-3	12				
			Proband with predicted or proven null variant	1.5	0-2	10				
			Proband with other variant type with some evidence of gene impact	0.5	0-1.5	7	2	1	1	De Bortoli M et al. 2018 Oct (PMID:30354300);
	Autosomal Recessive Disease		Two variants in trans and at least one de novo or a predicted/proven null variant	2	0-3	12				
			Two variants (not predicted/proven null) with some evidence of gene impact in trans	1	0-1.5					
Genetic Evidence / Case-Level Data	Segregation Evidence				Summed LOD		Family Count	0	0	
			Candidate gene sequencing							
			Exome/genome or all genes sequenced in linkage region		0.9		2			De Bortoli M et al. 2018 Oct (PMID:30354300);
			Total Summed LOD Score		0.9					

Evidence	Case-Control Study Type	Case-Control Quality Criteria	Guidelines Points/Study	Guidelines Max	Count	Points	Counted	PMIDs/Notes
Genetic Evidence / Case-Control Data	Single Variant Analysis	1. Variant Detection Methodology 2. Power 3. Bias and confounding 4. Statistical Significance	0-6	12				
	Aggregate Variant Analysis		0-6					
	**Total Genetic Evidence Points (Maximum 12)**						1	

Evidence	Evidence Category	Evidence Type	Guidelines Default	Guidelines Range	Guidelines Max	Count	Points Total	Points Counted	PMIDs/Notes
Experimental Evidence	Function	Biochemical Function	0.5	0 - 2	2				
		Protein Interaction	0.5	0 - 2					
		Expression	0.5	0 - 2					
	Functional Alteration	Patient cells	1	0 - 2	2				
		Non-patient cells	0.5	0 - 1					
	Models	Non-human model organism	2	0 - 4					
		Cell culture model	1	0 - 2					
		Rescue in human	2	0 - 4					

**Rescue**	Rescue in non-human model organism	2	0 - 4	4				
	Rescue in cell culture model	1	0 - 2					
	Rescue in patient cells	1	0 - 2					
**Total Experimental Evidence Points (Maximum 6)**						**0**		

**Assertion criteria**	**Genetic Evidence (0-12 points)**	**Experimental Evidence (0-6 points)**	**Total Points (0-18)**	**Replication Over Time (Y/N)**
**Description**	Case-level, family segregation, or case-control data that support the gene-disease association	Gene-level experimental evidence that support the gene-disease association	Sum of Genetic & Experimental Evidence	> 2 pubs w/ convincing evidence over time (>3 yrs)
**Assigned Points**	**1**	**0**	**1**	**NO**
**CALCULATED CLASSIFICATION**		**LIMITED**	**1-6**	
		**MODERATE**	**7-11**	
		**STRONG**	**12-18**	
		**DEFINITIVE**	**12-18 AND replication over time**	
**Valid contradictory evidence (Y/N)***	NO			
**CALCULATED CLASSIFICATION (DATE)**		**Limited**	**05/08/2020**	
**EXPERT CURATION (DATE)**		**Limited**	**02/08/2019**	
**EVIDENCE SUMMARY**		**There is limited evidence that TJP1 is associated with ARVC. Published in 2018 (30354300), there was one family meeting 2010 TFC that was found to have a variant in TJP1: p.Y669C. Targeted sequencing of an additional 40 ACM patients found another patient with a variant in TJP1 p.R265W. An additional 43 Dutch/German patients were sequenced and in 2 patients with DCM and VT, TJP1 variants were found but one had a pathogenic DSP variant. Of these cases, two probands clearly met 2010 TFC. One proband had histology consistent with ARVC. Functional characterization in this paper showed that it interacts with the gap junction, but not more specific phenotype. Therefore, limited experimental studies, and lack of proof of segregation in families leaves TJP1 with only limited evidence for ARVC.**		

TMEM43 - arrhythmogenic right ventricular dysplasia 5

Gene:	TMEM43 (HGNC:28472)
Disease:	arrhythmogenic right ventricular dysplasia 5 (MONDO_0011459)
Mode of Inheritance:	Autosomal dominant inheritance (HP:0000006)

Definitive

Classification - 10/26/2018

Replication over time:	YES	Contradictory Evidence:	NO
Expert Panel:	Arrhythmogenic Right Ventricular Cardiomyopathy EP	Contributors:	UNC Biocuration Core

Evidence Summary: The relationship between TMEM43 and arrhythmogenic right ventricular dysplasia (autosomal dominant) was evaluated using the ClinGen Clinical Validity Framework as of July 10th, 2019. Variants in TMEM43 were first reported in humans with this disease as early as 2008 (Merner et al., PMID 18313022). At least 9 variants (mostly missense) have been reported in humans. However, the pathogenicity of most of the variants is unknown. The majority of genetic evidence comes from case-level data and segregation data for one founder variant, p.Ser358Leu, which has been reported in more than 20 families with ARVC and occurred de novo in one individual (Merner et al., 2008, PMID 18313022; Christensen et al. 2011, PMID 21214875; Baskin et al., 2013, PMID 23812740; Hodgkinson et al., 2013, PMID 22725725; Milting et al., 2014, PMID 24598986). This gene-disease relationship is also supported by an animal model, expression stuies, and in vitro assays. In summary, TMEM43 is definitively associated with autosomal dominant arrhythmogenic right ventricular dysplasia . This has been repeatedly demonstrated in both the research and clinical diagnostic settings, and has been upheld over time. This classification was approved by the ClinGen Arrythmogenic Right Ventricular Cardiomyopathy Gene Curation Expert Panel on October 26, 2018 (SOP Version 6).
Gene Clinical Validity Standard Operating Procedures (SOP) - Version 7

<table>
<tr><th colspan="4">Evidence Type</th><th>Case Information Type</th><th>Guidelines Default</th><th>Range</th><th>Max</th><th>Count</th><th>Points Total</th><th>Counted</th><th>PMIDs/Notes</th></tr>
<tr><td rowspan="9">Genetic Evidence</td><td rowspan="9">Case-Level Data</td><td rowspan="5">Variant Evidence</td><td rowspan="3">Autosomal Dominant or X-linked Disorder</td><td>Variant is de novo</td><td>2</td><td>0-3</td><td>12</td><td>1</td><td>3</td><td>3</td><td>Baskin B et al. 2013 Nov (PMID:23812740);</td></tr>
<tr><td>Proband with predicted or proven null variant</td><td>1.5</td><td>0-2</td><td>10</td><td></td><td></td><td></td><td></td></tr>
<tr><td>Proband with other variant type with some evidence of gene impact</td><td>0.5</td><td>0-1.5</td><td>7</td><td>16</td><td>3.6</td><td>3.6</td><td>Merner ND et al. 2008 Apr (PMID:18313022); Christensen AH et al. 2011 Sep (PMID:21214875); Baskin B et al. 2013 Nov (PMID:23812740); Honda T et al. 2016 May (PMID:26840987); Milting H et al. 2015 Apr 7 (PMID:24598986); Haywood AF et al. 2013 Apr (PMID:23161701);</td></tr>
<tr><td rowspan="2">Autosomal Recessive Disease</td><td>Two variants in trans and at least one de novo or a predicted/proven null variant</td><td>2</td><td>0-3</td><td rowspan="2">12</td><td></td><td></td><td rowspan="2"></td><td></td></tr>
<tr><td>Two variants (not predicted/proven null) with some evidence of gene impact in trans</td><td>1</td><td>0-1.5</td><td></td><td></td><td></td></tr>
<tr><td rowspan="4" colspan="2">Segregation Evidence</td><td></td><td colspan="2">Summed LOD</td><td colspan="2">Family Count</td><td rowspan="4">1.5</td><td rowspan="4">1.5</td><td></td></tr>
<tr><td>Candidate gene sequencing</td><td colspan="2">26.49</td><td colspan="2">2</td><td>Merner ND et al. 2008 Apr (PMID:18313022); Milting H et al. 2015 Apr 7 (PMID:24598986);</td></tr>
<tr><td>Exome/genome or all genes sequenced in linkage region</td><td colspan="2"></td><td colspan="2"></td><td></td></tr>
<tr><td>Total Summed LOD Score</td><td colspan="2">26.49</td><td colspan="2"></td><td></td></tr>
<tr><th></th><th rowspan="3">Case-Control Data</th><th colspan="2">Case-Control Study Type</th><th>Case-Control Quality Criteria</th><th colspan="2">Guidelines Points/Study</th><th>Max</th><th>Count</th><th colspan="2">Points Counted</th><th>PMIDs/Notes</th></tr>
<tr><td></td><td colspan="2">Single Variant Analysis</td><td rowspan="2">1. Variant Detection Methodology
2. Power
3. Bias and confounding
4. Statistical Significance</td><td colspan="2">0-6</td><td rowspan="2">12</td><td></td><td></td><td rowspan="2"></td><td></td></tr>
<tr><td></td><td colspan="2">Aggregate Variant Analysis</td><td colspan="2">0-6</td><td></td><td></td><td></td></tr>
<tr><td colspan="10">Total Genetic Evidence Points (Maximum 12)</td><td>8.1</td><td></td></tr>
</table>

Evidence Category	Evidence Type	Guidelines Default	Range	Max	Count	Points Total	Counted	PMIDs/Notes
	Biochemical Function	0.5	0 - 2					

Experimental Evidence	Function	Protein Interaction	0.5	0 - 2	2			1	
		Expression	0.5	0 - 2		2	1		Merner ND et al. 2008 Apr (PMID:18313022); Christensen AH et al. 2011 Sep (PMID:21214875);
	Functional Alteration	Patient cells	1	0 - 2	2				
		Non-patient cells	0.5	0 - 1					
	Models	Non-human model organism	2	0 - 4	4	1	3	4	Zheng G et al. 2019 Feb (PMID:29980933);
		Cell culture model	1	0 - 2		1	1		Siragam V et al. 2014 Oct 24 (PMID:25343256);
	Rescue	Rescue in human	2	0 - 4					
		Rescue in non-human model organism	2	0 - 4					
		Rescue in cell culture model	1	0 - 2					
		Rescue in patient cells	1	0 - 2					
	**Total Experimental Evidence Points (Maximum 6)**							5	

Assertion criteria	Genetic Evidence (0-12 points)	Experimental Evidence (0-6 points)	Total Points (0-18)	Replication Over Time (Y/N)
Description	Case-level, family segregation, or case-control data that support the gene-disease association	Gene-level experimental evidence that support the gene-disease association	Sum of Genetic & Experimental Evidence	> 2 pubs w/ convincing evidence over time (>3 yrs)
Assigned Points	**8.1**	**5**	**13.1**	**YES**

CALCULATED CLASSIFICATION	LIMITED	1-6
	MODERATE	7-11
	STRONG	12-18
	DEFINITIVE	12-18 AND replication over time

Valid contradictory evidence (Y/N)*	NO	

CALCULATED CLASSIFICATION (DATE)	Definitive	02/04/2020
EXPERT CURATION (DATE)	**Definitive**	10/26/2018
EVIDENCE SUMMARY	The relationship between TMEM43 and arrhythmogenic right ventricular dysplasia (autosomal dominant) was evaluated using the ClinGen Clinical Validity Framework as of July 10th, 2019. Variants in TMEM43 were first reported in humans with this disease as early as 2008 (Merner et al., PMID 18313022). At least 9 variants (mostly missense) have been reported in humans. However, the pathogenicity of most of the variants is unknown. The majority of genetic evidence comes from case-level data and segregation data for one founder variant, p.Ser358Leu, which has been reported in more than 20 families with ARVC and occurred de novo in one individual (Merner et al., 2008, PMID 18313022; Christensen et al. 2011, PMID 21214875; Baskin et al., 2013, PMID 23812740; Hodgkinson et al., 2013, PMID 22725725; Milting et al., 2014, PMID 24598986). This gene-disease relationship is also supported by an animal model, expression stuies, and in vitro assays. In summary, TMEM43 is definitively associated with autosomal dominant arrhythmogenic right ventricular dysplasia . This has been repeatedly demonstrated in both the research and clinical diagnostic settings, and has been upheld over time. This classification was approved by the ClinGen Arrythmogenic Right Ventricular Cardiomyopathy Gene Curation Expert Panel on October 26, 2018 (SOP Version 6).	

TNNC1 - arrhythmogenic right ventricular cardiomyopathy

Gene:	TNNC1 (HGNC:11943)
Disease:	arrhythmogenic right ventricular cardiomyopathy (MONDO_0016587)
Mode of Inheritance:	Autosomal dominant inheritance (HP:0000006)

No Known Disease Relationship

Classification - 09/13/2019

Replication over time: NO

Contradictory Evidence: NO

Expert Panel: Arrhythmogenic Right Ventricular Cardiomyopathy EP

Evidence Summary: There is no evidence that TNN1C is involved in ARVC. This gene has been investigated for pathogenic variants in a cohort of 137 ARVC patients meeting 2010 Task Force criteria for ARVC (29709087). No variants in TNN1C were reported.

Gene Clinical Validity Standard Operating Procedures (SOP) - Version 7

			Evidence Type	Case Information Type	Guidelines Default	Guidelines Range	Guidelines Max	Count	Points Total	Points Counted	PMIDs/Notes
Genetic Evidence	Case-Level Data	Variant Evidence	Autosomal Dominant or X-linked Disorder	Variant is de novo	2	0-3	12				
				Proband with predicted or proven null variant	1.5	0-2	10				
				Proband with other variant type with some evidence of gene impact	0.5	0-1.5	7				
			Autosomal Recessive Disease	Two variants in trans and at least one de novo or a predicted/proven null variant	2	0-3	12				
				Two variants (not predicted/proven null) with some evidence of gene impact in trans	1	0-1.5					
		Segregation Evidence			Summed LOD	Family Count					
				Candidate gene sequencing							
				Exome/genome or all genes sequenced in linkage region							
				Total Summed LOD Score							

	Case-Control Study Type	Case-Control Quality Criteria	Guidelines Points/Study	Guidelines Max	Count	Points	Points Counted	PMIDs/Notes
Case-Control Data	Single Variant Analysis	1. Variant Detection Methodology<br>2. Power<br>3. Bias and confounding<br>4. Statistical Significance	0-6	12				
	Aggregate Variant Analysis		0-6					
	**Total Genetic Evidence Points (Maximum 12)**						0	

	Evidence Category	Evidence Type	Guidelines Default	Guidelines Range	Guidelines Max	Count	Points Total	Points Counted	PMIDs/Notes
Experimental Evidence	Function	Biochemical Function	0.5	0 - 2	2				
		Protein Interaction	0.5	0 - 2					
		Expression	0.5	0 - 2					
	Functional Alteration	Patient cells	1	0 - 2	2				
		Non-patient cells	0.5	0 - 1					
	Models	Non-human model organism	2	0 - 4	4				
		Cell culture model	1	0 - 2					
	Rescue	Rescue in human	2	0 - 4					
		Rescue in non-human model organism	2	0 - 4					
		Rescue in cell culture model	1	0 - 2					
		Rescue in patient cells	1	0 - 2					
	**Total Experimental Evidence Points (Maximum 6)**							0	

Assertion criteria	Genetic Evidence (0-12 points)	Experimental Evidence (0-6 points)	Total Points (0-18)	Replication Over Time (Y/N)
Description	Case-level, family segregation, or case-control data that support the gene-disease association	Gene-level experimental evidence that support the gene-disease association	Sum of Genetic & Experimental Evidence	> 2 pubs w/ convincing evidence over time (>3 yrs)
Assigned Points	0	0	0	NO

CALCULATED CLASSIFICATION		
	LIMITED	1-6
	MODERATE	7-11
	STRONG	12-18
	DEFINITIVE	12-18 AND replication over time

Valid contradictory evidence (Y/N)*	NO	

CALCULATED CLASSIFICATION (DATE)	No Known Disease Relationship	05/08/2020
EXPERT CURATION (DATE)	**No Known Disease Relationship**	09/13/2019
EVIDENCE SUMMARY	There is no evidence that TNN1C is involved in ARVC. This gene has been investigated for pathogenic variants in a cohort of 137 ARVC patients meeting 2010 Task Force criteria for ARVC (29709087). No variants in TNN1C were reported.	

TNNI3 - arrhythmogenic right ventricular cardiomyopathy

Gene:	TNNI3 (HGNC:11947)
Disease:	arrhythmogenic right ventricular cardiomyopathy (MONDO_0016587)
Mode of Inheritance:	Autosomal dominant inheritance (HP:0000006)

No Known Disease Relationship

Classification - 07/16/2019

Replication over time: NO
Contradictory Evidence: NO

Expert Panel: Arrhythmogenic Right Ventricular Cardiomyopathy EP

Evidence Summary: There is only one case reported of a TNNI3 variant in an ARVC patient (30385303) in a Chinese patient. This proband presented in VT of LBBB superior axis morphology and has TWI in leads V1-V4, meeting ARVC TFC criteria. This variant, however is present in higher than expected prevalence in the East Asian population. Otherwise, in other studies (29709087) of 137 patients sequenced for TNNI3, no pathogenic variants were found. Therefore, there is currently no evidence to suggest a role of TNNI3 in pathogenesis of ARVC.
Gene Clinical Validity Standard Operating Procedures (SOP) - Version 7

			Evidence Type	Case Information Type	Guidelines Default	Guidelines Range	Guidelines Max	Count	Points Total	Points Counted	PMIDs/Notes
Genetic Evidence	Case-Level Data	Variant Evidence	Autosomal Dominant or X-linked Disorder	Variant is de novo	2	0-3	12				
				Proband with predicted or proven null variant	1.5	0-2	10				
				Proband with other variant type with some evidence of gene impact	0.5	0-1.5	7				
			Autosomal Recessive Disease	Two variants in trans and at least one de novo or a predicted/proven null variant	2	0-3	12				
				Two variants (not predicted/proven null) with some evidence of gene impact in trans	1	0-1.5					
			Segregation Evidence			Summed LOD		Family Count			
				Candidate gene sequencing							
				Exome/genome or all genes sequenced in linkage region							
				Total Summed LOD Score							

		Case-Control Study Type	Case-Control Quality Criteria	Guidelines Points/Study	Guidelines Max	Count	Points	Points Counted	PMIDs/Notes
Genetic Evidence	Case-Control Data	Single Variant Analysis	1. Variant Detection Methodology<br>2. Power<br>3. Bias and confounding<br>4. Statistical Significance	0-6	12				
		Aggregate Variant Analysis		0-6					
			Total Genetic Evidence Points (Maximum 12)					0	

	Evidence Category	Evidence Type	Guidelines Default	Guidelines Range	Guidelines Max	Count	Points Total	Points Counted	PMIDs/Notes
Experimental Evidence	Function	Biochemical Function	0.5	0 - 2	2				
		Protein Interaction	0.5	0 - 2					
		Expression	0.5	0 - 2					
	Functional Alteration	Patient cells	1	0 - 2	2				
		Non-patient cells	0.5	0 - 1					
	Models	Non-human model organism	2	0 - 4	4				
		Cell culture model	1	0 - 2					
	Rescue	Rescue in human	2	0 - 4					
		Rescue in non-human model organism	2	0 - 4					
		Rescue in cell culture model	1	0 - 2					

	Rescue in patient cells	1	0 - 2				
	**Total Experimental Evidence Points (Maximum 6)**			**0**			

**Assertion criteria**	**Genetic Evidence (0-12 points)**	**Experimental Evidence (0-6 points)**	**Total Points (0-18)**	**Replication Over Time (Y/N)**
**Description**	Case-level, family segregation, or case-control data that support the gene-disease association	Gene-level experimental evidence that support the gene-disease association	Sum of Genetic & Experimental Evidence	> 2 pubs w/ convincing evidence over time (>3 yrs)
**Assigned Points**	**0**	**0**	**0**	**NO**
**CALCULATED CLASSIFICATION**		**LIMITED**	**1-6**	
		**MODERATE**	**7-11**	
		**STRONG**	**12-18**	
		**DEFINITIVE**	**12-18 AND replication over time**	
**Valid contradictory evidence (Y/N)***	NO			
	**CALCULATED CLASSIFICATION (DATE)**	**No Known Disease Relationship**	**05/08/2020**	
	**EXPERT CURATION (DATE)**	**No Known Disease Relationship**	**07/16/2019**	
	**EVIDENCE SUMMARY**	**There is only one case reported of a TNNI3 variant in an ARVC patient (30385303) in a Chinese patient. This proband presented in VT of LBBB superior axis morphology and has TWI in leads V1-V4, meeting ARVC TFC criteria. This variant, however is present in higher than expected prevalence in the East Asian population. Otherwise, in other studies (29709087) of 137 patients sequenced for TNNI3, no pathogenic variants were found. Therefore, there is currently no evidence to suggest a role of TNNI3 in pathogenesis of ARVC.**		

TNNT2 - arrhythmogenic right ventricular cardiomyopathy

Gene:	TNNT2 (HGNC:11949)
Disease:	arrhythmogenic right ventricular cardiomyopathy (MONDO_0016587)
Mode of Inheritance:	Autosomal dominant inheritance (HP:0000006)

No Known Disease Relationship

Classification - 07/16/2019

Replication over time:	NO	Contradictory Evidence:	NO
Expert Panel:	Arrhythmogenic Right Ventricular Cardiomyopathy EP		

Evidence Summary: There is no evidence that TNNT2 is involved in ARVC. This gene has been investigated for pathogenic variants in two populations of gene-elusive ARVC patients: a study of 84 Chinese gene-elusive ARVC patients and a cohort of 137 North American ARVC patients (29709087). No variants in TNNT2 were reported.
Gene Clinical Validity Standard Operating Procedures (SOP) - Version 7

			Evidence Type	Case Information Type	Guidelines Default	Guidelines Range	Guidelines Max	Count	Points Total	Points Counted	PMIDs/Notes
Genetic Evidence	Case-Level Data	Variant Evidence	Autosomal Dominant or X-linked Disorder	Variant is de novo	2	0-3	12				
				Proband with predicted or proven null variant	1.5	0-2	10				
				Proband with other variant type with some evidence of gene impact	0.5	0-1.5	7				
			Autosomal Recessive Disease	Two variants in trans and at least one de novo or a predicted/proven null variant	2	0-3	12				
				Two variants (not predicted/proven null) with some evidence of gene impact in trans	1	0-1.5					
		Segregation Evidence				Summed LOD		Family Count			
				Candidate gene sequencing							
				Exome/genome or all genes sequenced in linkage region							
				Total Summed LOD Score							

		Case-Control Study Type	Case-Control Quality Criteria	Guidelines Points/Study	Guidelines Max	Count	Points	Points Counted	PMIDs/Notes
Genetic Evidence	Case-Control Data	Single Variant Analysis	1. Variant Detection Methodology<br>2. Power<br>3. Bias and confounding<br>4. Statistical Significance	0-6	12				
		Aggregate Variant Analysis		0-6					
		**Total Genetic Evidence Points (Maximum 12)**						0	

	Evidence Category	Evidence Type	Guidelines Default	Guidelines Range	Guidelines Max	Count	Points Total	Points Counted	PMIDs/Notes
Experimental Evidence	Function	Biochemical Function	0.5	0 - 2	2				
		Protein Interaction	0.5	0 - 2					
		Expression	0.5	0 - 2					
	Functional Alteration	Patient cells	1	0 - 2	2				
		Non-patient cells	0.5	0 - 1					
	Models	Non-human model organism	2	0 - 4	4				
		Cell culture model	1	0 - 2					
	Rescue	Rescue in human	2	0 - 4					
		Rescue in non-human model organism	2	0 - 4					
		Rescue in cell culture model	1	0 - 2					
		Rescue in patient cells	1	0 - 2					
	**Total Experimental Evidence Points (Maximum 6)**							0	

Assertion criteria	Genetic Evidence (0-12 points)	Experimental Evidence (0-6 points)	Total Points (0-18)	Replication Over Time (Y/N)
Description	Case-level, family segregation, or case-control data that support the gene-disease association	Gene-level experimental evidence that support the gene-disease association	Sum of Genetic & Experimental Evidence	> 2 pubs w/ convincing evidence over time (>3 yrs)
Assigned Points	0	0	0	NO
CALCULATED CLASSIFICATION		LIMITED	1-6	
		MODERATE	7-11	
		STRONG	12-18	
		DEFINITIVE	12-18 AND replication over time	
Valid contradictory evidence (Y/N)*	NO			
	CALCULATED CLASSIFICATION (DATE)	No Known Disease Relationship	05/08/2020	
	EXPERT CURATION (DATE)	No Known Disease Relationship	07/16/2019	
	EVIDENCE SUMMARY	There is no evidence that TNNT2 is involved in ARVC. This gene has been investigated for pathogenic variants in two populations of gene-elusive ARVC patients: a study of 84 Chinese gene-elusive ARVC patients and a cohort of 137 North American ARVC patients (29709087). No variants in TNNT2 were reported.		

TPM1 - arrhythmogenic right ventricular cardiomyopathy

Gene:	TPM1 (HGNC:12010)
Disease:	arrhythmogenic right ventricular cardiomyopathy (MONDO_0016587)
Mode of Inheritance:	Autosomal dominant inheritance (HP:0000006)

No Known Disease Relationship

Classification - 09/13/2019

Replication over time:	NO	Contradictory Evidence:	NO
Expert Panel:	Arrhythmogenic Right Ventricular Cardiomyopathy EP		

Evidence Summary: There is no evidence that TMP1 is involved in ARVC. This gene has been investigated for pathogenic variants in a cohort of 137 ARVC patients meeting 2010 Task Force criteria for ARVC (29709087). No variants in TMP1 were reported.

Gene Clinical Validity Standard Operating Procedures (SOP) - Version 7

			Evidence Type	Case Information Type	Guidelines Default	Guidelines Range	Guidelines Max	Points Count	Points Total	Points Counted	PMIDs/Notes
Genetic Evidence	Case-Level Data	Variant Evidence	Autosomal Dominant or X-linked Disorder	Variant is de novo	2	0-3	12				
				Proband with predicted or proven null variant	1.5	0-2	10				
				Proband with other variant type with some evidence of gene impact	0.5	0-1.5	7				
			Autosomal Recessive Disease	Two variants in trans and at least one de novo or a predicted/proven null variant	2	0-3	12				
				Two variants (not predicted/proven null) with some evidence of gene impact in trans	1	0-1.5					
			Segregation Evidence		Summed LOD		Family Count				
				Candidate gene sequencing							
				Exome/genome or all genes sequenced in linkage region							
				Total Summed LOD Score							

	Case-Control Study Type	Case-Control Quality Criteria	Guidelines Points/Study	Guidelines Max	Points Count	Points	Points Counted	PMIDs/Notes
Case-Control Data	Single Variant Analysis	1. Variant Detection Methodology<br>2. Power<br>3. Bias and confounding<br>4. Statistical Significance	0-6	12				
	Aggregate Variant Analysis		0-6					
	**Total Genetic Evidence Points (Maximum 12)**						0	

	Evidence Category	Evidence Type	Guidelines Default	Guidelines Range	Guidelines Max	Points Count	Points Total	Points Counted	PMIDs/Notes
Experimental Evidence	Function	Biochemical Function	0.5	0 - 2	2				
		Protein Interaction	0.5	0 - 2					
		Expression	0.5	0 - 2					
	Functional Alteration	Patient cells	1	0 - 2	2				
		Non-patient cells	0.5	0 - 1					
	Models	Non-human model organism	2	0 - 4	4				
		Cell culture model	1	0 - 2					
	Rescue	Rescue in human	2	0 - 4					
		Rescue in non-human model organism	2	0 - 4					
		Rescue in cell culture model	1	0 - 2					
		Rescue in patient cells	1	0 - 2					
	**Total Experimental Evidence Points (Maximum 6)**							0	

Assertion criteria	Genetic Evidence (0-12 points)	Experimental Evidence (0-6 points)	Total Points (0-18)	Replication Over Time (Y/N)
Description	Case-level, family segregation, or case-control data that support the gene-disease association	Gene-level experimental evidence that support the gene-disease association	Sum of Genetic & Experimental Evidence	> 2 pubs w/ convincing evidence over time (>3 yrs)
Assigned Points	0	0	0	NO

CALCULATED CLASSIFICATION	LIMITED	1-6
	MODERATE	7-11
	STRONG	12-18
	DEFINITIVE	12-18 AND replication over time

Valid contradictory evidence (Y/N)*	NO	

CALCULATED CLASSIFICATION (DATE)	No Known Disease Relationship	05/08/2020
EXPERT CURATION (DATE)	No Known Disease Relationship	09/13/2019
EVIDENCE SUMMARY	There is no evidence that TMP1 is involved in ARVC. This gene has been investigated for pathogenic variants in a cohort of 137 ARVC patients meeting 2010 Task Force criteria for ARVC (29709087). No variants in TMP1 were reported.	

TTN - arrhythmogenic right ventricular cardiomyopathy

Gene:	TTN (HGNC:12403)	Limited
Disease:	arrhythmogenic right ventricular cardiomyopathy (MONDO_0016587)	
Mode of Inheritance:	Autosomal dominant inheritance (HP:0000006)	Classification - 08/06/2019

Replication over time: NO   Contradictory Evidence: NO

Expert Panel: Arrhythmogenic Right Ventricular Cardiomyopathy EP

Evidence Summary: TTN: Arrythmogenic Right Ventricular Cardiomyopathy Mode of Inheritance: Autosomal dominant inheritance (HP:0000006) HGNC:12403 MONDO:0016587 Expert Panel: Arrythmogenic Right Ventricular Cardiomyopathy Nine papers were reviewed related to TTN and ARVD (23297410, 23299917, 25157032, 29750433, 24055113, 24082139, 25445213, 29221435, 21810661). In a paper the authors found in 11 of 35 ARVD patients missense variants in TTN. After evaluation of the family members they conclude that TTN missense variants had either a very low penetrance or negligible pathogenicity on the disease (29750433). Gonzalez-Garay et al found a missense variant in an ARVD patient that was also found 19 times in gnomAD (25157032). Only in two papers from the same group potentially relevant information was found. 38 ARVD families were analyzed for the TTN gene because previously analyzed ARVD linked genes (DSC2, DSG2, DSP, and PKP2) were negative. Eight unique TTN nonsynonymous variants were identified in seven unrelated ARVC probands with one proband that was a compound heterozygote having two TTN variants. This is not strange to find because the gene is huge and in reference alleles in gnomAD missense variants are very common. Five of these variants occur relatively often in gnomAD (between 18 and 1134 times) making them less likely as a disease causing variant. One of these variants could be of interest. The variant NM_133378.4 (TTN): c.8687C>T; p.T2896I showed co-segregation (published lod score of 2.41) in 9 (probably) affected with the genotype of whom six of them had ARVD (21810661, 25157032). The variant, present in an Ig10 domain, was further tested in an in vitro functional assay by two independent groups (21810661, 23297410). Both found that the variant introduced an aberrant function. Unfortunately only a very small part of the protein was tested and the TTN protein contains many of these domains therefore masking it difficult to judge if the variant will alter the mutant protein in the patient. Total awarded points = 1.00; Final classification = limited.
Gene Clinical Validity Standard Operating Procedures (SOP) - Version 7

			Evidence Type	Case Information Type	Guidelines Default	Guidelines Range	Guidelines Max	Count	Points Total	Points Counted	PMIDs/Notes
Genetic Evidence	Case-Level Data	Variant Evidence	Autosomal Dominant or X-linked Disorder	Variant is de novo	2	0-3	12				
				Proband with predicted or proven null variant	1.5	0-2	10				
				Proband with other variant type with some evidence of gene impact	0.5	0-1.5	7				
			Autosomal Recessive Disease	Two variants in trans and at least one de novo or a predicted/proven null variant	2	0-3	12				
				Two variants (not predicted/proven null) with some evidence of gene impact in trans	1	0-1.5					
		Segregation Evidence				Summed LOD		Family Count	0.5	0.5	
				Candidate gene sequencing		2.41		1			Taylor M et al. 2011 Aug 23 (PMID:21810661);
				Exome/genome or all genes sequenced in linkage region							
				Total Summed LOD Score		2.41					

	Case-Control Study Type	Case-Control Quality Criteria	Guidelines Points/Study	Guidelines Max	Points Count	Points Counted	PMIDs/Notes
Case-Control Data	Single Variant Analysis	1. Variant Detection Methodology<br>2. Power<br>3. Bias and confounding<br>4. Statistical Significance	0-6	12			
	Aggregate Variant Analysis		0-6				

Total Genetic Evidence Points (Maximum 12) 0.5

Experimental Evidence

		Guidelines				Points		
[redacted]							Counted	PMIDs/Notes
Function	Biochemical Function	0.5	0 - 2	2				
	Protein Interaction	0.5	0 - 2					
	Expression	0.5	0 - 2					
Functional Alteration	Patient cells	1	0 - 2	2			0.5	
	Non-patient cells	0.5	0 - 1		2	0.5		Taylor M et al. 2011 Aug 23 (PMID:21810661); Anderson BR et al. 2013 Feb 22 (PMID:23297410);
Models	Non-human model organism	2	0 - 4	4				
	Cell culture model	1	0 - 2					
Rescue	Rescue in human	2	0 - 4					
	Rescue in non-human model organism	2	0 - 4					
	Rescue in cell culture model	1	0 - 2					
	Rescue in patient cells	1	0 - 2					

Total Experimental Evidence Points (Maximum 6) 0.5

Assertion criteria	Genetic Evidence (0-12 points)	Experimental Evidence (0-6 points)	Total Points (0-18)	Replication Over Time (Y/N)
Description	Case-level, family segregation, or case-control data that support the gene-disease association	Gene-level experimental evidence that support the gene-disease association	Sum of Genetic & Experimental Evidence	> 2 pubs w/ convincing evidence over time (>3 yrs)
Assigned Points	0.5	0.5	1	NO

CALCULATED CLASSIFICATION	LIMITED	1-6
	MODERATE	7-11
	STRONG	12-18
	DEFINITIVE	12-18 AND replication over time

Valid contradictory evidence (Y/N)*	NO	

CALCULATED CLASSIFICATION (DATE)	Limited	02/04/2020
EXPERT CURATION (DATE)	**Limited**	08/06/2019

EVIDENCE SUMMARY

TTN: Arrythmogenic Right Ventricular Cardiomyopathy Mode of Inheritance: Autosomal dominant inheritance (HP:0000006) HGNC:12403 MONDO:0016587 Expert Panel: Arrythmogenic Right Ventricular Cardiomyopathy Nine papers were reviewed related to TTN and ARVD (23297410, 23299917, 25157032, 29750433, 24055113, 24082139, 25445213, 29221435, 21810661). In a paper the authors found in 11 of 35 ARVD patients missense variants in TTN. After evaluation of the family members they conclude that TTN missense variants had either a very low penetrance or negligible pathogenicity on the disease (29750433). Gonzalez-Garay et al found a missense variant in an ARVD patient that was also found 19 times in gnomAD (25157032). Only in two papers from the same group potentially relevant information was found. 38 ARVD families were analyzed for the TTN gene because previously analyzed ARVD linked genes (DSC2, DSG2, DSP, and PKP2) were negative. Eight unique TTN nonsynonymous variants were identified in seven unrelated ARVC probands with one proband that was a compound heterozygote having two TTN variants. This is not strange to find because the gene is huge and in reference alleles in gnomAD missense variants are very common. Five of these variants occur relatively often in gnomAD (between 18 and 1134 times) making them less likely as a disease causing variant. One of these variants could be of interest. The variant NM_133378.4 (TTN): c.8687C>T; p.T2896I showed co-segregation (published lod score of 2.41) in 9 (probably) affected with the genotype of whom six of them had ARVD (21810661, 25157032). The variant, present in an Ig10 domain, was further tested in an in vitro functional assay by two independent groups (21810661, 23297410). Both found that the variant introduced an aberrant function. Unfortunately only a very small part of the protein was tested and the TTN protein contains many of these domains therefore masking it difficult to judge if the variant will alter the mutant protein in the patient. Total awarded points = 1.00; Final classification = limited.
