# Supplementary material for: International Evidence Based Reappraisal of Genes Associated With Arrhythmogenic Right Ventricular Cardiomyopathy Using the Clinical Genome Resource Framework
Source: Circ Genom Precis Med. 2021 Apr 8;14(3):e003273. doi: 10.1161/CIRCGEN.120.003273 (PMC8205996; doi:10.1161/CIRCGEN.120.003273)
Supplement: Supplementary file 2 [file hcg-14-e003273-s002.pdf]

DOI: 10.1161/CIRCGEN.120.003273

An International Evidence Based Reappraisal of Genes Associated with Arrhythmogenic Right Ventricular Cardiomyopathy (ARVC) using the ClinGen Framework

Running title: *James et al.; ARVC ClinGen gene curation*

Cynthia A. James, PhD, CGC[1]; Jan D.H. Jongbloed, PhD[2]; Ray E. Hershberger, MD[3,4]; Ana Morales, MS, CGC[4]; Daniel P. Judge, MD[5]; Petros Syrris, PhD[6]; Kalliopi Pilichou, PhD[7]; Argelia Medeiros Domingo, MD, PhD[8]; Brittney Murray, MS, CGC[1]; Julia Cadrin-Tourigny, MD[9]; Ronald Lekanne Deprez, PhD[10]; Rudy Celeghin, PhD[7]; Alexandros Protonotarios, MD[6]; Babken Asatryan MD, PhD[8]; Emily Brown, MS,CGC[1]; Elizabeth Jordan, MS, LCGC[3]; Jennifer McGlaughon, PhD[11]; Courtney Thaxton, PhD[11]; C. Lisa Kurtz, PhD[11]; J. Peter van Tintelen, MD, PhD[10,12]

[1]Dept of Medicine, Division of Cardiology, Johns Hopkins Hospital, Baltimore, MD; [2]Dept of Genetics, Univ of Groningen, Univ Medical Center Groningen, Groningen, the Netherlands; [3]Dept of Internal Medicine, Division of Cardiovascular Medicine, [4]Dept of Internal Medicine, Division of Human Genetics, Ohio State Univ, Columbus, OH; [5]Division of Cardiology, Dept of Medicine Medical Univ of South Carolina, Charleston, SC; [6]Centre for Heart Muscle Disease, Inst of Cardiovascular Science, Univ College London, London, UK; [7]Univ of Padua, Dept of Cardiac-Thoracic-Vascular Sciences & Public Health, Padua, Italy; [8]Dept for Cardiology, Inselspital, Bern Univ Hospital, Univ of Bern, Bern, Switzerland; [9]Cardiovascular Genetics Ctr, Montreal Heart Inst, Université de Montréal, Montréal, Canada; [10]Dept of Clinical Genetics, Amsterdam UMC, Univ of Amsterdam, Amsterdam, the Netherlands; [11]Dept of Genetics, Univ of North Carolina, Chapel Hill, NC; [12]Dept of Genetics, Univ of Utrecht, Univ Medical Ctr Utrecht, Utrecht, the Netherlands

American Heart Association.
Circulation: Genomic and Precision Medicine

Correspondence:

Cynthia A. James, PhD
Division of Cardiology, Dept of Medicine
Carnegie 568D
Johns Hopkins School of Medicine
600 N. Wolfe St.
Baltimore, MD 21287-0409
Phone: 443-287-5985, Fax: 410-502-9148
Email: cjames7@jhmi.edu

J. Peter van Tintelen, MD, PhD
Dept of Genetics
Room number KC.04.052.; P.O. Box 85090
University Medical Center, Utrecht
3508 AB Utrecht
the Netherlands
Phone: +31 88 75 538 21
Email: J.P.vanTintelen-3@umcutrecht.nl

Journal Subject Terms: Genetics; Arrhythmias; Sudden Cardiac Death; Cardiomyopathy; Electrophysiology

This article is published in its accepted form; it has not been copyedited and has not appeared in an issue of the journal. Preparation for inclusion in an issue of *Circulation: Genomic and Precision Medicine* involves copyediting, typesetting, proofreading, and author review, which may lead to differences between this accepted version of the manuscript and the final published version.

Abstracts:

Background - Arrhythmogenic right ventricular cardiomyopathy (ARVC) is an inherited disease characterized by ventricular arrhythmias and progressive ventricular dysfunction. Genetic testing is recommended and a pathogenic variant in an ARVC-associated gene is a major criterion for diagnosis according to the 2010 Task Force Criteria (TFC). As incorrect attribution of a gene to ARVC can contribute to misdiagnosis, we assembled an international multidisciplinary ARVC ClinGen Gene Curation Expert Panel to reappraise all reported ARVC genes.

Methods - Following a comprehensive literature search, six two-member teams conducted blinded independent curation of reported ARVC genes using the semi-quantitative ClinGen framework.

Results - Of 26 reported ARVC genes, only six (*PKP2*, *DSP*, *DSG2*, *DSC2*, *JUP*, *TMEM43*) had strong evidence and were classified as definitive for ARVC causation. There was moderate evidence for two genes, *DES* and *PLN*. The remaining 18 genes had limited or no evidence. *RYR2* was refuted as an ARVC gene since clinical data and model systems exhibited a catecholaminergic polymorphic ventricular tachycardia (CPVT) phenotype. In ClinVar, only 5 pathogenic / likely pathogenic (P/LP) variants (1.1%) in limited evidence genes had been reported in ARVC cases in contrast to 450 desmosome gene variants (97.4%).

Conclusions - Using the ClinGen approach to gene-disease curation, only eight genes, (*PKP2*, *DSP*, *DSG2*, *DSC2*, *JUP*, *TMEM43*, *PLN*, *DES*) had definitive or moderate evidence for ARVC and these genes accounted for nearly all P/LP ARVC variants in ClinVar. Therefore, only P/LP variants in these eight genes should yield a major criterion for ARVC diagnosis. P/LP variants identified in other genes in a patient should prompt further phenotyping as variants in many of these genes are associated with other cardiovascular conditions.

Key words: arrhythmogenic right ventricular cardiomyopathy; arrhythmogenic right ventricular dysplasia/cardiomyopathy; desmosome; genetic testing; ClinGen

This article is published in its accepted form; it has not been copyedited and has not appeared in an issue of the journal. Preparation for inclusion in an issue of *Circulation: Genomic and Precision Medicine* involves copyediting, typesetting, proofreading, and author review, which may lead to differences between this accepted version of the manuscript and the final published version.

DOI: 10.1161/CIRCGEN.120.003273

Nonstandard Abbreviations and Acronyms

ARVC	arrhythmogenic right ventricular cardiomyopathy
CPVT	catecholaminergic polymorphic ventricular tachycardia
DCM	dilated cardiomyopathy
GCEP	gene curation expert panel
GCI	gene curation interface
MAF	minor allele frequency
P/LP	pathogenic or likely pathogenic
TFC	2010 Task Force Criteria

American Heart Association.

Circulation: Genomic and Precision Medicine

Introduction

Arrhythmogenic right ventricular cardiomyopathy (ARVC) is an inherited cardiomyopathy characterized by fibro-fatty myocardial replacement, frequent ventricular arrhythmias, and slowly progressive ventricular dysfunction. Patients typically present between their second and fifth decades with symptoms associated with ventricular arrhythmias[1]. Sudden cardiac death is a common presentation, occurring in up to half of probands.

ARVC clusters in families and its pattern of inheritance is generally autosomal dominant with age-related, reduced penetrance[2]. The discovery that Naxos disease, a rare cardiocutaneous autosomal recessive form of ARVC, was caused by pathogenic variants in *JUP*-encoded plakoglobin[3] prompted rapid identification of pathogenic variants in other desmosomal genes (*PKP2*, *DSP*, *DSG2*, *DSC2*) in ARVC populations. In contemporary ARVC cohorts meeting

This article is published in its accepted form; it has not been copyedited and has not appeared in an issue of the journal. Preparation for inclusion in an issue of *Circulation: Genomic and Precision Medicine* involves copyediting, typesetting, proofreading, and author review, which may lead to differences between this accepted version of the manuscript and the final published version.

2010 Task Force Criteria (TFC), up to two-thirds of cases have pathogenic / likely pathogenic (P/LP) desmosomal variants[4,5].

Exponential growth in sequencing capacity led investigators to sequence ever-growing lists of candidate genes and to undertake exome and genome sequencing in attempts to identify the genetic basis of ARVC in gene-elusive patients. These studies identified variants of interest in numerous genes which were then suggested to be ARVC-causative. However, some reports were based on an incomplete understanding of the extent of rare variation in the human genome. Older studies used dated diagnostic criteria for inclusion. Finally, isolated gene-elusive ARVC may be oligogenic[2], calling the assumptions underlying some gene identification studies into question. Despite these limitations, newly-reported ARVC genes have been promptly added to diagnostic ARVC sequencing panels which now generally range from 11-46 genes. Genetic testing is recommended for patients with ARVC to confirm the diagnosis, inform management, and enable cascade genetic testing[6]

Circulation: Genomic and Precision Medicine

The NIH-funded Clinical Genome Resource (ClinGen) created a standardized evidence-based framework to systematically assess gene-disease relationships[7]. Recently, evaluation of genes associated with hypertrophic cardiomyopathy[8], long QT syndrome[9], and Brugada syndrome[10] called into question causality of many disease genes. Similar weaknesses in conventional understanding of the genetic architecture for ARVC seemed likely. ARVC can be difficult to diagnose, particularly when phenotypic expression is mild or when the patient has biventricular disease raising the possibility that gene:diseases associations may have been erroneously derived from participants with other cardiovascular diseases. The TFC were updated in 2010 and older manuscripts relied on less sensitive and specific 1994 criteria[5] making phenotyping in older publications potentially problematic.

This article is published in its accepted form; it has not been copyedited and has not appeared in an issue of the journal. Preparation for inclusion in an issue of *Circulation: Genomic and Precision Medicine* involves copyediting, typesetting, proofreading, and author review, which may lead to differences between this accepted version of the manuscript and the final published version.

Since identification of a P/LP variant constitutes a major criterion in the TFC[5] accurate understanding of genetic architecture has direct implications for ARVC diagnosis and management. Incorrect ARVC gene:disease associations may result in: 1) a patient's phenotype incorrectly attributed to a variant leading to potential over-diagnosis and incorrect cascade genetic testing or 2) a variant associated with a different disease incorrectly attributed to ARVC missing the opportunity for correct genotype-specific management of a family. Therefore, an international multidisciplinary ARVC ClinGen Gene Curation Expert Panel (GCEP) (Supplementary Table I; https://clinicalgenome.org/affiliation/40003/) with expertise in ARVC research, genetics, and clinical care was assembled to formally reappraise all previously-reported ARVC genes using the ClinGen Gene-Disease Clinical Validity Framework. To enhance scientific rigor we used a dual, blinded, independent curation approach (Figure 1). In this effort, we defined ARVC by fulfillment of the 2010 TFC(5). While arrhythmogenic cardiomyopathy (ACM) has been suggested as a concept by several groups of authors[6,11,12], at present there is considerable variability in the breadth of phenotypes covered by the term "ACM" and no standard agreed-upon diagnostic criteria that could be applied for gene curation. Here we report our results.

Materials and Methods

The data that support the findings of this study are available from the corresponding authors upon reasonable request. The study was approved by the Johns Hopkins Medicine institutional review board (data for variant frequency cutoffs) and subjects gave informed consent. The full methods for this study are available as supplemental material (Supplemental Methods).

Circulation: Genomic and Precision Medicine

American Heart Association.

This article is published in its accepted form; it has not been copyedited and has not appeared in an issue of the journal. Preparation for inclusion in an issue of *Circulation: Genomic and Precision Medicine* involves copyediting, typesetting, proofreading, and author review, which may lead to differences between this accepted version of the manuscript and the final published version.

DOI: 10.1161/CIRCGEN.120.003273

Results

Overview

PubMed/OMIM searches resulted in 26 genes reported to cause human ARVC: *ACTC1*, *CDH2*, *CTNNA3*, *LDB3*, *DES*, *DSC2*, *DSG2*, *DSP*, *JUP*, *LMNA*, *MYBPC3*, *MYH7*, *MYL2*, *MYL3*, *PKP2*, *PLN*, *RYR2*, *SCN5A*, *TGFB3*, *TJP1*, *TMEM43*, *TNNI3*, *TNNC1*, *TNNT2*, *TPM1*, and *TTN* (Table 1). As shown in Figure 2, based on initial scoring, six genes (*PKP2*, *DSP*, *DSC2*, *DSG2*, *JUP*, *TMEM43*) had strong evidence (12-18 points) and were judged to be definitive for ARVC causation as each had replication across ARVC cohorts. There was moderate evidence for two genes, *DES* (9.5 points) and *PLN* (11 points). The remaining genes had only limited or no evidence for ARVC causation (0-6 points). Curation team scores were highly concordant. For every gene both curation teams arrived at the same preliminary classification based on points achieved (Supplementary Table II).

Circulation: Genomic and Precision Medicine

Table 2 summarizes the evidence for genes designated to have definitive or moderate evidence for ARVC causation by the GCEP. The evidence used to arrive at these final classifications was predominantly derived from clinical genetic studies. Supplementary Table III shows scoring for genes with limited or no evidence for ARVC. Granular scores for each subcategory of genetic and experimental evidence can be found for each gene in Supplementary Table IV. The most up-to-date curation data for each gene can be accessed at https://clinicalgenome.org/.

As shown in Table 2, the GCEP classified each gene with a score in the strong range (12-18 points) as having definitive evidence for ARVC. The genes encoding the cardiac desmosome (*PKP2*, *DSP*, *DSG2*, *DSC2*, *JUP*) have been consistently identified across multiple ARVC cohorts. *TMEM43* was initially identified due to a founder variant segregating in a well-

This article is published in its accepted form; it has not been copyedited and has not appeared in an issue of the journal. Preparation for inclusion in an issue of *Circulation: Genomic and Precision Medicine* involves copyediting, typesetting, proofreading, and author review, which may lead to differences between this accepted version of the manuscript and the final published version.

characterized ARVC population in Newfoundland, and subsequently in the UK, Denmark, and Germany and recently on a different haplotype in Spain[13-15]. The GCEP confirmed the moderate-evidence classification of *PLN* which was likewise initially associated with ARVC due to a segregating founder variant[16]. A moderate-evidence classification was also confirmed for *DES*. While not typically considered an ARVC gene, it is frequently associated with a myopathy[17] in which arrhythmogenic cardiomyopathy (myofibrillar myopathy-1, desminopathy) is a presenting feature and patients can meet TFC[18].

The GCEP concluded the remaining 18 genes - more than two thirds of the genes analyzed - did not have convincing evidence for ARVC causality. These fell into two categories: 1) genes – often newly published - with rare variants detected in small families with a clear ARVC phenotype but for which data was yet limited, and 2) genes known to be associated with other cardiomyopathies or arrhythmia syndromes where evidence for ARVC causality was relatively scant. Notably, the panel refuted *RYR2* as an ARVC gene, finding P/LP variants in *RYR2* typically cause catecholaminergic polymorphic ventricular tachycardia (CPVT) not ARVC. All the literature asserting ARVC causality was judged to be incorrect and based on incomplete phenotyping, wrong interpretation of clinical data (personal communication G. Thiene, C. Basso), use of dated diagnostic criteria, and designation of relatively common *RYR2* variants as pathogenic.

American Heart Association.
Circulation: Genomic and Precision Medicine

Genes with definitive evidence for ARVC causality

Cardiac desmosomes are specialized structures composed of proteins (cadherins, armadillo proteins, plakins) responsible for cardiomyocyte adhesion. ARVC is classically considered a “disease of the desmosome” and the desmosomal genes rapidly achieved sufficient evidence for a strong designation with replication across cohorts making the gene:disease relationship

This article is published in its accepted form; it has not been copyedited and has not appeared in an issue of the journal. Preparation for inclusion in an issue of *Circulation: Genomic and Precision Medicine* involves copyediting, typesetting, proofreading, and author review, which may lead to differences between this accepted version of the manuscript and the final published version.

definitive. There were several nuances. First, ARVC can be accompanied by skin and hair features. This has long been recognized in families with *JUP*-associated Naxos disease and *DSP*-associated Carvajal syndrome, both of which have autosomal recessive inheritance. Several authors have reported skin and hair findings in patients with heterozygous *DSP* variants which can be subtle[19]. Importantly, there is evidence across studies for a relatively high prevalence of patients with multiple desmosomal P/LP variants beyond Naxos disease and Carvajal syndrome. Pedigrees segregating multiple *DSC2* and *DSG2* variants have been reported and inheritance more consistent with autosomal recessive than autosomal dominant inheritance has been recognized in specific populations[20,21].

The *TMEM43* (transmembrane protein 43) gene encodes a nuclear membrane protein. One heterozygous pathogenic variant (NM_024334.3(TMEM43):c.1073C>T; p.Ser358Leu) was identified as a founder mutation in a large number of patients and families from Newfoundland, Denmark and Germany and has also been identified in other populations[15,22]. It is associated with a highly penetrant and arrhythmogenic subtype of ARVC in which biventricular involvement can often be appreciated. Evidence of pathogenicity of other *TMEM43* variants remains limited.

Circulation: Genomic and Precision Medicine

Moderate evidence genes

DES and *PLN*, had moderate evidence for ARVC causality. *DES* was initially proposed as an ARVC gene based on data from 27 Dutch individuals in five families segregating a rare missense variant (NM_001927.4(DES):c.38C>T; p.Ser13Phe)[23]. Cases had right ventricular involvement consistent with ARVC but also conduction disease which is atypical for ARVC. Additional families carrying *DES* LP/P variants with a clinical ARVC diagnosis as well as families with left-predominant disease have been described[18,24,25]. Experimental evidence including expression systems integrating variants found in these families showed phenotypic alterations

This article is published in its accepted form; it has not been copyedited and has not appeared in an issue of the journal. Preparation for inclusion in an issue of *Circulation: Genomic and Precision Medicine* involves copyediting, typesetting, proofreading, and author review, which may lead to differences between this accepted version of the manuscript and the final published version.

consistent with histological examinations of skeletal and cardiac muscle of ARVC cases[18] and disruption of cellular adhesion[26]. Nonetheless, *DES* variants associated with ARVC appear to be very rare and have not been observed in some large ARVC cohorts.

The *PLN* p.Arg14del variant (NM_002667.5(PLN):c.37_39AGA[1]) was first identified in ARVC in a cohort of 12/97 patients fulfilling TFC[27]. Histology showed the typical fibrofatty replacement and interstitial fibrosis yet compared with desmosomal gene positive patients, *PLN* p. Arg14del showed significantly more severe fibrotic changes in the left ventricle, underscoring its biventricular character[28].

Limited / no human evidence genes

Ten genes had limited evidence for ARVC causality (1-6 points): *SCN5A*, *LMNA*, *CDH2*, *CTNNA3*, *TGFB3*, *TTN*, *TJP1*, *MYH7*, *MYBPC3*, *MYL3*. In comparison to the wealth of literature linking desmosomal genes with ARVC, evidence for these gene:disease relationships had been generated by relatively few research groups. For some of these genes assertions of ARVC-causality are relatively new and further data from larger cohorts might lead to an upgraded level of evidence in the future. For others, the assertion of ARVC causality was published some time ago, leading the panel to give additional weight to the failure to confirm the observation across ARVC cohorts. Our review strongly suggested that none of these genes account for a substantial fraction of ARVC patients.

Genes with variants identified in classic ARVC families but yet-limited evidence

CDH2, *CTNNA3*, and *TJP1* showed evidence for a classic ARVC phenotype and segregation in several relatively small families but with human data as yet limited. *CDH2* and *CTNNA3* encode the area composita proteins cadherin-2 and alpha T catenin respectively. *TJP1* encodes a

American Heart Association.

Circulation: Genomic and Precision Medicine

This article is published in its accepted form; it has not been copyedited and has not appeared in an issue of the journal. Preparation for inclusion in an issue of *Circulation: Genomic and Precision Medicine* involves copyediting, typesetting, proofreading, and author review, which may lead to differences between this accepted version of the manuscript and the final published version.

scaffolding protein, tight junction protein-1, which localizes to the intercalated discs in cardiomyocytes.

Evidence for *CDH2* included identification of two LP rare missense variants in three probands meeting TFC[29,30]. One variant (NM_001792.4(CDH2): c.1219G>A; p.Asp407Asn) was identified in both a South African and a Norwegian family. Another (NM_001792.4(CDH2): c.686A>C; p.Gln229Pro) segregated among five affected family members. A murine knockout model showed disrupted desmosomes, cardiomyopathy, ventricular tachycardia, and sudden death, suggestive of a phenotype compatible with ARVC.

Similarly, for *CTNNA3*, two variants were reported in two ARVC probands: one likely *de novo* missense variant absent from gnomAD (NM_013266.3(CTNNA3): c.281T>A; p.Val94Asp) and one in-frame deletion (NM_013266.3(CTNNA3): c.2296_2298del; p.Leu766del) with limited segregation[31]. A germline knockout mouse showed altered PKP2 distribution without affecting other junctional components of the area composita. These mice had progressive dilated cardiomyopathy (DCM), and the GCEP judged the phenotype not completely convincing for ARVC. Furthermore, no *CTNNA3* LP/P variants were reported in two series of gene elusive ARVC patients.

American Heart Association.
Circulation: Genomic and Precision Medicine

Finally, two probands with ARVC (as well as two probands with DCM) had variants in *TJP1*[32]. Modest segregation data allowed this to be counted as limited human evidence.

Genes associated with other cardiomyopathies / arrhythmia syndromes

The remaining genes curated were each strongly associated with other cardiomyopathies or arrhythmia syndromes. The GCEP concluded each had limited or no evidence for ARVC causality. The gene with the most evidence (6 points) was *SCN5A* which encodes the Nav 1.5 sodium channel, and previously curated as definitive for both Brugada syndrome and long QT

This article is published in its accepted form; it has not been copyedited and has not appeared in an issue of the journal. Preparation for inclusion in an issue of *Circulation: Genomic and Precision Medicine* involves copyediting, typesetting, proofreading, and author review, which may lead to differences between this accepted version of the manuscript and the final published version.

syndrome[9,10]. The most robust evidence for *SCN5A* as an ARVC gene comes from identification of a variant (NM_198056.2(SCN5A): c.5693G>A; p.Arg1898His) in a gene-elusive ARVC patient via exome sequencing followed by derivation of an induced pluripotent stem cell-derived cardiomyocyte model[33]. This model showed a one-third reduction in peak sodium current and reduced abundance of both SCN5A and CDH2 clusters at the intercalated disk which normalized in a CRISPR/Cas9 corrected line. The authors subsequently identified five *SCN5A* variants among 281 ARVC probands. One variant was excluded for being too common and two were found in probands who also had pathogenic desmosomal variants. One proband had an in-frame deletion (NM_198056.2(SCN5A): c.2184-2186del; p.Leu729del) which segregated with the phenotype, but most family members did not fulfill definite TFC.

American Heart Association.

Potentially pathogenic variants in *LMNA* have been published in several ARVC cohorts[34,35]. While evidence was sufficient to merit a limited-evidence classification, the GCEP noted that while most probands did meet TFC, many of their affected family members did not. The phenotypes observed overlapped with DCM and were characterized by prominent conduction system abnormalities and atrial arrhythmias.

Circulation: Genomic and Precision Medicine

Eleven papers were reviewed to assess the relationship of *TGFB3* with ARVC, the majority from the same research group. *TGFB3* emerged as a candidate gene based on linkage to 14q23-q24 in several Italian families. Sequencing found variants in the 3' untranslated region in one family and a second regulatory noncoding variant in an unrelated family[36]. These variants were both associated with increased activity compared to wildtype in an expression assay. However, two of the initial families with significant linkage to the candidate region had no P/LP variants in the *TGFB3* coding sequences, UTRs, and promoter regions. No definitively pathogenic variants have been subsequently reported in ARVC probands. Nowadays *TGFB3* is

This article is published in its accepted form; it has not been copyedited and has not appeared in an issue of the journal. Preparation for inclusion in an issue of *Circulation: Genomic and Precision Medicine* involves copyediting, typesetting, proofreading, and author review, which may lead to differences between this accepted version of the manuscript and the final published version.

believed to underlie Loeys-Dietz syndrome type 5, a connective tissue disease phenotype with features of Marfan syndrome, including aortic abnormalities[37]. The GCEP concluded that while *TGFB3* merited a limited-evidence classification for ARVC, a variant detected in *TGFB3* should be treated with great caution, and is unlikely the cause of a patient's ARVC.

TTN, encoding titin, is a frequent cause of familial DCM. Nine papers were evaluated for the role of *TTN* variants in ARVC causation. Many reported missense variants which were relatively common in gnomAD. Furthermore, in study reporting 11/35 ARVC probands with *TTN* missense variants, relatives carrying the variant had no evidence of disease leading them to conclude these *TTN* variants had very low penetrance or negligible pathogenicity[38]. One paper did describe a rare missense variant NM_133378.4 (TTN): c.8678C>T; p.Thr2896Ile that segregated among nine family members – six of whom met TFC[39]. An *in vitro* functional assay by two independent groups found that the variant introduced aberrant function. Further evidence has shown that *TTN*-associated DCM is not particularly associated with an arrhythmogenic phenotype[40]. The GCEP thus concluded there was very limited evidence for *TTN* as ARVC-causative.

Circulation: Genomic and Precision Medicine

The sarcomere genes have been considered by several research groups as a potential cause of ARVC[41]. *MYH7*, *MYBPC3*, and *MYL3* were scored as having very limited evidence. The other sarcomere genes had no evidence. These genes are well-established as causative for hypertrophic cardiomyopathy[8].

Refuted and disputed genes - *RYR2* is not an ARVC gene

The GCEP refuted the association of *RYR2* with ARVC. A thorough review including 57 papers showed that the assertion of ARVC causality was initially derived from three publications from the same research group who first established linkage to chromosome 1q42-43, and subsequently

This article is published in its accepted form; it has not been copyedited and has not appeared in an issue of the journal. Preparation for inclusion in an issue of *Circulation: Genomic and Precision Medicine* involves copyediting, typesetting, proofreading, and author review, which may lead to differences between this accepted version of the manuscript and the final published version.

to *RYR2* in families with a phenotype called ARVD2 described as CPVT with fibrofatty replacement of the right ventricle[42]. The clinical features described in these manuscripts reflect CPVT rather than ARVC with cases not meeting TFC. This was confirmed by collaborators from the original research group (C. Basso, G. Thiene personal communication). A mouse model with one of the variants also showed a CPVT–like phenotype with no evidence of fibrofatty infiltration or structural alterations characteristic of ARVC[43]. In papers reporting *RYR2* missense variants in possible ARVC probands the minor allele frequency (MAF) was often too high, cases did not have a clear ARVC diagnosis, segregation information was often not informative, and in several cases CPVT was said to also be present in the family. Rare cases of *RYR2* deletions associated with DCM with CPVT-like arrhythmias have been described[44], but none associated with ARVC. Taken together the evidence is convincing that pathogenic variants in *RYR2* do not cause ARVC, rather they cause CPVT.

Circulation: Genomic and Precision Medicine

LDB3 was disputed as a cause for ARVC. The only variant reported in an ARVC family had a MAF higher than the cutoff established, particularly in the relevant ethnic population (Europeans).

Prevalence of variants reported in ClinVar for each gene

Figure 3 shows the distribution of variants reported to ClinVar associated with ARVC (panel A) and the proportion of P/LP variants found in the genes with definitive or moderate evidence for ARVC in comparison to the other genes (panel B). As can be appreciated, ARVC-associated P/LP variants were nearly exclusively reported in the desmosomal genes (450/462, 97.4%) with the established founder variants in *PLN* and *TMEM43* also reported. Notably, only 5 P/LP variants (1.1%) were reported in genes with limited evidence including one variant in *CTNNA3*, three in *LMNA*, and one in *TGFB3*.

This article is published in its accepted form; it has not been copyedited and has not appeared in an issue of the journal. Preparation for inclusion in an issue of *Circulation: Genomic and Precision Medicine* involves copyediting, typesetting, proofreading, and author review, which may lead to differences between this accepted version of the manuscript and the final published version.

Discussion

The data presented here, derived from a rigorous, international evaluation of 26 genes published as ARVC-causing using the ClinGen framework, confirm that ARVC is primarily a disease of the cardiac desmosome, with *PKP2*, *DSP*, *DSC2*, *DSG2*, and *JUP*, definitively associated with ARVC and these genes accounting for nearly all reported ARVC-associated P/LP variants. *PLN* and *TMEM43* contribute to disease pathogenesis, particularly in geographic regions with well-characterized founder variants. This study also demonstrates that the majority of published ARVC genes had only limited (N=10) or no (N=8) evidence, and contribute little to the classic ARVC phenotype. While there has been extensive discussion of the genetic heterogeneity of ARVC and overlap syndromes, P/LP variants in genes with strong / definitive evidence for another cardiovascular disease do not substantially contribute to ARVC causation. In particular, this analysis disqualified *RYR2* as an ARVC gene, finding cases and model systems instead had CPVT. Taken together, these findings call into question the extent of genetic heterogeneity truly contributing to classic ARVC as defined by the TFC.

Circulation: Genomic and Precision Medicine

This reappraisal of ARVC genes is strengthened by our methodological approach that included an extensive literature review, dual, blinded, independent curation, and final adjudication of evidence by an international multidisciplinary panel with substantial experience with ARVC. The semi-quantitative ClinGen framework for evidence classification was effective. The independent curation teams had a high degree of uniformity in applying the framework with 100% concordance of preliminary classification of the level of evidence and no disagreement between the curation teams’ conclusions and the final opinion of the other GCEP members in arriving at the final level of evidence.

This article is published in its accepted form; it has not been copyedited and has not appeared in an issue of the journal. Preparation for inclusion in an issue of *Circulation: Genomic and Precision Medicine* involves copyediting, typesetting, proofreading, and author review, which may lead to differences between this accepted version of the manuscript and the final published version.

DOI: 10.1161/CIRCGEN.120.003273

Eighteen of the 26 published ARVC genes had either limited or no evidence for ARVC causation. Excluded genes fell into two categories: 1) recently published genes with rare variants detected in small families with clear ARVC but for which data was limited and 2) genes known to be associated with other cardiomyopathies or arrhythmia syndromes where a thorough literature review showed limited or no evidence for ARVC causality. For the genes in the first category (*CDH2*, *CTNNA3*, *TJP1*) multicenter studies, some currently underway, will address whether the identification of segregating P/LP variants in ARVC families will be replicated and if so in what proportion of gene elusive ARVC cases. These data could lead to the level of evidence for these genes being upgraded in the future.

This misattribution of the latter group of genes to ARVC illustrates the well-known challenges of ascertaining, and then attributing, clinical data to a specific cardiac disease. This challenge, particularly prior to 2010 when the ARVC diagnostic criteria were refined, was the primary reason for the erroneous assertion that *RYR2* caused ARVC, with our review revealing affected individuals in published pedigrees segregating *RYR2* variants had clinical characteristics consistent with CPVT rather than ARVC. Diagnostic challenges also emerged in papers that described associations of *LMNA*, *TTN*, *SCN5A*, and even the moderate-evidence gene *DES* with ARVC. In most, while several cases met 2010 TFC, others did not and the pedigrees often included clinical features not typically seen in ARVC.

Older genetic/genomic methodologies also contributed to incorrect assertions of ARVC causality. Some papers identified variants of interest as likely pathogenic based on small control cohorts. Reassessment revealed the MAF of quite a few variants was too high given current understanding of the frequency of rare variants in the general population.

American Heart Association.
Circulation: Genomic and Precision Medicine

This article is published in its accepted form; it has not been copyedited and has not appeared in an issue of the journal. Preparation for inclusion in an issue of *Circulation: Genomic and Precision Medicine* involves copyediting, typesetting, proofreading, and author review, which may lead to differences between this accepted version of the manuscript and the final published version.

Clinical implications

Genetic testing is recommended for ARVC patients and genetic test results are part of the 2010 TFC[5,6]. Optimal genetic testing requires both wise genetic test selection and accurate interpretation of results. By defining the genetic architecture of ARVC, this study informs both.

Interpretation and utilization of genetic test results for diagnosis and cascade testing

A pathogenic variant "categorized as associated or probably associated with ARVC" constitutes a major ARVC diagnostic criterion[5]. Based on our results, we recommend that only P/LP variants in genes with definitive and moderate evidence for ARVC causation (*PKP2*, *DSP*, *DSC2*, *DSG2*, *JUP*, *TMEM43*, *PLN*, *DES*) should yield a major criterion for ARVC diagnosis.

We found that genes with strong or definitive evidence for other cardiovascular diseases had at-most moderate (*DES*) and usually limited or no evidence for ARVC causation. This suggests skepticism is warranted when a P/LP variant in one of these genes is identified in a putative ARVC patient. The American College of Medical Genetics and Genomics explicitly warns against relying on their guidelines for interpretation of pathogenicity in genes of unknown significance[45]. Re-evaluation of the patient and family for features suggesting an alternate clinical diagnosis may be useful and the full associated clinical spectrum (eg. desminopathy, laminopathy, CPVT) should inform medical care and familial cascade screening. In the absence of evidence suggesting an alternate diagnosis, reevaluating the pathogenicity of the variant may be warranted.

A few percent of ARVC patients have multiple P/LP variants in strong/moderate evidence ARVC genes, leading to earlier and more severe ARVC manifestations. The rare patients with definite ARVC and also definitively pathogenic variants in limited-evidence genes typically associated with other cardiovascular diseases are particularly likely to harbor additional

American Heart Association.
Circulation: Genomic and Precision Medicine

This article is published in its accepted form; it has not been copyedited and has not appeared in an issue of the journal. Preparation for inclusion in an issue of *Circulation: Genomic and Precision Medicine* involves copyediting, typesetting, proofreading, and author review, which may lead to differences between this accepted version of the manuscript and the final published version.

genetic variants. Real harm can be done by cascade testing of a variant which does not (fully) explain the disease in a family. These second variants in other cardiomyopathy or arrhythmia related genes may also drive the phenotype towards an atypical yet severe, manifestation of ARVC. This underscores that while ARVC is not a condition with substantial genetic heterogeneity, the paradigm of one gene-one disease is challenged[46]. Evidence suggests oligogenic inheritance with multiple additional variants that may or may not reach the LP/P status (or in genes that are not "definitive" for ARVC) could also contribute to disease expression.

Furthermore, recent publications show at least one-third of ARVC cases are gene elusive[12] and these patients are disproportionately high-level athletes with no family history of ARVC, pointing to exercise as contributing to etiology[47]. Among relatives of ARVC patients with P/LP desmosomal variants, exercise increases penetrance and risk of incident arrhythmias, but not all athletic relatives develop ARVC[4848]. Additionally, a recent study[49] showed 0.23% of a general clinical population harbored a loss of function desmosomal variant. These patients had extremely low ARVC penetrance (estimated at 6%) and were no more likely than controls to have ECG or echocardiography findings that met TFC. Taken together this evidence strongly suggests a threshold model of ARVC pathogenesis in which multiple hits, both environmental and genetic, are required for disease expression[2]. Thus, while detection of a P/LP variant in a definitive or moderate evidence gene in a patient with ARVC features is highly indicative of disease, it does not fully predict the clinical features or course of individual patients. The fact that these different aspects influence disease expression, and are not accounted for in the ClinGen framework which is built around true penetrant Mendelian disease, can be considered a

Circulation: Genomic and Precision Medicine

This article is published in its accepted form; it has not been copyedited and has not appeared in an issue of the journal. Preparation for inclusion in an issue of *Circulation: Genomic and Precision Medicine* involves copyediting, typesetting, proofreading, and author review, which may lead to differences between this accepted version of the manuscript and the final published version.

limitation and are important to keep in mind when using the results of this analysis to interpret genetic tests.

In summary, substantial caution is required for the interpretation of variants in limited evidence genes which are unlikely to be the sole cause of disease in a patient with ARVC. Our results suggest such variants should not be used to assign the patient a major TFC criterion.

Panel selection for genetic testing

This study also challenges the inclusion of several genes frequently present in ARVC panels – foremost among them *RYR2*. Our results identify genes with definitive and moderate evidence for ARVC and show that most P/LP variants in ARVC patients occur in these genes. However, a clinical ARVC diagnosis can be challenging, particularly in early stages of disease where structural abnormalities can be subtle but arrhythmic risk is nonetheless significant[1]. Careful use of a larger panel can therefore facilitate correct genetic diagnosis of a family. Using a large panel responsibly in this context requires multidisciplinary expertise[6].

American Heart Association.
Circulation: Genomic and Precision Medicine

Limitations

Genes were curated for ARVC per 2010 TFC. Updated diagnostic criteria are being considered, particularly for the left-dominant form of ARVC[50], thus a need to update ARVC curation is anticipated.

Although genes accounting for most familial ARVC have been identified, expanded sequencing efforts and new analytic approaches will identify rare or family-specific variants in novel putative ARVC that will require adjudication (foremost among these *FLNC*). Recuration of limited and moderate evidence genes will be done per ClinGen procedures as new data emerges. (https://clinicalgenome.org/site/assets/files/2164/clingen_standard_gene-disease_validity_recuration_procedures_v1.pdf).

This article is published in its accepted form; it has not been copyedited and has not appeared in an issue of the journal. Preparation for inclusion in an issue of *Circulation: Genomic and Precision Medicine* involves copyediting, typesetting, proofreading, and author review, which may lead to differences between this accepted version of the manuscript and the final published version.

Conclusion

This evidence-based re-evaluation of published ARVC genes by experts in the field shows that only a small number of genes, (*PKP2*, *DSP*, *DSG2*, *DSC2*, *JUP*, *TMEM43*, *PLN*, *DES*) are definitively or moderately associated with ARVC and these genes account for the overwhelming majority of P/LP variants in patients with ARVC. We recommend only P/LP variants in these eight genes should yield a major criterion for ARVC diagnosis by TFC. This analysis is expected to further refine the utility of genetic data in caring for families with ARVC by assisting the clinician in determining what test to order and also by quantifying the strength of evidence underlying the gene:disease relationship relevant to a genetic result.

American Heart Association.
Circulation: Genomic and Precision Medicine

Sources of Funding: This work was financially supported by grants from the NIH (U41HG009650) and by the Netherlands Cardiovascular Research Initiative, (PvT) an initiative supported by the Dutch Heart Foundation (CVON2018-30 PREDICT2 and CVON 2015-12 eDETECT). The Johns Hopkins ARVD/C Program (CAJ, BM) is supported by the Leonie-Wild Foundation, the Leyla Erkan Family Fund for ARVD Research, the Dr. Satish, Rupal, and Robin Shah ARVD Fund at Johns Hopkins, the Bogle Foundation, the Healing Hearts Foundation, the Campanella family, the Patrick J. Harrison Family, the Peter French Memorial Foundation, and the Wilmerding Endowments. PS was supported by Fondation Leducq Transatlantic Networks of Excellence Program grant no 14CVD03 and the National Institute for Health Research University College London Hospitals Biomedical Research Centre (UK). AP is supported by a British Heart Foundation clinical research training fellowship grant (FS/18/82/34024).

Disclosures: BM and EB are consultants for MyGeneCounsel.

Supplemental Material

Supplementary Methods

Supplementary Tables I-IV

References [51-52]

This article is published in its accepted form; it has not been copyedited and has not appeared in an issue of the journal. Preparation for inclusion in an issue of *Circulation: Genomic and Precision Medicine* involves copyediting, typesetting, proofreading, and author review, which may lead to differences between this accepted version of the manuscript and the final published version.

DOI: 10.1161/CIRCGEN.120.003273

References:

1. Corrado D, Link MS, Calkins H. Arrhythmogenic Right Ventricular Cardiomyopathy. *N Engl J Med*. 2017;376:1489-1490.

2. James CA, Syrris P, van Tintelen JP, Calkins H. The role of genetics in cardiovascular disease: arrhythmogenic cardiomyopathy. *Eur Heart J.* 2020;41:1393-1400.

3. McKoy G, Protonotarios N, Crosby A, Tsatsopoulou A, Anastasakis A, Coonar A, Norman M, Baboonian C, Jeffrey S, McKenna WJ. Identification of a deletion in plakoglobin in arrhythmogenic right ventricular cardiomyopathy with palmoplantar keratoderma and woolly hair (Naxos disease). *Lancet*. 2000;355:2119-2124.

4. Groeneweg JA, Bhonsale A, James CA, te Riele AS, Dooijes D, Tichnell C, Murray B, Wiesfeld AC, Sawant AC, Kassamali B, et al. Clinical presentation, long-term follow-up, and outcomes of 1001 arrhythmogenic right ventricular dysplasia/cardiomyopathy patients and family members. *Circ Cardiovasc Genet.* 2015;8:437-446.

5. Marcus FI, McKenna WJ, Sherrill D, Basso C, Bauce B, Bluemke DA, Calkins H, Corrado D, Cox MG, Steinberg JS, et al. Diagnosis of arrhythmogenic right ventricular cardiomyopathy/dysplasia: proposed modification of the task force criteria. *Circulation*. 2010;121:1533-1541.

6. Towbin JA, McKenna WJ, Abrams DJ, Ackerman MJ, Calkins H, Darrieux FCC, Daubert JP, de Chiliou C, DePasquale EC, Desai MY, et al. 2019 HRS expert consensus statement on evaluation, risk stratification, and management of arrhythmogenic cardiomyopathy. *Heart Rhythm*. 2019;16:e301-e372.

7. Strande NT, Riggs ER, Buchanan AH, Ceyhan-Birsoy O, DiStefano M, Dwight SS, Goldstein J, Ghosh R, Seifert BA, Sneddon TP, et al. Evaluating the clinical validity of gene-disease associations: An evidence-based framework developed by the clinical genome resource. *Am J Hum Genet.* 2017;100:895-906.

8. Ingles J, Goldstein J, Thaxton C, Caleshu C, Corty EW, Crowley SB, Dougherty K, Harrison SM, McGlaughon J, Milko LV, et al. Evaluating the Clinical Validity of Hypertrophic Cardiomyopathy Genes. *Circ Genom Precis Med.* 2019;12:e002460.

9. Adler A, Novelli V, Amin AS, Abiusi E, Care M, Nannenberg EA, Feilotter H, Amenta S, Mazza D, Bikker H, et al. An International, Multicentered, Evidence-Based Reappraisal of Genes Reported to Cause Congenital Long QT Syndrome. *Circulation*. 2020;141:418-428.

10. Hosseini SM, Kim R, Udupa S, Costain G, Jobling R, Liston E, Jamal SM, Szybowska M, Morel CF, Bowdin S, et al. Reappraisal of Reported Genes for Sudden Arrhythmic Death: Evidence-Based Evaluation of Gene Validity for Brugada Syndrome. *Circulation*. 2018;138:1195-1205.

American Heart Association.

Circulation: Genomic and Precision Medicine

This article is published in its accepted form; it has not been copyedited and has not appeared in an issue of the journal. Preparation for inclusion in an issue of *Circulation: Genomic and Precision Medicine* involves copyediting, typesetting, proofreading, and author review, which may lead to differences between this accepted version of the manuscript and the final published version.

11. Elliott PM, Anastasakis A, Asimaki A, Basso C, Bauce B, Brooke MA, Calkins H, Corrado D, Duru F, Green KJ, et al. Definition and treatment of arrhythmogenic cardiomyopathy: an updated expert panel report. *Eur J Heart Fail.* 2019;21:955-964.

12. Corrado D, Perazzolo MM, Zorzi A, Beffagna G, Cipriani A, De Lazzari M, Migliore F, Pilichou K, Rampazzo A, Rigato I, et al. Diagnosis of arrhythmogenic cardiomyopathy: The Padua criteria. *Int J Cardiol.* 2020;319:106-114.

13. Milting H, Klauke B, Christensen AH, Musebeck J, Walhorn V, Grannemann S, Münnich S, Šarić T, Rasmussen TB, Jensen HK, et al. The TMEM43 Newfoundland mutation p.S358L causing ARVC-5 was imported from Europe and increases the stiffness of the cell nucleus. *Eur Heart J.* 2015;36:872-881.

14. Dominguez F, Zorio E, Jimenez-Jaimez J, Salguero-Bodes R, Zwart R, Gonzalez-Lopez E, Molina P, Bermudez-Jiménez, Delgado JF, Braza-Boïls, et al. Clinical characteristics and determinants of the phenotype in TMEM43 arrhythmogenic right ventricular cardiomyopathy type 5. *Heart Rhythm.* 2020;17:945-954.

15. Merner ND, Hodgkinson KA, Haywood AF, Connors S, French VM, Drenckhahn JD, Kupprion C, Ramadanova K, Thierfelder L, McKenna W, et al. Arrhythmogenic right ventricular cardiomyopathy type 5 is a fully penetrant, lethal arrhythmic disorder caused by a missense mutation in the TMEM43 gene. *Am J Hum Genet.* 2008;82:809-821.

16. van der Zwaag, PA, van Rijsingen IA, de Ruiter R, Nannenberg EA, Groeneweg JA, Post JG, Hauer RN, van Gelder IC, Van den Berg MP, van der Harst P, et al. Recurrent and founder mutations in the Netherlands-Phospholamban p.Arg14del mutation causes arrhythmogenic cardiomyopathy. *Neth Heart J.* 2013;21:286-293.

17. van Spaendonck-Zwarts KY, van Hessem L, Jongbloed JD, de Walle HE, Capetanaki Y, van der Kooi, AJ, van Langen IM, van den Berg MP, van Tintelen JP. Desmin-related myopathy. *Clin Genet.* 2011;80:354-366.

18. Klauke B, Kossmann S, Gaertner A, Brand K, Stork I, Brodehl A, Dieding M, Walhorn V, Anselmetti D, Gerdes D, et al. De novo desmin-mutation N116S is associated with arrhythmogenic right ventricular cardiomyopathy. *Hum Mol Genet.* 2010;19:4595-4607.

19. Maruthappu T, Posafalvi A, Castelletti S, Delaney PJ, Syrris P, O'Toole EA, Green KJ, Elliott PM, Lambiase PD, Tinker A, et al. Loss-of-function desmoplakin I and II mutations underlie dominant arrhythmogenic cardiomyopathy with a hair and skin phenotype. *Br J Dermatol.* 2019;180:1114-1122.

20. Gerull B, Kirchner F, Chong JX, Tagoe J, Chandrasekharan K, Strohm O, Waggoner D, Ober C, Duff HJ. Homozygous founder mutation in desmocollin-2 (DSC2) causes arrhythmogenic cardiomyopathy in the Hutterite population. *Circ Cardiovasc Genet.* 2013;6:327-336.

Circulation: Genomic and Precision Medicine

This article is published in its accepted form; it has not been copyedited and has not appeared in an issue of the journal. Preparation for inclusion in an issue of *Circulation: Genomic and Precision Medicine* involves copyediting, typesetting, proofreading, and author review, which may lead to differences between this accepted version of the manuscript and the final published version.

21. Chen L, Rao M, Chen X, Chen K, Ren J, Zhang N, Zhao Q, Yu W, Yuian B, Song J. A founder homozygous DSG2 variant in East Asia results in ARVC with full penetrance and heart failure phenotype. *Int J Cardiol.* 2019;274:263-270.

22. Baskin B, Skinner JR, Sanatani S, Terespolsky D, Krahn AD, Ray PN, Scherer SW, Hamilton RM. TMEM43 mutations associated with arrhythmogenic right ventricular cardiomyopathy in non-Newfoundland populations. *Hum Genet.* 2013;132:1245-1252.

23. van Tintelen JP, Van Gelder IC, Asimaki A, Suurmeijer AJ, Wiesfeld AC, Jongbloed JD, van den Wijngaard A, Kuks JB, van Spaendonck-Zwarts KY, Notermans N, et al. Severe cardiac phenotype with right ventricular predominance in a large cohort of patients with a single missense mutation in the DES gene. *Heart Rhythm.* 2009;6:1574-1583.

24. Lorenzon A, Beffagna G, Bauce B, De Bortoli M, Li Mura IE, Calore M, Dazzo E, Basso C, Nava A, Thiene G, et al. Desmin mutations and arrhythmogenic right ventricular cardiomyopathy. *Am J Cardiol.* 2013;111:400-405.

25. Hedberg C, Melberg A, Kuhl A, Jenne D, Oldfors A. Autosomal dominant myofibrillar myopathy with arrhythmogenic right ventricular cardiomyopathy 7 is caused by a DES mutation. *Eur J Hum Genet.* 2012;20:984-985.

26. Bermúdez-Jiménez FJ, Carriel V, Brodehl A, Alaminos M, Campos A, Schirmer I, Milting H, Abril BA, Álvarez M, López-Fernández S, et al. Novel desmin mutation p.Glu401Asp impairs filament formation, disrupts dell membrane integrity, and causes severe arrhythmogenic left ventricular cardiomyopathy/dysplasia. *Circulation*. 2018;137:1595-1610.

27. van der Zwaag, PA, van Rijsingen IA, Asimaki A, Jongbloed JD, van Veldhuisen DJ, Wiesfeld AC, Cox MGPJ, van Lochem LT, de Boer RA, Hofstra RMW, et al. Phospholamban R14del mutation in patients diagnosed with dilated cardiomyopathy or arrhythmogenic right ventricular cardiomyopathy: evidence supporting the concept of arrhythmogenic cardiomyopathy. *Eur J Heart Fail.* 2012;14:1199-1207.

28. Sepehrkhouy S, Gho JMIH, van Es R, Harakalova M, de Jonge N, Dooijes D, van der Smagt JJ, Buisrogge MP, Hauer RNW, Goldschmeding R, et al. Distinct fibrosis pattern in desmosomal and phospholamban mutation carriers in hereditary cardiomyopathies. *Heart Rhythm.* 2017;14:1024-1032.

29. Mayosi BM, Fish M, Shaboodien G, Mastantuono E, Kraus S, Wieland T, Kotta M, Chin A, Laing N, Ntusi NBA, et al. Identification of Cadherin 2 (CDH2) mutations in arrhythmogenic right ventricular cardiomyopathy. *Circ Cardiovasc Genet.* 2017;10:e001605.

30. Turkowski KL, Tester DJ, Bos JM, Haugaa KH, Ackerman MJ. Whole exome sequencing with genomic triangulation implicates CDH2-encoded N-cadherin as a novel pathogenic substrate for arrhythmogenic cardiomyopathy. *Congenit Heart Dis.* 2017;12:226-235.

This article is published in its accepted form; it has not been copyedited and has not appeared in an issue of the journal. Preparation for inclusion in an issue of *Circulation: Genomic and Precision Medicine* involves copyediting, typesetting, proofreading, and author review, which may lead to differences between this accepted version of the manuscript and the final published version.

31. van Hengel J, Calore M, Bauce B, Dazzo E, Mazzotti E, De Bortoli M, Lorenzon A, Li Mura IEA, Beffanga G, Rigato I, et al. Mutations in the area composita protein alphaT-catenin are associated with arrhythmogenic right ventricular cardiomyopathy. *Eur Heart J*. 2013;34:201-210.

32. De Bortoli M, Postma AV, Poloni G, Calore M, Minervini G, Mazzotti E, Rigato I, Ebert M, Lorenzon A, Vazza G, et al. Whole-exome sequencing identifies pathogenic variants in TJP1 gene associated with arrhythmogenic cardiomyopathy. *Circ Genom Precis Med*. 2018;11:e002123.

33. Te Riele AS, Agullo-Pascual E, James CA, Leo-Macias A, Cerrone M, Zhang M, et al. Multilevel analyses of SCN5A mutations in arrhythmogenic right ventricular dysplasia/cardiomyopathy suggest non-canonical mechanisms for disease pathogenesis. *Cardiovasc Res.* 2017;113:102-111.

34. Quarta G, Syrris P, Ashworth M, Jenkins S, Zuborne Alapi K, Morgan J, Muir A, Pantazis A, McKenna WJ, Elliott PM. Mutations in the Lamin A/C gene mimic arrhythmogenic right ventricular cardiomyopathy. *Eur Heart J.* 2012;33:1128-1136.

35. Forleo C, Carmosino M, Resta N, Rampazzo A, Valecce R, Sorrentino S, Iacoviello M, Pisani F, Procino G, Gerbino A, et al. Clinical and functional characterization of a novel mutation in lamin a/c gene in a multigenerational family with arrhythmogenic cardiac laminopathy. *PLoS One*. 2015;10:e0121723.

36. Beffagna G, Occhi G, Nava A, Vitiello L, Ditadi A, Basso C, Bauce B, Carraro G, Thiene G, Towbin JA, et al. Regulatory mutations in transforming growth factor-beta3 gene cause arrhythmogenic right ventricular cardiomyopathy type 1. *Cardiovasc Res*. 2005;65:366-373.

37. Marsili L, Overwater E, Hanna N, Baujat G, Baars MJH, Boileau C, Bonneau D, Brehin AC Yline C, Cheung HY, et al. Phenotypic spectrum of TGFB3 disease-causing variants in a Dutch-French cohort and first report of a homozygous patient. *Clin Genet.* 2020;97:723-730.

38. Chen K, Song J, Wang Z, Rao M, Chen L, Hu S. Absence of a primary role for TTN missense variants in arrhythmogenic cardiomyopathy: From a clinical and pathological perspective. *Clin Cardiol.* 2018;41:615-622.

39. Taylor M, Graw S, Sinagra G, Barnes C, Slavov D, Brun F, Pinamonti B, Salcedo EE, Sauer W, Pyxaras S, et al. Genetic variation in titin in arrhythmogenic right ventricular cardiomyopathy-overlap syndromes. *Circulation.* 2011;124:876-885.

40. Gigli M, Merlo M, Graw SL, Barbati G, Rowland TJ, Slavov DB, Stolfo D, Haywood ME, Dal Ferro M, Altinier A, et al. Genetic risk of arrhythmic phenotypes in patients with dilated cardiomyopathy. *J Am Coll Cardiol.* 2019;74:1480-1490.

41. Murray B, Hoorntje ET, Te Riele, ASJM, Tichnell C, van der Heijden, JF, Tandri H, van den Berg MP, Jongbloed JDH, Wilde AAM, Hauer RNW, et al. Identification of sarcomeric variants

Circulation: Genomic and Precision Medicine

This article is published in its accepted form; it has not been copyedited and has not appeared in an issue of the journal. Preparation for inclusion in an issue of *Circulation: Genomic and Precision Medicine* involves copyediting, typesetting, proofreading, and author review, which may lead to differences between this accepted version of the manuscript and the final published version.

in probands with a clinical diagnosis of arrhythmogenic right ventricular cardiomyopathy (ARVC). *J Cardiovasc Electrophysiol.* 2018;29:1004-1009.

42. Tiso N, Stephan DA, Nava A, Bagattin A, Devaney JM, Stanchi F, Larderet G, Brahmbhatt B, Brown K, Baucer B, et al. Identification of mutations in the cardiac ryanodine receptor gene in families affected with arrhythmogenic right ventricular cardiomyopathy type 2 (ARVD2). *Hum Mol Genet.* 2001;10:189-194.

43. Kannankeril PJ, Mitchell BM, Goonasekera SA, Chelu MG, Zhang W, Sood S, Kearney DL, Danila CI, De Biasi M, Wehrens XHT, et al. Mice with the R176Q cardiac ryanodine receptor mutation exhibit catecholamine-induced ventricular tachycardia and cardiomyopathy. *Proc Natl Acad Sci USA.* 2006;103:12179-12184.

44. Bhuiyan ZA, van den Berg, M P, van Tintelen JP, Bink-Boelkens MT, Wiesfeld AC, Alders M, Potsma AV, van Langen I, Mannens MMAM, Wilde AAM. Expanding spectrum of human RYR2-related disease: new electrocardiographic, structural, and genetic features. *Circulation.* 2007;116:1569-1576.

45. Richards S, Aziz N, Bale S, Bick D, Das S, Gastier-Foster J, Grody WW, Hegde M, Lyon E, Spector E, et al. Standards and guidelines for the interpretation of sequence variants: a joint consensus recommendation of the American College of Medical Genetics and Genomics and the Association for Molecular Pathology. *Genet Med.* 2015;17:405-424.

46. Cerrone M, Remme CA, Tadros R, Bezzina CR, Delmar M. Beyond the one gene-one disease paradigm: complex genetics and pleiotropy in inheritable cardiac disorders. *Circulation*. 2019;140:595-610.

47. Sawant AC, Bhonsale A, te Riele AS, Tichnell C, Murray B, Russell SD, Tandri H, Tedford RJ, Judge DP, Calkins H, et al. Exercise has a disproportionate role in the pathogenesis of arrhythmogenic right ventricular dysplasia/cardiomyopathy in patients without desmosomal mutations. *J Am Heart Assoc.* 2014;3:e001471.

48. Wang W, Tichnell C, Murray BA, Agafonova J, Cadrin-Tourigny J, Chelko S, Tandri H, Calkins H, James CA. Exercise restriction is protective for genotype-positive family members of arrhythmogenic right ventricular cardiomyopathy patients. *Europace.* 2020;22:1270-1278.

49. Carruth ED, Young W, Beer D, James CA, Calkins H, Jing L, Raghunath S, Hartzel DN, Leader JB, Kirchner HL, et al. Prevalence and electronic health record-based phenotype of loss-of-function genetic variants in arrhythmogenic right ventricular cardiomyopathy-associated genes. *Circ Genom Precis Med*. 2019;12:e002579.

50. Corrado D, van Tintelen PJ, McKenna WJ, Hauer RNW, Anastastakis A, Asimaki A, Basso C, Bauce B, Brunckhorst C, Bucciarelli-Ducci C, et al. Arrhythmogenic right ventricular cardiomyopathy: evaluation of the current diagnostic criteria and differential diagnosis. *Eur Heart J.* 2020;41:1414-1429.

Circulation: Genomic and Precision Medicine

This article is published in its accepted form; it has not been copyedited and has not appeared in an issue of the journal. Preparation for inclusion in an issue of *Circulation: Genomic and Precision Medicine* involves copyediting, typesetting, proofreading, and author review, which may lead to differences between this accepted version of the manuscript and the final published version.

51. van Lint FHM, Murray B, Tichnell C, Zwart R, Amat N, Lekanne Deprez RH, Dittmann S, Stallmeyer B, Calkins H, van der Smagt JJ, et al. Arrhythmogenic right ventricular cardiomyopathy-associated desmosomal variants are rarely de novo. *Circ Genom Precis Med.* 2019:12:e002467.

52. Whiffin N, Minikel E, Walsh R, O'Donnell-Luria AH, Karczewski K, Ing AY, Barton PJR, Funke B, Cook SA, MacArthur D, et al. Using high-resolution variant frequencies to empower clinical genome interpretation. *Genet Med*. 2017;19:1151-1158.

American Heart Association.

Circulation: Genomic and Precision Medicine

This article is published in its accepted form; it has not been copyedited and has not appeared in an issue of the journal. Preparation for inclusion in an issue of *Circulation: Genomic and Precision Medicine* involves copyediting, typesetting, proofreading, and author review, which may lead to differences between this accepted version of the manuscript and the final published version.

DOI: 10.1161/CIRCGEN.120.003273

Table 1: Reported Genes for ARVC

Gene	Protein	Cellular complex	HGNC ID*
ACTC1	actin alpha cardiac muscle 1	sarcomere	143
CDH2	cadherin-2	area composita	1759
CTNNA3	catenin alpha 3	area composita	2511
DES	desmin	intermediate filament	2770
DSC2	desmocollin-2	desmosome	3036
DSP	desmoplakin	desmosome	3052
DSG2	desmoglein-2	desmosome	3049
JUP	junction plakoglobin	desmosome	6207
LDB3	LIM domain binding 3	sarcomere	15710
LMNA	lamin A/C	nuclear envelope	6636
MYBPC3	myosin binding protein C3	sarcomere	7551
MYH7	myosin heavy chain 7	sarcomere	7577
MYL2	myosin light chain 2	sarcomere	7583
MYL3	myosin light chain 3	sarcomere	7584
PKP2	plakophilin-2	desmosome	9024
PLN	phospholamban	calcium handling	9080
RYR2	ryanodine receptor 2	calcium handling	10484
SCN5A	sodium voltage-gated channel alpha subunit 5	sodium channel	10593
TGFB3	transforming growth factor beta 3	signaling pathways	11769
TJP1	tight junction protein 1	area composita	11827
TMEM43	transmembrane protein 43	nuclear envelope	28472
TNNI3	troponin I3	sarcomere	11947
TNNC1	troponin C1	sarcomere	11943
TNNT2	troponin T2	sarcomere	11949
TPM1	tropomyosin 1	sarcomere	12010
TTN	titin	sarcomere-related	12403

* HUGO Gene Nomenclature Committee at the European Bioinformatics Institute ID

Circulation: Genomic and Precision Medicine
American Heart Association.
This article is published in its accepted form; it has not been copyedited and has not appeared in an issue of the journal. Preparation for inclusion in an issue of *Circulation: Genomic and Precision Medicine* involves copyediting, typesetting, proofreading, and author review, which may lead to differences between this accepted version of the manuscript and the final published version.

DOI: 10.1161/CIRCGEN.120.003273

Table 2: Genetic architecture of ARVC – Final expert panel classification (https://clinicalgenome.org/)

**Gene***	**Evidence** Genetic	**Evidence** experimental	**Evidence total**	**Prevalence ***	**Inheritance**	**Phenotype notes**
**Definitive**						
PKP2	12	6	18	++++	AD	Classic ARVC
DSP	12	2.5	14.5	+++	AD / AR	Frequent left-ventricular involvement.<br>Occasional hair and skin features.<br>Rare homozygous variants - Carvajal Syndrome (cardioectodermal)
DSG2	12	6	18	+++	AD / AR	Frequent left-ventricular involvement
DSC2	8.5	5.5	14	++	AD / AR	ARVC
JUP	10.5	5.5	16	+ (higher in Naxos, Greece)	AR	Naxos disease (cardioectodermal)
TMEM43	8.1	5	13.1	++ (higher in Newfoundland)	AD	Extremely high sudden death rates in males.
**Moderate**						
PLN	8	3	11	++ (higher in Netherlands)	AD	Frequent left ventricular involvement.<br>Also associated with dilated cardiomyopathy.
DES	6	3.5	9.5	+	AD	Conduction system abnormalities common.<br>Skeletal myopathy possible.<br>Definitive evidence gene for desminopathy

AD: Autosomal dominant, AR: Autosomal recessive / digenic / compound heterozygote, NA:Not available
*Number of "+" corresponds to prevalence of variants in this gene associated with definite ARVC cases

Circulation: Genomic and Precision Medicine

DOI: 10.1161/CIRCGEN.120.003273

Figure Legends:

Figure 1. ARVC gene curation approach. Two-member teams conducted blinded independent dual curation using the semi-quantitative ClinGen framework with ARVC-specific rules for required minor allele frequency of variants detected in patients and phenotypic evaluation of model systems. Each summarized their analysis in separate presentations for the entire ARVC Gene Curation Expert panel who arrived by consensus at the final gene classification.
Abbreviations: GCEP: ARVC gene curation expert panel

Figure 2. Level of evidence scores for genes reported for ARVC. Final genetic (dark blue) and experimental (light blue) evidence scores for 26 genes reported in the literature as associated with ARVC. Only 8 genes had strong or moderate evidence for ARVC causality. The granular scores for each gene along with a complete list of references used are available in Supplementary Table IV.

Circulation: Genomic and Precision Medicine

Figure 3. Variants in ClinVar in ARVC GCEP curated genes. A) Distribution of variants in each gene curated - pathogenic and likely pathogenic variants (blue) were reported primarily in genes encoding the cardiac desmosome. B) Nearly all pathogenic and likely pathogenic variants reported in ClinVar for ARVC are in the genes categorized as definitive or moderate evidence ARVC genes while refuted/disputed/limited evidence genes account for a higher proportion of VUS and benign/likely benign variants.
Abbreviations: B/LB: benign or likely benign; P/LP: pathogenic or likely pathogenic VUS; variant of uncertain significance; GCEP: Gene curation expert panel.

This article is published in its accepted form; it has not been copyedited and has not appeared in an issue of the journal. Preparation for inclusion in an issue of *Circulation: Genomic and Precision Medicine* involves copyediting, typesetting, proofreading, and author review, which may lead to differences between this accepted version of the manuscript and the final published version.

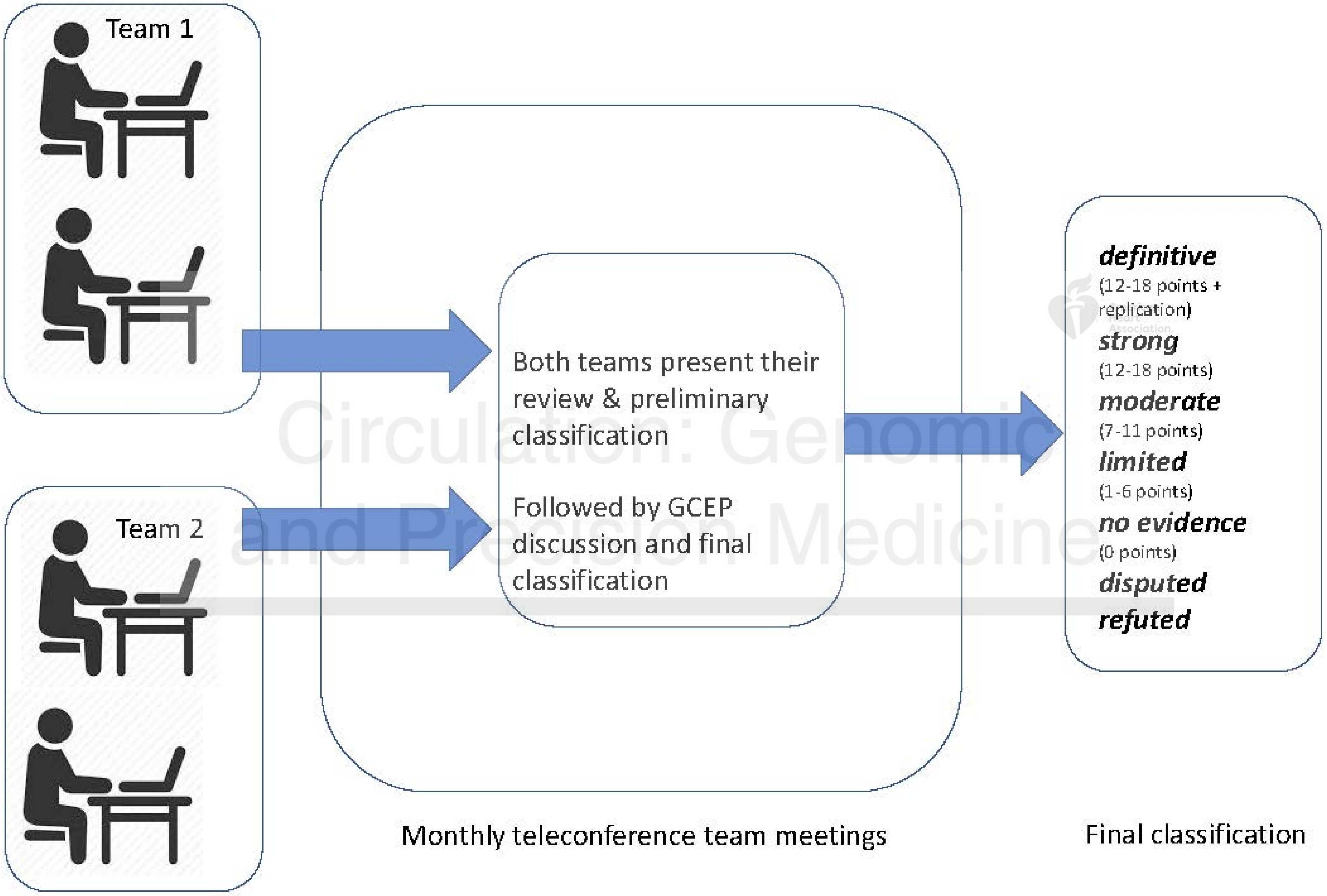

Circulation: Genomic and Precision Medicine

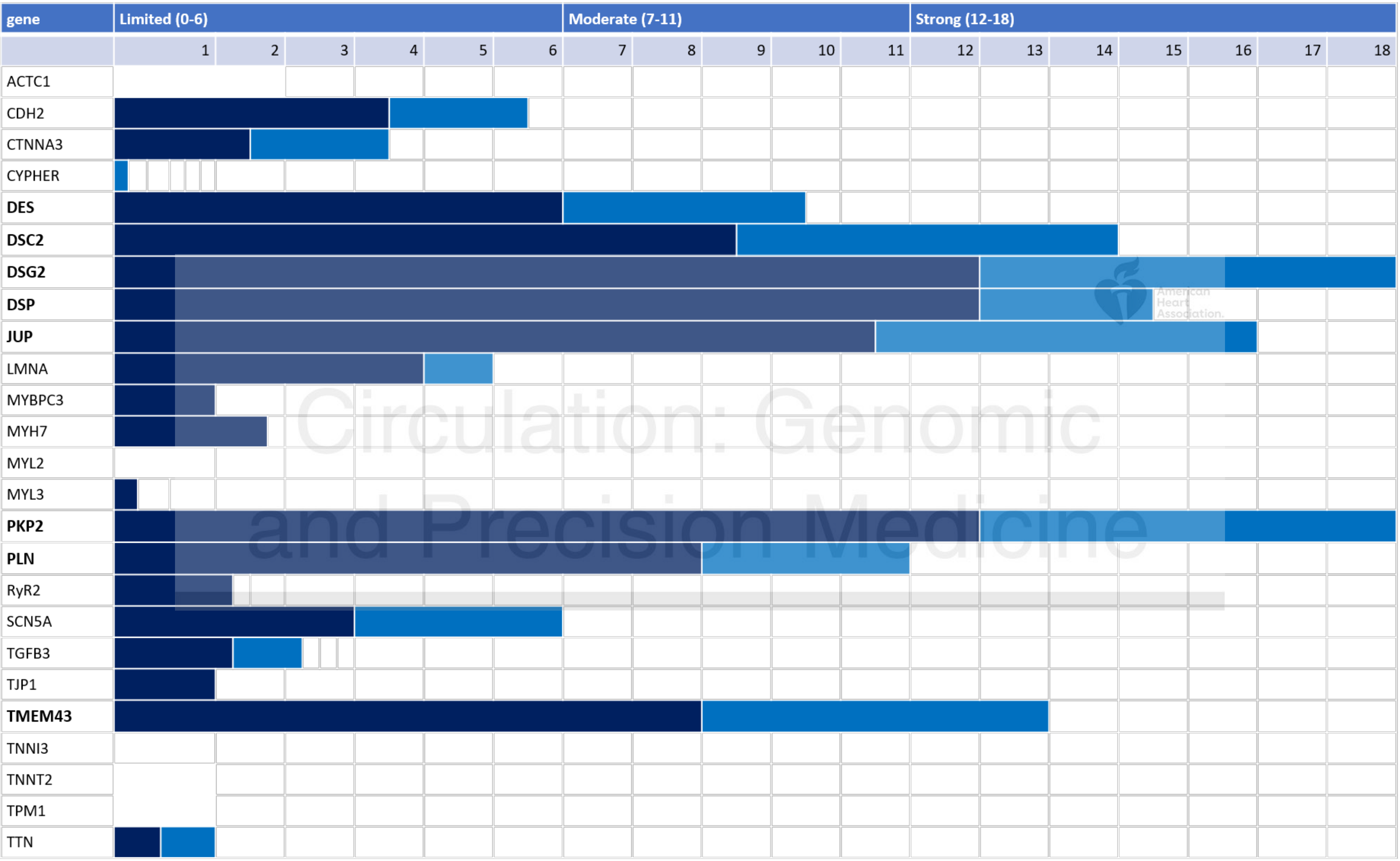

Circulation: Genomic and Precision Medicine
American Heart Association.

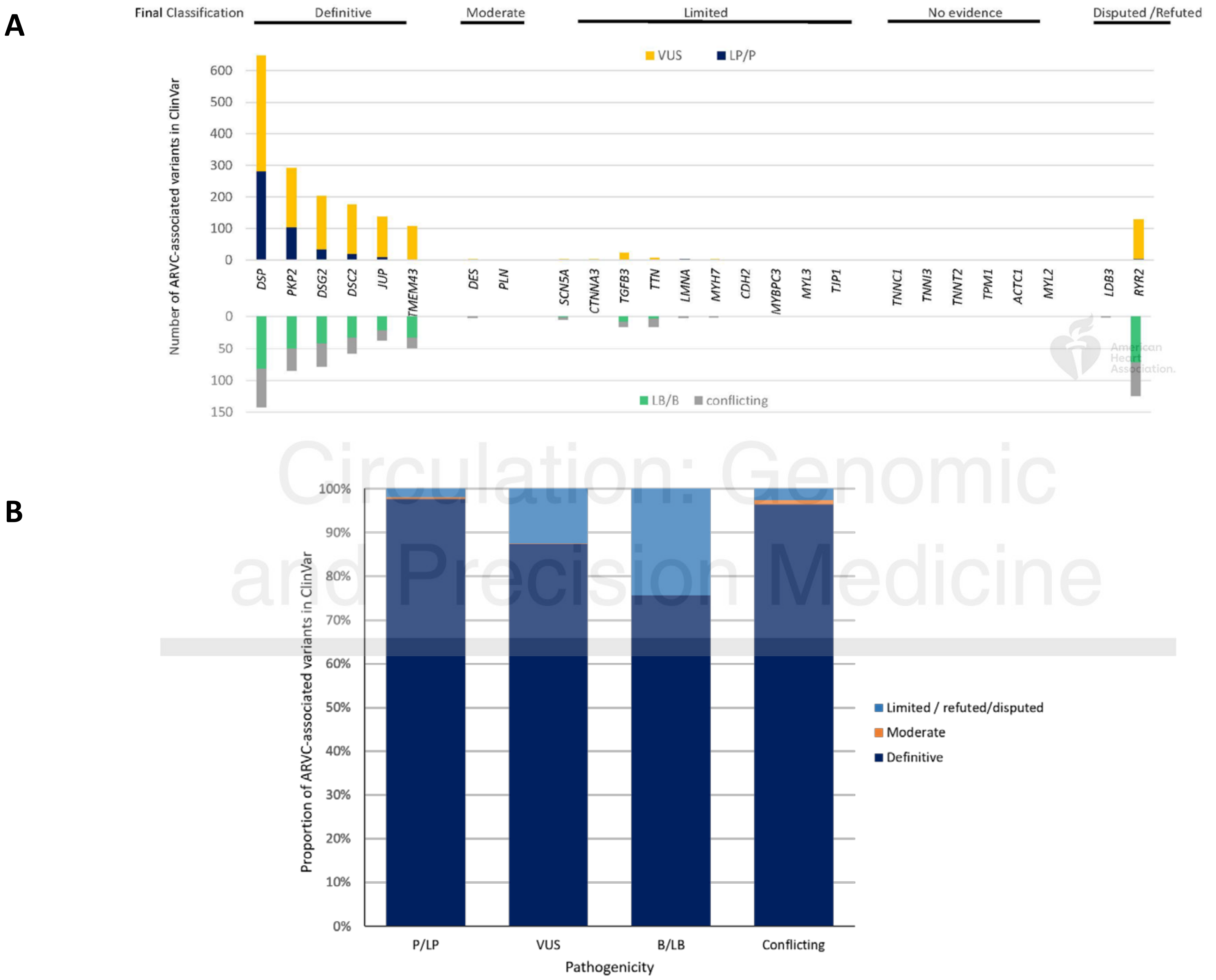

Circulation: Genomic and Precision Medicine
American Heart Association.
